# Regulation of tryptophan-indole metabolic pathway in *Porphyromonas gingivalis* virulence and microbiota dysbiosis in periodontitis

**DOI:** 10.1038/s41522-025-00669-y

**Published:** 2025-02-27

**Authors:** Jing Ding, Lingping Tan, Lingzhi Wu, Jinyu Li, Yong Zhang, Zongshan Shen, Chi Zhang, Chuanjiang Zhao, Li Gao

**Affiliations:** 1 Hospital of Stomatology, Sun Yat-sen University, Guangzhou, China; 2 Guangdong Provincial Key Laboratory of Stomatology, Guangzhou, China; 3 Guanghua School of Stomatology, Sun Yat-sen University, Guangzhou, China

**Keywords:** Plaque, Pathogens, Biofilms, Microbiome

## Abstract

Pathogenesis of periodontitis is marked by microbiota dysbiosis and disrupted host responses. *Porphyromonas gingivalis* is a keystone pathogen of periodontitis which expresses various crucial virulence factors. This study aimed to clarify the role and mechanisms of *P. gingivalis* tryptophan-indole metabolic pathway in the pathogenesis of periodontitis. This study showed that periodontitis patients exhibited elevated tryptophan metabolism and salivary pathogen abundance. Tryptophanase gene-deficiency altered proteome and metabolome of *P. gingivalis,* inhibited *P. gingivalis* virulent factors expression, biofilm growth, hemin utilization, cell adhesion/invasion and pro-inflammation ability. Tryptophan-indole pathway of *P. gingivalis* stimulated periodontitis biofilm formation and induced oral microbiota dysbiosis. In periodontitis mice, this pathway of *P. gingivalis* aggravated alveolar bone loss and gingival tissue destruction, causing oral and gut microbiota dysbiosis. This study indicates that the tryptophan-indole pathway serves as a significant regulator of *P. gingivalis* virulence and oral microbiota dysbiosis, which is also associated with gut dysbiosis.

## Introduction

Periodontitis is a chronic inflammatory disease caused by oral pathogens, leading to irreversible damage to alveolar bone and gingival tissue, and is the main cause of tooth loss worldwide^1^. The onset and progression of periodontitis are centered around microbiota dysbiosis and a disrupted immune-inflammatory response in the subgingival environment, where the colonization and proliferation of keystone pathogens below the gumline disrupted host-microbe homeostasis and initiate local inflammation^2^. In the subgingival environment, robust protein and amino acid metabolic activities are identified as the most prominent features of the dysbiotic microbiota, for the pathogenic members of the community were mostly specialized in proteolytic activities, which help them gain nutritional advantages in the inflammatory environment filled with tissue breakdown products^3,4^. Studies of dysbiotic periodontal microbiota have identified a number of functional pathways related to amino acid metabolism in the pathogenic community or individual pathogens^5,6^.

Tryptophan, an essential amino acid, can be catabolized by host or bacterial enzymes into indole and indole derivatives^7^. Disrupted tryptophan metabolism can lead to inflammatory diseases like rheumatoid arthritis and inflammatory bowel diseases^8–11^. Recent researches indicate that tryptophan metabolites, particularly indole from pathogens in inflamed environments, disrupt microbial balance and host responses^12^. Indole from dysbiotic gut microbiota worsens arthritis in mice^10^ and can cause significant colon inflammation when transferred to healthy mice^8^. Upregulated tryptophan metabolism was also reported in the dysbiotic periodontal microbiota, and elevated levels of indole could be detected from the saliva and gingival crevicular fluids of periodontitis patients^11,13^, indicating that disrupted tryptophan metabolism was a functional signature of periodontitis microbiota, and indole metabolites produced by keystone pathogens might contribute to microbiota dysbiosis and host inflammation. Dysbiotic changes of the oral microbiota could also disrupt the homeostasis of downstream microbial community, namely the gut microbiota by ectopic translocation and colonization, where members of the gut microbiota are widely modulated by microbial metabolic cues including indoles and others^14,15^. Indole is a microbial signal generated from microbiota tryptophan metabolism whose regulatory potential in the polymicrobial community and host responses has been highlighted in recent years^16,17^. The degradation of exogenous tryptophan by microbial tryptophanase at the host-microbe interface, which is mainly expressed by oral and gut pathogens, is the sole source of indole in the mammalian host^7,18^. Over the past decade, researchers have confirmed that indole acts as an interspecies signal influencing biofilm formation, virulence factor expression, spore formation, and other pathogen activities in infectious and inflammatory diseases^14,16^. However, the regulatory roles of these pathways in the virulence of periodontal pathogens remain unclear.

*P. gingivalis*, a key pathogen in periodontitis, relies on protein and amino acid metabolism for survival and proliferation. Its pathogenicity is largely due to its production of lipopolysaccharides, fimbria proteins, outer membrane vesicles, and its ability to metabolize proteins and amino acids^19,20^. Previous researches have demonstrated how different amino acid metabolism pathways in *P*. *gingivalis* directly regulated its colonization, translocation, pro-inflammatory ability^21,22^, and interspecies interactions^23,24^. *P. gingivalis* is presumed to possess the capability to catabolize tryptophan into indole as it has been reported to express a homologous tryptophanase gene^25^. However, no study has yet identified the role of the tryptophan-indole metabolic activity in the pathogenicity of this significant pathogen, nor its potential relationship with the gut microbiota. Therefore, this study aims to investigate the tryptophan-indole metabolic pathway in *P. gingivalis* to elucidate its role and mechanism in virulence expression, oral and gut microbiota dysbiosis, and periodontal tissue inflammation.

## Results

### Enhanced tryptophan metabolism of periodontitis patients was associated with periodontal clinical parameters and pathogen abundances

**Fig. 1:**
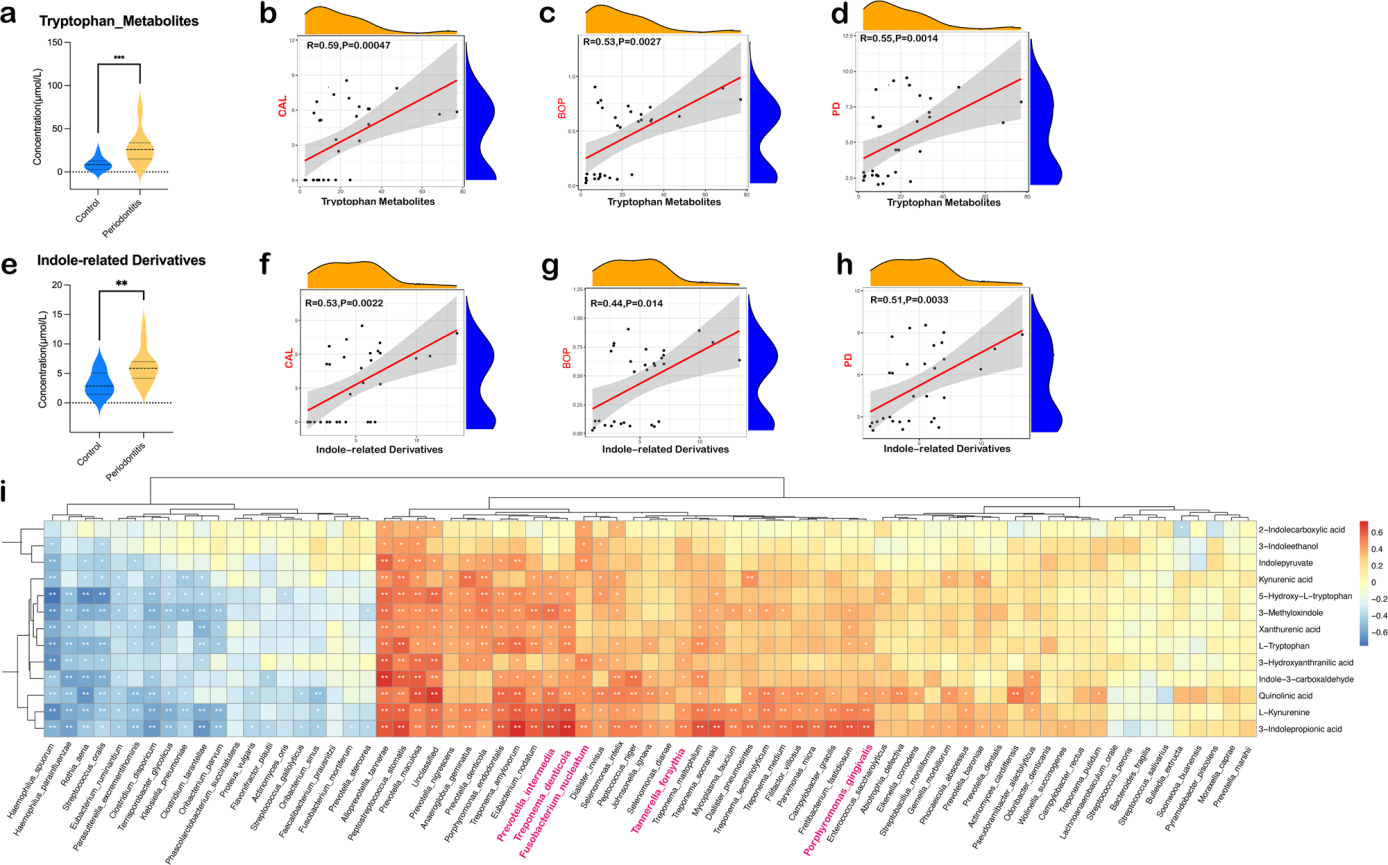
Combined salivary metabolomics and 16S rRNA amplicon analysis of periodontitis patients and healthy individuals. **a** Concentrations of tryptophan-related metabolites in periodontitis patients and healthy individuals. Correlation analysis of the concentrations of tryptophan-related metabolite and periodontal clinical parameters, including CAL (**b**), BOP (**c**) and PD (**d**). **e** Concentrations of indole and its derivatives in periodontitis patients and healthy individuals. Correlation analysis of the concentrations of indole derivatives and periodontal clinical parameters, including CAL (**f**), BOP (**g**) and PD (**h**). **i** Correlation analysis of the tryptophan-related metabolites and the abundances of differential microbial genera in periodontitis patients. Statistical significance was determined using the Kruskal–Wallis *H* test. *p < 0.05, **p < 0.01, ***p < 0.001.

Multi-omics data revealed that patients with stage III or IV periodontitis exhibited enhanced tryptophan metabolism, marked by higher levels of tryptophan-related metabolites and indole derivatives (Fig. 1a, e). A strong positive correlation was also found between the clinical parameters of periodontitis, including clinical attachment loss (CAL), bleeding on probing (BOP) and probing depth (PD), and the concentrations of tryptophan-related metabolites (Fig. 1b–d) or indole derivatives (Fig. 1f–h). This indicates that increased tryptophan metabolism is a key feature of the periodontitis microenvironment and is closely linked to the severity of tissue destruction.

We further found that the increased presence of key periodontal pathogens like *P*. *gingivalis*, *Treponema denticola*, *Tannerella forsythia*, *Prevotella intermedia* and *F*. *nucleatum* in the periodontitis cohort was positively correlated with tryptophan-related metabolites, indicating that the tryptophan-indole metabolic pathway plays a crucial role in the periodontitis environment.

### Tryptophan modulated the protein and amino acid metabolism of *P. gingivalis*

**Fig. 2:**
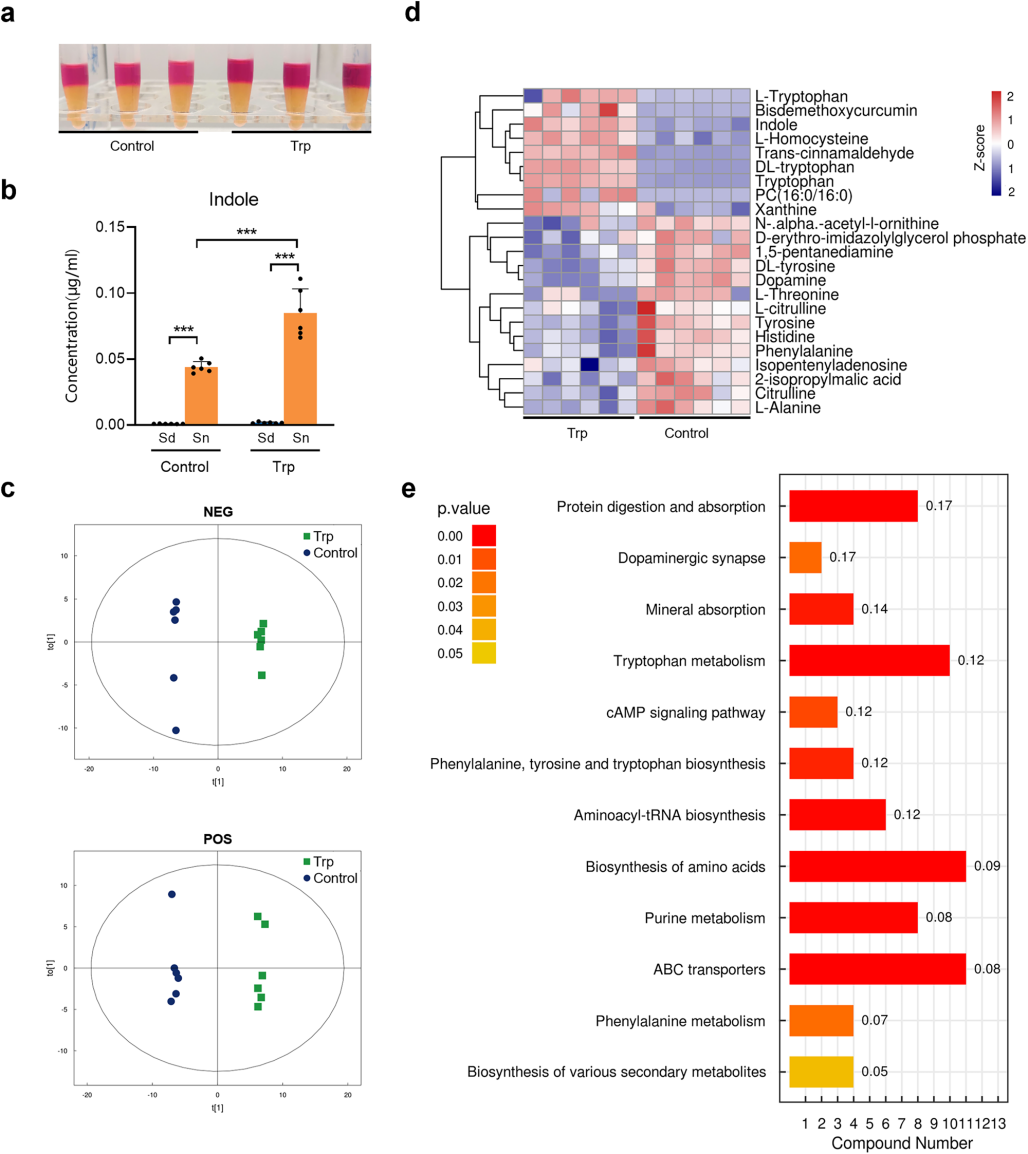
Indole production measurement and metabolomics analysis of *P. gingivalis.* **a** Indole assay of the 24 h-culture of *P. gingivalis* supplemented with tryptophan (3 mM). The 24 h-culture of *P. gingivalis* grown in blank medium was used as control. **b** Intracellular and extracellular indole concentration of *P. gingivalis* after 24-h cultivation with or without tryptophan (3 mM) supplementation determined by liquid chromatography and mass spectrometry. **c** OPLS-DA model showing the separation of *P. gingivalis* metabolome after 24-h cultivation with or without tryptophan (3 mM). **d** Heatmap of key differential metabolites produced by *P. gingivalis* cultured with tryptophan (3 mM) after 24 h. **e** KEGG pathway enrichment analysis of *P. gingivalis* cultured with tryptophan (3 mM) after 24 h. Compound number showed the number of differential metabolites annotated in each pathway. Rich factor was marked on each bar showing the ratio of differential metabolites to all annotated metabolites in every pathway. Sd Sediment, Sn Supernatant. Statistical significance was determined using Student’s *t* test. ***p < 0.001.

As demonstrated in Fig. 2a, *P. gingivalis* exhibited the ability to produce indole. The addition of exogenous tryptophan significantly enhanced the level of indole production in *P. gingivalis*, with the indole products predominantly being released into the extracellular environment (Fig. 2b).

Non-targeted metabolomics was performed to gain insight into the tryptophan-modulated *P. gingivalis* metabolism. The tryptophan-stimulated *P. gingivalis* showed a distinct metabolomic profile compared to the control group (Fig. 2c). Notably, there were significant changes in protein and amino acid metabolites, with indole, L-homocysteine, and xanthine upregulated, and tyrosine, threonine, and citrulline phenylalanine, alanine and histidine downregulated (Fig. 2d and Table S1). Further functional analysis revealed that adding tryptophan to *P. gingivalis* significantly enhanced pathways related to protein digestion, tryptophan metabolism, and ABC transporters (Fig. 2e). This suggests that exogenous tryptophan boosts not only tryptophan metabolism but also other functional activities, particularly the transport of proteins, amino acids, and minerals, potentially affecting its survival and virulence in the microbial community.

### Tryptophanase gene deficiency altered the *P. gingivalis* proteome and metabolome

**Fig. 3:**
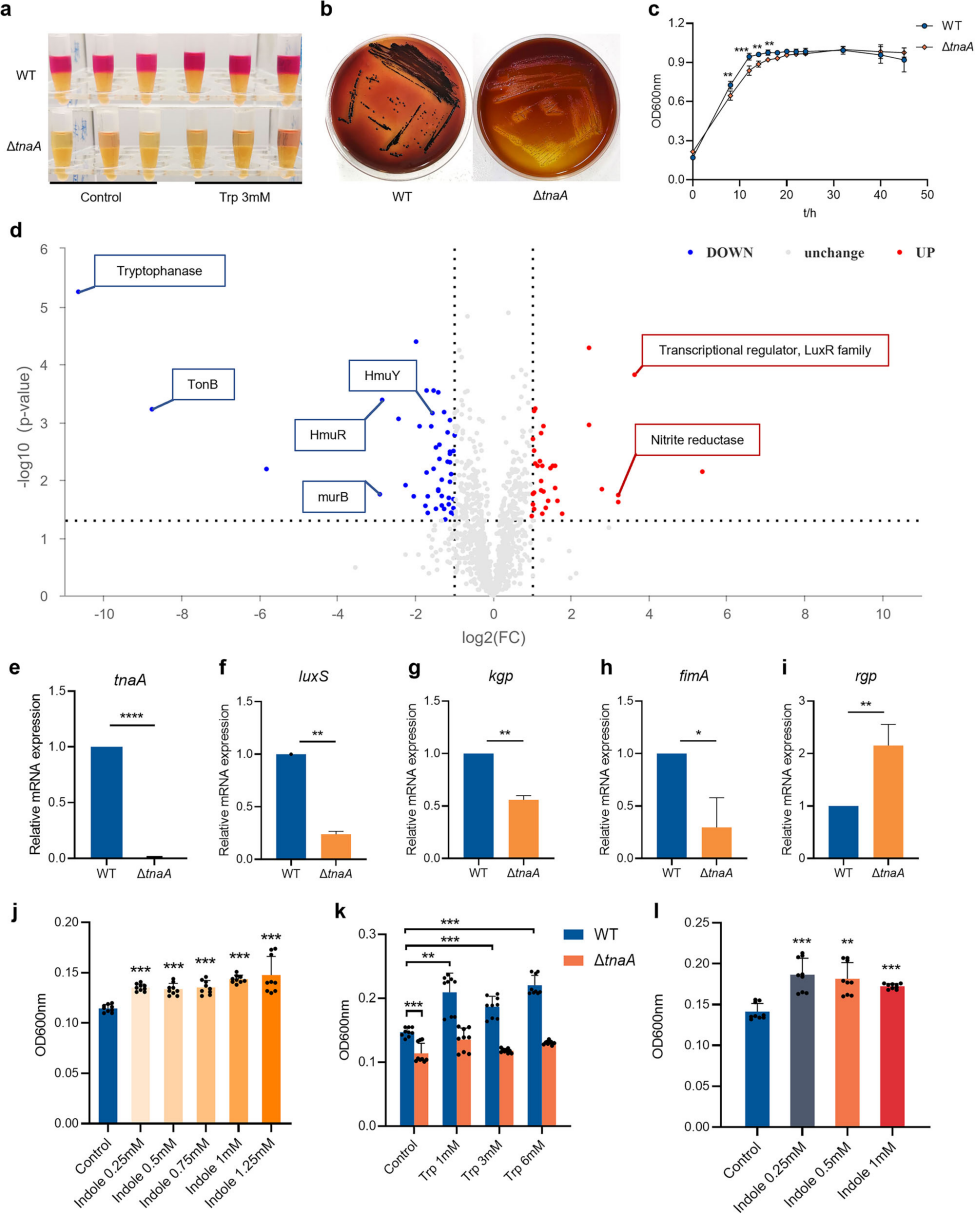
Deficiency of the *tnaA* gene impaired *P. gingivalis* growth, virulence factor expression and biofilm formation. **a** Indole assay of the 24 h-culture of WT and Δ*tnaA* supplemented with tryptophan (3 mM). The 24 h-culture of *P. gingivalis* grown in blank medium was used as control. **b** Colony formation of WT and Δ*tnaA* on the blood agar plates (7 days). **c** Growth curve of WT and Δ*tnaA*. **d** Differential proteins of WT and Δ*tnaA* after 24-h cultivation with tryptophan (3 mM) identified by proteomics. **e**–**i** qRT-PCR measurement of the *tnaA* and *P. gingivalis* virulence factors including *luxS* (**f**), *kgp* (**g**), *fimA* (**h**) and *rgp* (**i**) expression of WT and Δ*tnaA*. **j** Biofilm assay of WT under 24-h indole stimulation. **k** Biofilm assay of WT and Δ*tnaA* under 24-h tryptophan stimulation. **l** Biofilm assay of Δ*tnaA* supplemented with exogenous indole for 24 h. Statistical significance was determined using Student’s *t* test and one-way ANOVA followed by Dunnett’s multiple comparisons test. *p < 0.05, **p < 0.01, ***p < 0.001.

To investigate the role of tryptophan metabolism in *P. gingivalis*, we constructed a mutant strain (Δ*tnaA*) by knocking out the tryptophanase gene in *P. gingivalis* W83. The Δ*tnaA* showed no indole production (Fig. 3a) and lacked tnaA mRNA expression compared to the wild-type (WT) strain (Fig. 3e). We then compared the proteome and metabolome of *P. gingivalis* WT and Δ*tnaA* after 24-h stimulation with 3 mM tryptophan. Proteomics analysis identified 1130 proteins, with 48 significantly downregulated and 34 significantly upregulated in Δ*tnaA* (Table S2). Tryptophanase protein expression was nearly undetectable in ΔtnaA compared to WT (FC = 5.6 × 10^−6^) (Fig. 3d and Table S2). Significant downregulation of proteins responsible for the bacterial heme uptake and transportation were observed in Δ*tnaA*, including TonB (FC = 0.002), TonB-dependent hemoglobin receptor HmuR (FC = 0.14) and hemin transporter HmuY (FC = 0.34) (Fig. 3d and Table S2), indicating disrupted hemin utilization. Additionally, peptidoglycan synthase murB expression was significantly reduced (FC = 0.13), while the transcriptional regulator of the quorum sensing receptor LuxR family was significantly upregulated (FC = 12.43) (Fig. 3d and Table S2), suggesting impaired cell wall biosynthesis and biofilm formation in Δ*tnaA*.

**Fig. 4:**
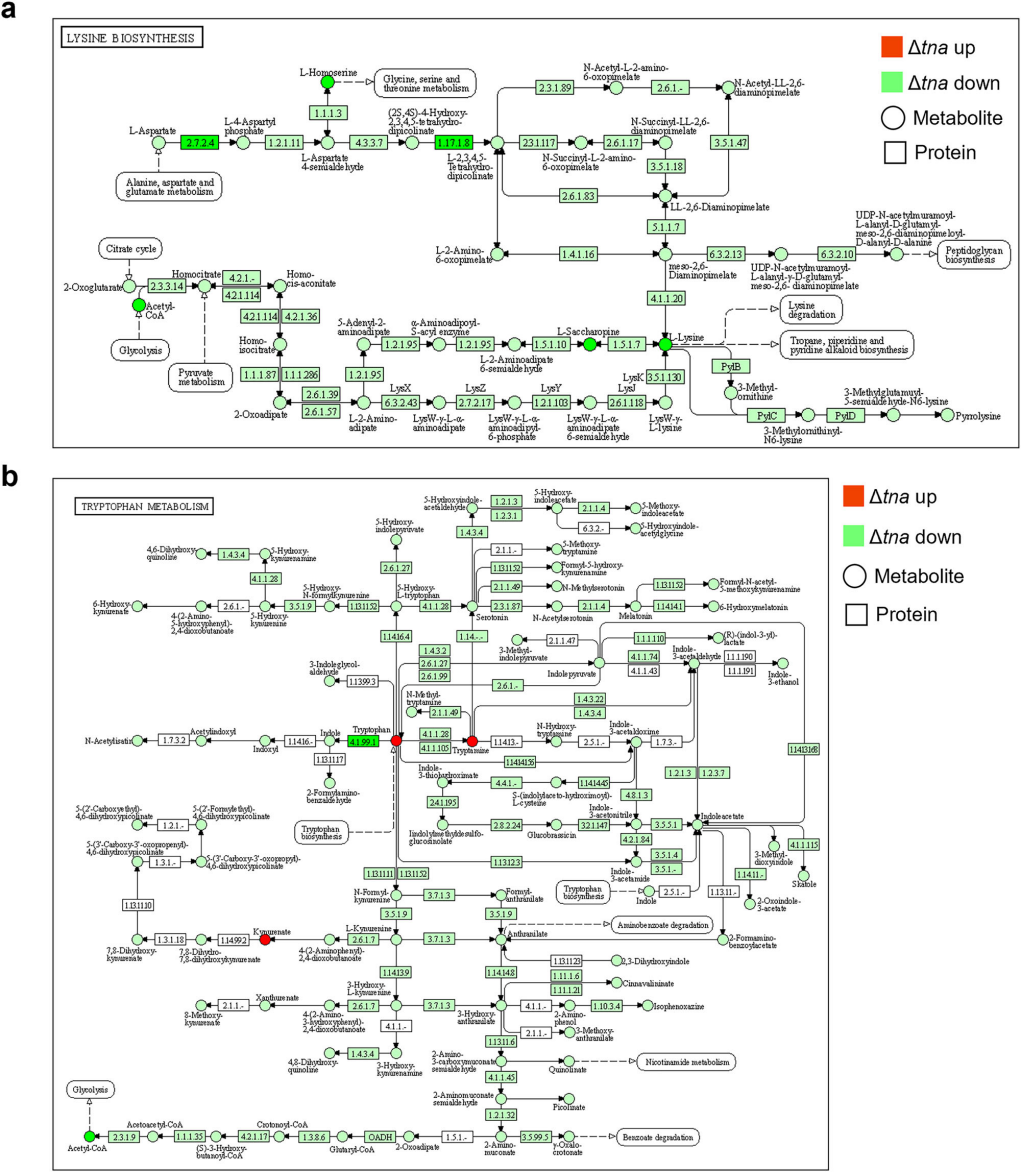
Functional analysis of *P. gingivalis* WT and Δ*tnaA* measured by combined proteomics-metabolomics after 24-h tryptophan stimulation. **a** Differential metabolites and proteins in the lysine biosynthesis pathway (KEGG MAP 00300). **b** Differential metabolites and proteins in the tryptophan metabolism pathway (KEGG MAP 00380). Red and green circles represent increased and decreased metabolites respectively. Red and green boxes represent increased and decreased proteins respectively.

Gene Ontology (GO) annotation of differentially expressed proteins in Δ*tnaA* revealed that, at the biological process (BP) level, they were primarily associated with cellular processes, metabolic processes, and signaling pathways. At the cellular component (CC) level, these proteins were mainly linked to the cell membrane. At the molecular function (MF) level, differential proteins were mainly enriched in the catalytic activity, binding and transporter functional activities of Δ*tnaA* (Supplementary Fig. 1a). Combined metabolomics-proteomics analysis revealed downregulation of pathways such as exopolysaccharide biosynthesis, lysine biosynthesis, and tryptophan metabolism in Δ*tnaA*, while pyruvate metabolism showed an upregulation trend (Supplementary Fig. 1b and Fig. 4a, b). These results showed that *tnaA* deficiency in *P. gingivalis* disrupted key proteins related to amino acid metabolism, hemin utilization, signal transduction, and biofilm formation, all crucial for its pathogenicity.

### Deficiency of the tryptophanase gene inhibited *P. gingivalis* growth, virulence expression and pro-inflammatory capacity

**Fig. 5:**
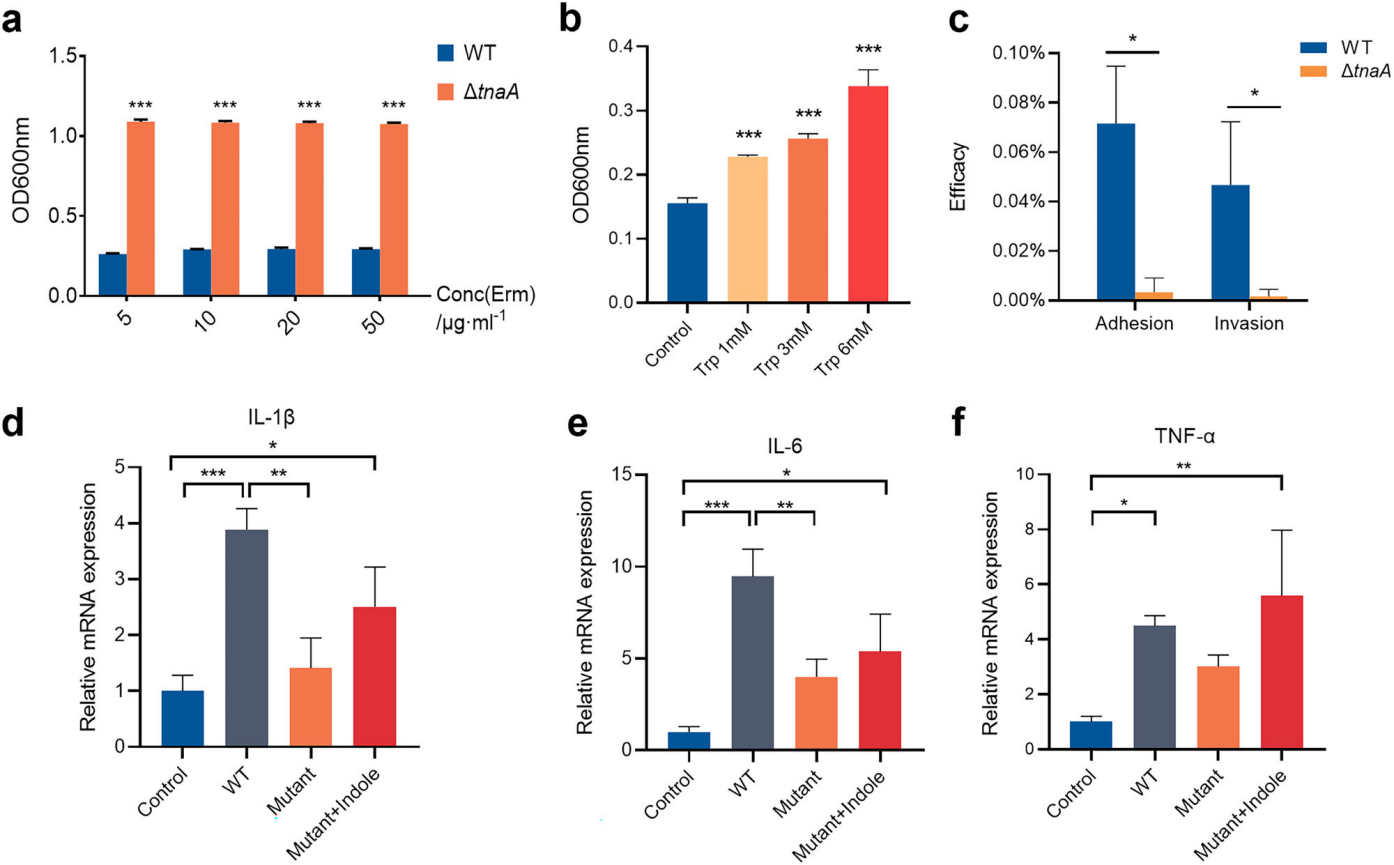
Regulation of *P. gingivalis* growth and epithelial cell invasion by *tnaA.* **a** Growth assay of WT and Δ*tnaA* in culture medium supplemented with erythromycin. **b** Biofilm assay of WT under 24-h tryptophan stimulation. **c** Adhesion and invasion assay of WT and Δ*tnaA*. The bacterial adhesion or invasion efficacy was measured by the ratio of adhered or invaded bacteria to the initial number of bacteria co-cultured with hGECs. qRT-PCR measurement of the hGECs inflammatory factor expression, including *Il-1β* (**d**), *Il-6* (**e**) and *Tnf-α* (**f**) stimulated by WT, Δ*tnaA* or Δ*tnaA* supplemented with indole (0.1 mM). Statistical significance was determined using Student’s *t* test and one-way ANOVA followed by Dunnett’s multiple comparisons test. *p < 0.05, **p < 0.01, ***p < 0.001.

To confirm the actual impact of tryptophanase deficiency on *P. gingivalis* virulence, we first looked into the bacterial growth and found that the growth assay of Δ*tnaA* showed significantly slower rates in both colony formation and planktonic growth (Fig. 3b, c). Strikingly, the black pigment production in Δ*tnaA* was severely compromised as the Δ*tnaA* colonies on blood agar plates were small and pale compared to the round black-pigmented colonies formed by the WT (Fig. 3b). The production of black pigment was recognized as a signature of the hemin utilization ability in *P. gingivalis* and other periodontal pathogens in the *Porphyromonas* and *Prevotella* genera^26^. These results confirmed that the *tnaA* deficiency significantly hindered *P. gingivalis* growth and its hemin utilization. Evaluation of the mRNA expression of *P. gingivalis* virulence factors by qRT-PCR showed significant decreases in the expression of quorum sensing signal synthase *luxS*, lysine-specific proteinase *kgp* and fimbrial component *fimA* in Δ*tnaA* (Fig. 3f–h), which were respectively responsible for bacterial quorum sensing, tissue degradation and cell adherence, while the expression level of arginine-specific proteinase *rgp* was significantly increased in Δ*tnaA* (Fig. 3i). In co-culture assays with hGECs, Δ*tnaA* showed significantly reduced cell adhesion, invasion, and pro-inflammatory ability compared to WT, with lower levels of inflammatory cytokines *Il-1β*, *Il-6* and *Tnf-α* (Fig. 5c–f).

We went further into measuring the ability of biofilm formation in Δ*tnaA*, and found that the tryptophan stimuli significantly increased the accumulation of mono-species biofilm of WT (Fig. 5b). In addition, the amount of WT biofilm could also be increased upon indole stimulation (Fig. 3j). On the other hand, the accumulation of Δ*tnaA* biofilm was significantly diminished (Fig. 3k). While the Δ*tnaA* biofilm formation could not be restored by exogenous tryptophan (Fig. 3k), supplementation of different concentration of indole was shown to restore Δ*tnaA* biofilm growth (Fig. 3l). These findings suggested that the deficiency of the tryptophan-indole metabolic pathway significantly inhibited the biofilm formation of *P. gingivalis* and indole was a crucial regulator in this process.

### The tryptophan-indole pathway of *P. gingivalis* promoted dysbiotic periodontal biofilm formation

**Fig. 6:**
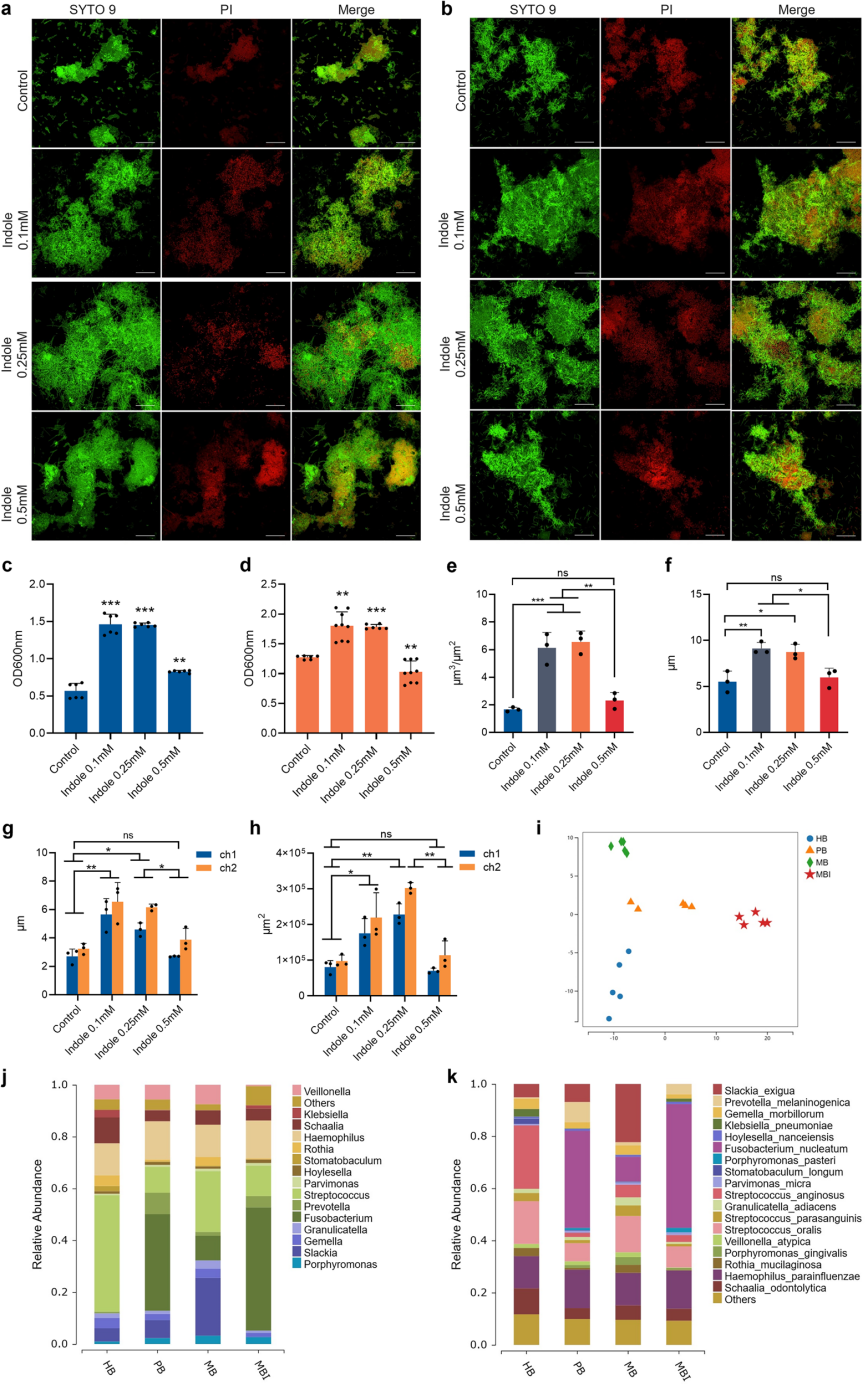
Regulatory effect of the *P. gingivalis* tryptophan-indole pathway on the polymicrobial oral biofilm. **a** Confocal microscopy image of the health-associated oral biofilm (HBiofilm). **b** Confocal microscopy image of the periodontitis-associated oral biofilm (PBiofilm). Biofilm formation assay of HBiofilm (**c**) and PBiofilm (**d**). Three-dimensional analysis of the PBiofilm, including biomass (**e**), maximum thickness (**f**), mean thickness (**g**) and surface area (**h**). **i** OPLS-DA model of oral biofilms. **j** Microbial composition of the oral biofilms on the genus level. **k** Microbial composition of the oral biofilms on the species level. The live and dead bacteria in the biofilms were separately stained by Syto9 (green) in channel 1 (ch1) and propidium iodide (red) in channel 2 (ch2) of CLSM. Merge images of the two channels were presented following the Syto9 and PI channels. PI propidium iodide. Scale bar: 20 μm. Statistical significance was determined using one-way ANOVA followed by Dunnett’s multiple comparisons test. ns no significant difference. *p < 0.05, **p < 0.01, ***p < 0.001.

**Fig. 7:**
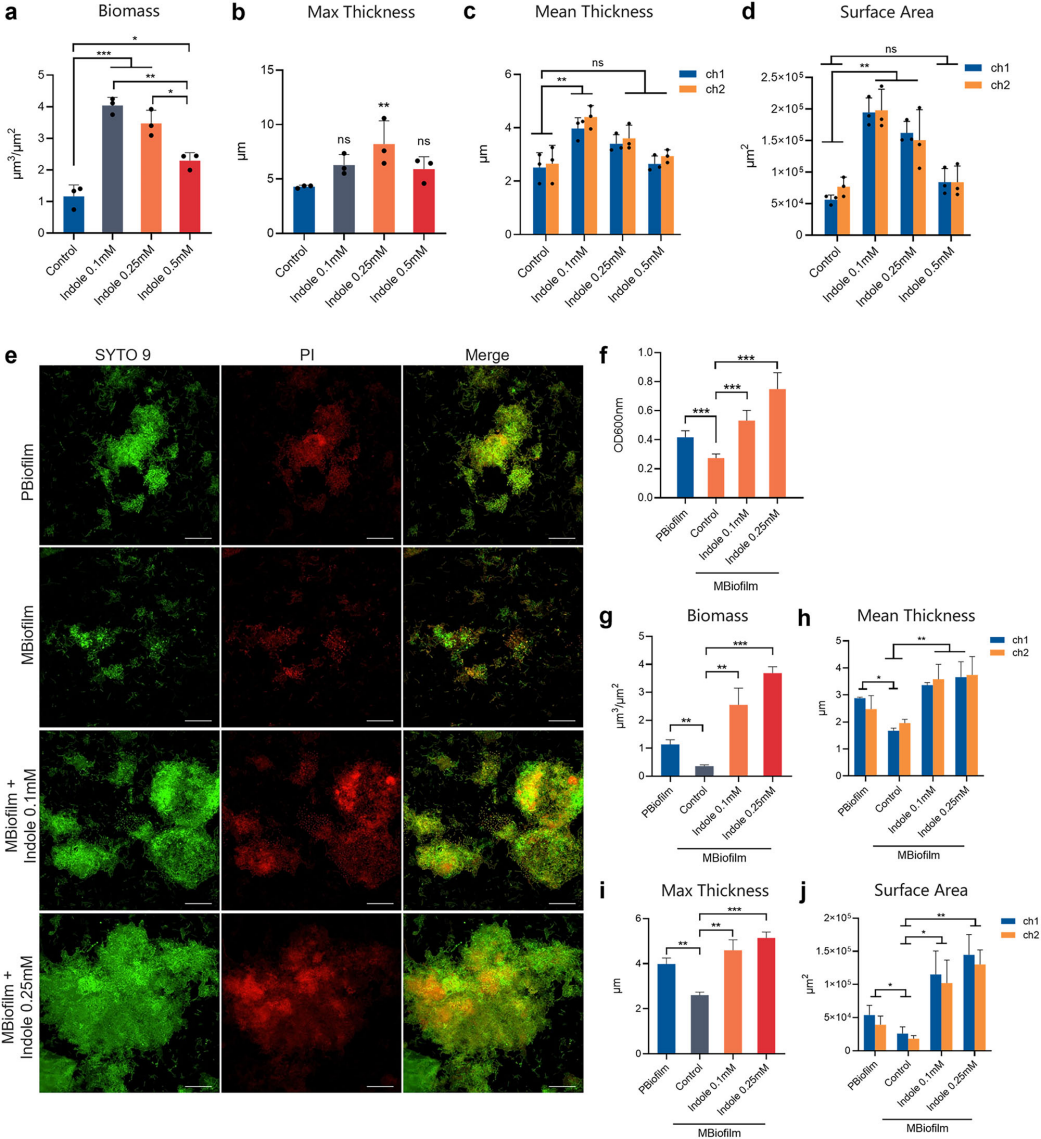
Regulatory role of the *P. gingivalis* tryptophan-indole pathway on healthy and periodontitis-associated oral biofilm. Three-dimensional analysis of the HBiofilm, including biomass (**a**), maximum thickness (**b**), mean thickness (**c**) and surface area (**d**). **e** Confocal microscopy image of the periodontitis-associated oral biofilm formed with Δ*tnaA* (MBiofilm). **f** Biofilm formation assay of MBiofilm. Three-dimensional analysis of the MBiofilm, including biomass (**g**), mean thickness (**h**), maximum thickness (**i**) and surface area (**j**). The live and dead bacteria in the biofilms were separately stained by Syto9 (green) in channel 1 (ch1) and propidium iodide (red) in channel 2 (ch2) of CLSM. Merge images of the two channels were presented following the Syto9 and PI channels. PI propidium iodide. Scale bar: 20 μm. Statistical significance was determined using Student’s *t* test and one-way ANOVA followed by Dunnett’s multiple comparisons test. ns no significant difference. *p < 0.05, **p < 0.01, ***p < 0.001.

After discovering the tryptophan-indole pathway’s regulatory role in *P. gingivalis* biofilm formation, we investigated its impact on the polymicrobial oral biofilm linked to periodontitis. We created a healthy oral biofilm (HBiofilm) using microbiota from clinical volunteers, then developed a periodontitis-associated pathogenic biofilm (PBiofilm) by inoculating it with *P. gingivalis* and *F. nucleatum*, which performed the role of a “microbial bridge” to facilitate *P. gingivalis* colonization and proliferation^27^. Confocal microscopy and crystal violet assays revealed that exogenous indole at 0.1 mM and 0.25 mM notably increased HBiofilm and PBiofilm accumulation (Fig. 6a–d). Further analysis of the biofilm structural properties showed indole significantly enhanced the biomass, maximum thickness, mean thickness, and surface area of both biofilms (Figs. 6e–h and 7a–d). In addition, the pathogenic biofilm formed by Δ*tnaA*, *F. nucleatum* and the oral microbiota (MBiofilm) was significantly inhibited compared to the PBiofilm (Fig. 7e). The MBiofilm showed significant depleted biofilm accumulation (Fig. 7f–j), while supplementation of exogenous indole restored the formation of periodontitis biofilm in three-dimensional structure (Fig. 7f–j).

16S rRNA amplicon sequencing demonstrated a clear separation between healthy and periodontitis biofilms (Fig. 6i), where in the PBiofilm, the relative abundances of oral commensals were significantly decreased, including *Streptococcus* on the genus level and *Streptococcus oralis* on the species level (Fig. 6j, k and Table S3), while the relative abundances of periodontal pathogens were significantly increased, including *Porphyromonas*, *Prevotella*, *Filifactor* and *Fusobacterium* on the genus level (Fig. 6j and Table S3). On the species level, significant increase in the relative abundance of periodontitis keystone pathogens, specifically *P*. *gingivalis*, *F. alocis* and *F*. *nucleatum* was observed in the PBiofilm compared with the HBiofilm (Fig. 6k and Table S3).

In the MBiofilm, the microbial community showed opposite compositional changes compared to the PBiofilm, with a significant increase in *Streptococcus* and *S. oralis* and a notable decrease in pathogens like *Fusobacterium* and *F. nucleatum* (Fig. 6j, k and Table S3). These compositional changes demonstrated a health-oriented shift in the periodontitis biofilm due to the tryptophan-indole pathway deficiency in *P*. *gingivalis*. In addition, indole supplementation restored the pathogenic changes by reducing *Streptococcus* and *Streptococcus oralis* while increasing the levels of the above-mentioned pathogens (Fig. 6j, k and Table S3).

Functional prediction analysis showed that the PBiofilm microbiota displayed significant upregulation trends in the amino acid transport and metabolism, lipid transport and metabolism, cell wall/membrane/envelope biogenesis, intracellular trafficking, secretion, and vesicular transport functional pathways. In contrast, the carbohydrate and nucleotide transport and metabolism pathways were upregulated in the HBiofilm (Supplementary Fig. 2a). Stimulation of the periodontitis biofilm with exogenous indole led to increased cell motility, lipid transport and metabolism, and cell wall/membrane/envelope biogenesis, while nucleotide transport and metabolism decreased, suggesting that indole may promote microbial proliferation and dispersion in the biofilm (Supplementary Fig. 2b). Therefore, the tryptophan-indole pathway in *P. gingivalis* may cause dysbiosis in the periodontal microbiota by increasing pathogen levels, decreasing commensal bacteria, and shifting the community’s metabolism from carbohydrates to amino acids.

### The tryptophan-indole pathway is crucial for *P. gingivalis* in the aggravation of periodontal bone resorption and tissue inflammation

**Fig. 8:**
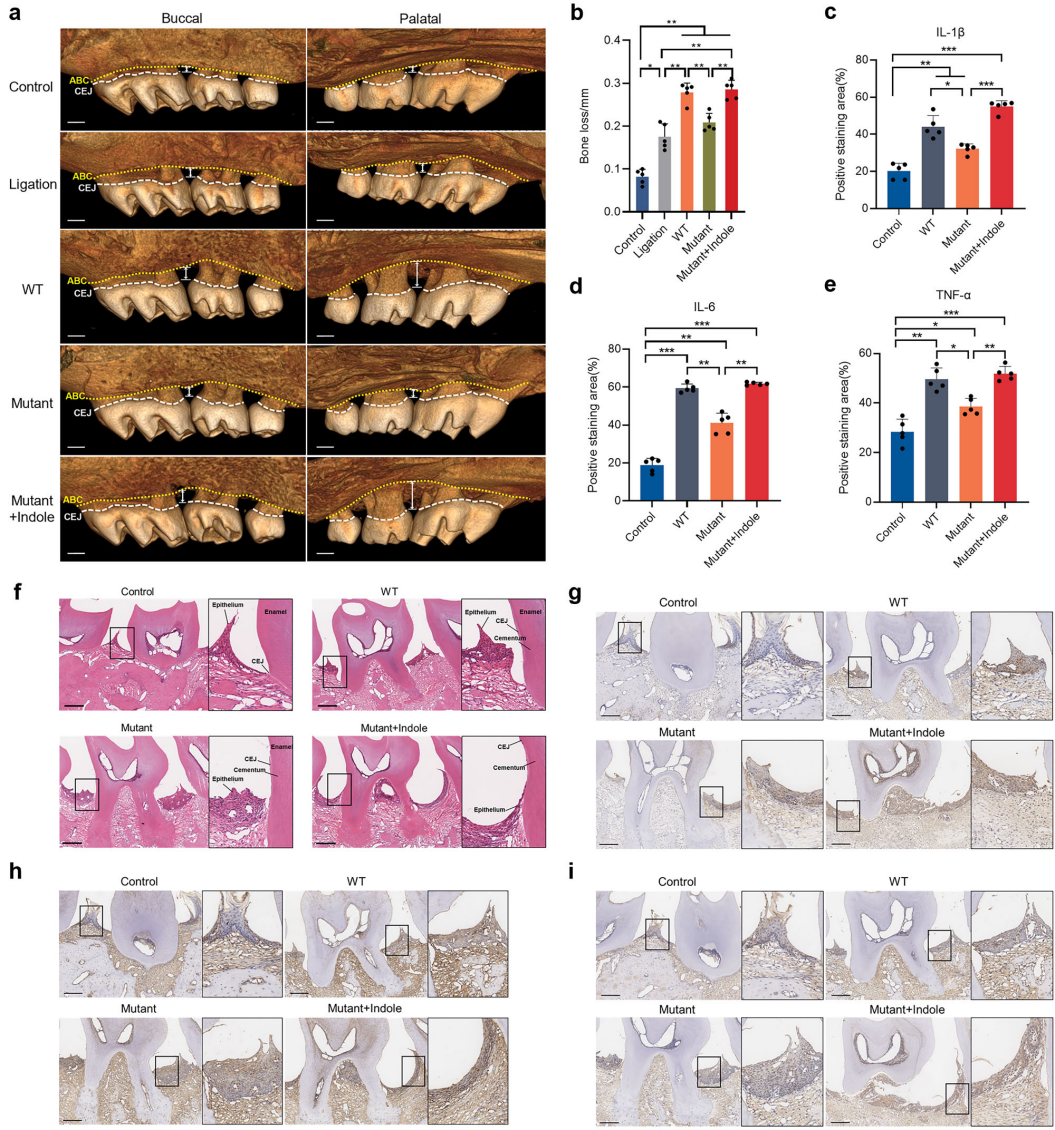
Regulatory effect of the tryptophan-indole pathway on *P. gingivalis* pathogenicity in periodontitis mice. **a** Micro-CT reconstruction of the periodontitis mice maxillae showing the distance between alveolar bone crest (yellow dotted line) and cemento-enamel junction (white dashed line). **b** Alveolar bone loss measurement of the periodontitis mice. Positive immunohistochemistry staining area measurement of the inflammatory factors, including IL-1β (**c**), IL-6 (**d**) and TNF-α (**e**) of the mice gingiva. **f** H&E staining of the mice gingiva. Immunohistochemistry staining of inflammatory factors IL-1β (**g**), IL-6 (**h**) and TNF-α (**i**). ABC alveolar bone crest, CEJ cemento-enamel junction. Scale bar: 200 μm. Statistical significance was determined using one-way ANOVA followed by Dunnett’s multiple comparisons test. *p < 0.05, **p < 0.01, ***p < 0.001.

A mouse model of periodontal disease was used to examine the role that the *P. gingivalis* tryptophan-indole pathway played in its in vivo pathogenicity. After 28 days treatment, mice in the *P. gingivalis* infection groups (WT, Mutant, and Mutant + Indole) and the ligation-only group showed significantly more alveolar bone resorption than the control group (Fig. 8a, b). The WT group had significantly higher bone resorption compared to the ligation-only group, while the Mutant group showed no significant difference from the ligation-only group (Fig. 8b). This indicates that P. gingivalis infection exacerbates alveolar bone destruction beyond the mechanical effects of periodontal ligation, a capability reduced by a deficiency in the tryptophan-indole pathway. Supplementing exogenous indole during Δ*tnaA* infection (Mutant + Indole group) significantly increased bone resorption compared to the Mutant group, matching the WT group levels (Fig. 8b), which sufficiently demonstrated that the tryptophan-indole pathway was crucial for *P. gingivalis* in the aggravation of periodontal bone destruction.

*P. gingivalis* infection also induced notable gingival inflammation, with varying degrees of gingival papilla destruction and periodontal attachment loss observed in WT, Mutant, and Mutant + Indole group mice. In contrast, the control group maintained healthy and intact epithelial attachment at the CEJ (Fig. 8f). Immunohistochemical staining revealed that WT group mice had higher levels of pro-inflammatory cytokines IL-1β, IL-6, and TNF-α in gingival tissue compared to the control group. These cytokine levels were significantly reduced in the Mutant group compared to the WT group. Consistent with the bone measurement results, indole supplementation during Δ*tnaA* infection significantly elevated IL-1β, IL-6, and TNF-α levels in the gingival tissue of the Mutant + Indole group compared to the Mutant group (Fig. 8c–e, g–i).

### Dysbiosis was induced by *P. gingivalis* through the tryptophan-indole pathway in oral and gut microbiota

**Fig. 9:**
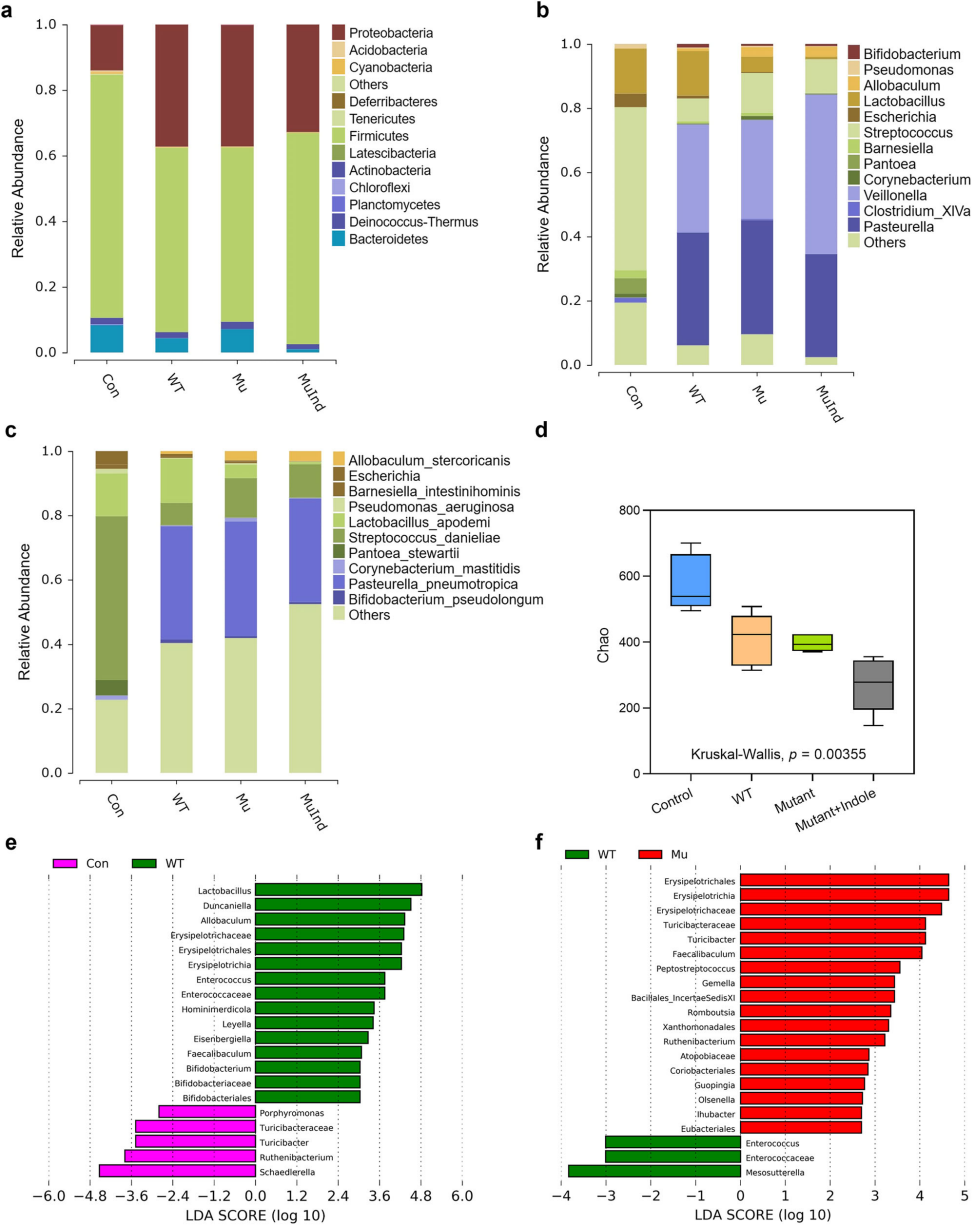
The regulatory effect of the *P. gingivalis* tryptophan-indole pathway on the oral and gut microbiota of periodontitis mice. **a** Microbial composition of the mice oral microbiota on the phylum level. **b** Microbial composition of the mice oral microbiota on the genus level. **c** Microbial composition of the mice oral microbiota on the species level. **d** Alpha diversity analysis of the periodontitis mice oral microbiota. **e** LEfSe analysis of the differential microbes in the gut microbiota of Control and WT group mice on the genus level. **f** LEfSe analysis of the differential microbes in the gut microbiota of WT and Mutant group mice on the genus level. Statistical significance was determined by the Kruskal–Wallis test.

**Fig. 10:**
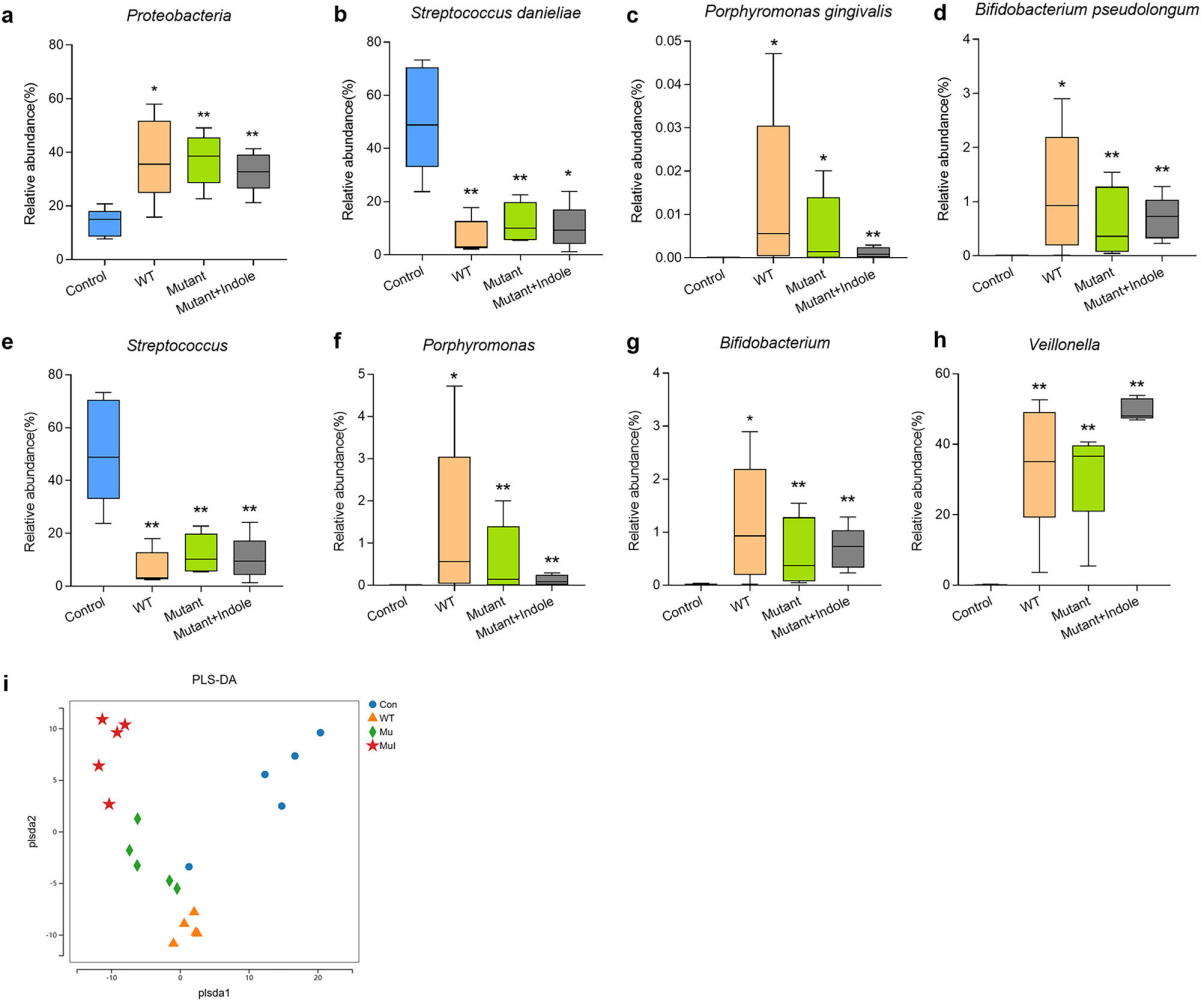
Differential microbial analysis of the oral and gut microbiota in periodontitis mice. **a** Differential oral microbe (Proteobacteria) on the phylum level. Differential oral microbes on the species level, including *Streptococcus danieliae* (**b**), *Porphyromonas gingivalis* (**c**) and *Bifidobacterium pseudolongum* (**d**). Differential oral microbes on the genus level, including *Streptococcus* (**e**), *Porphyromonas* (**f**), *Bifidobacterium* (**g**) and *Veillonella* (**h**). **i** PLS-DA model of the gut microbiota of periodontitis mice. PLS-DA partial least squares discriminant analysis. Statistical significance was determined using one-way ANOVA followed by Dunnett’s multiple comparisons test. *p < 0.05, **p < 0.01.

The dysbiotic changes of the oral microbiota in periodontitis mice were profiled by 16S rRNA sequencing, which showed that on the phylum level, Firmicutes and Proteobacteria were the core members of the oral microbiome in all mice, contributing to over 90% of the total abundance (Fig. 9a and Table S4). *P. gingivalis* infection caused a shift in microbial composition in the WT group, reducing Firmicutes from 74 to 56% and increasing Proteobacteria from 14 to 34% (Figs. 9a and 10a), which was consistent with previous studies that reported similar changing trends^28^. On the genus level, *Streptococcus*, *Lactobacillus*, *Pantoe* and *Escherichia* dominated the microbiota, making up over 70% of it, while in the WT group, *Pasteurella*, *Veillonella* and *Lactobacillus* became the main members, with an overall abundance over 82% (Fig. 9b and Table S4). On the species level, *Streptococcus danieliae* dominated the healthy oral microbiome at 51%, in contrast, its abundance dropped to 7% in the WT group (Fig. 9c and Table S4). Alpha diversity analysis also revealed a significant decline in oral microbial diversity in periodontitis mice compared to healthy controls (Fig. 9d), consistent with previous reports showing reduced diversity as periodontitis progresses^29^.

Differential analysis showed that *P. gingivalis* colonized the oral cavity in the WT, Mutant, and Mutant + Indole groups, but was absent in the control group (Fig. 10c). The abundances of the *Streptococcus* genera and the core murine commensal *S*. *danieliae* significantly decreased in oral microbiota of the WT, Mutant and Mutant + Indole groups (Fig. 10b, e), while murine periodontitis-associated anaerobes, including *Porphyromonas*, *Veillonella*, *Bifidobacterium* and its core member *Bifidobacterium pseudolongum* significantly increased (Fig. 10d, f–h). In the Mutant group, above-mentioned commensals were upregulated and periodontitis-associated anaerobes were downregulated compared to the WT group (Fig. 10b–h). With indole supplementation during Δ*tnaA* infection, *Streptococcus* and *S*. *danieliae* decreased, while *Veillonella* increased in the Mutant + Indole group (Fig. 10b, e, h). Overall, the results indicated that *P. gingivalis* induced dysbiosis in periodontitis mice via its tryptophan-indole pathway, reducing oral commensals and increasing periodontal pathogens.

We further conducted 16S rRNA sequencing on fecal samples from healthy and periodontitis mice to study the impact of *P. gingivalis* oral colonization on gut microbiota. Despite no significant signs of colon inflammation, the gut microbiota of healthy and *P. gingivalis*-infected mice showed clear differences (Fig. 10i). LEfSe analysis revealed that *Lactobacillus*, *Erysipelotrichaceae*, *Duncaniella* and *Enterococcus* were prominent in the gut microbiota of WT group mice compared to healthy controls (Fig. 9e). Notably, previous studies have linked *Erysipelotrichaceae* and *Duncaniella* with colitis in mice^30,31^, suggesting that *P. gingivalis* oral infection induces a pro-inflammatory state in the colon. In the gut microbiota of mutant mice, dominant taxa included *Turicibacter* and *Faecalibaculum* (Fig. 9f), which were typical residents reported in the gut microbiota of healthy mice^32,33^. The gut microbiota profiling results demonstrated *P. gingivalis* might promote inflammation in the colon via the tryptophan-indole pathway.

## Discussion

There is an increasing recognition of the significance of bacterial tryptophan metabolism at the host-microbiota interface in inflammatory diseases characterized by dysbiotic microbiota. In this study, we investigated the association between tryptophan metabolism and periodontal pathogens in patients with periodontitis. Subsequently, we explored the tryptophan-indole metabolic pathway of the keystone pathogen *Porphyromonas gingivalis*, validating its regulatory role in the pathogenicity of this bacterium, as well as in the oral and gut microbiota in periodontitis, for the first time.

*P. gingivalis* is known for its prominent proteolytic ability, immunogenicity, and its impact on microbial community dynamics through biofilm formation and interactions with other key pathogens^19^. Our study showed that knocking out the tryptophanase gene significantly inhibited many essential genes and proteins involved in hemin utilization and proteolysis in *P. gingivalis*. Specifically, downregulation of the *P*. *gingivalis* hemin-transporters TonB, HmuR and HmuY, and the lysine-specific proteinase was verified by proteomics and qPCR analysis. The survival and proliferation of *P*. *gingivalis* depend on these functional proteins for it is a heme-auxotroph that solely relies on exogenous heme supply. To this end, it robustly expresses gingipain proteinase Kgp to degrades tissue collagen and hemoglobins, releasing hemin into the subgingival environment^34^. Free hemin can be captured by HmuY with its histidine residue, transported into *P*. *gingivalis* by the HmuR and TonB on the cell membrane, and catabolized for iron provision in the bacterial growth^35,36^. Significant downregulation of the TonB, HmuR and HmuY proteins and *kgp* expression observed in our study strongly suggested that the deficiency of *tnaA* compromised the hemolytic activity and growth in *P*. *gingivalis*. To verify our hypothesis, we conducted bacterial growth assays and observed significant changes in *P. gingivalis* colonies on blood agar plates. The black pigment production was greatly reduced, indicating an inability to utilize hemin like the WT strain. Additionally, *P. gingivalis* exhibited a slower growth rate in BHIHK culture medium. Our findings show that tryptophan metabolism in *P. gingivalis* drives the production of gingipains and hemin-transporters, thereby modulating the proteolytic and hemolysis capability.

In this study, *P*. *gingivalis* was shown to promote its accumulation of mono-species biofilm mainly by the production of bacterial indole, as the supplementation of exogenous indole restored the once-diminished biofilm formation of Δ*tnaA*. Comparable results were also observed in the formation of polymicrobial periodontitis biofilm. These observations align with earlier studies showing the stimulative role of indole in biofilm formation of different pathogens, including *S. mutans*, *F. nucleatum*, *Vibrio cholerae*^30,31^. It is also demonstrated that *C. difficile*, although non-indole-producing, could gain a survival advantage by shifting gut microbial tryptophan metabolism to increase indole production^37^. In addition to biofilm formation, indole has also been proven to stimulate the spore formation, persister formation and drug resistance in various bacteria^31^. It functions as a bacterial signal from tryptophan metabolism by affecting different quorum sensing systems. Indole has been reported to disrupt AHL-based quorum sensing in several gram-negative bacteria, such as *Pseudomonas aeruginosa* and *Serratia marcescens*, by interfering with the stability and folding of the LuxR receptors^38–40^. Additionally, a positive correlation was found between indole and quorum sensing systems involving XIP or AI-2 molecules. Indole stimulation in *S. mutans* led to increased production of XIP and CSP signals, enhancing biofilm formation^30^. Metagenomic analysis also revealed an overall enrichment of the AI-2 synthase luxS in sludge microbiota treated with indole for 60 days^41,42^. Our study further discovered deficiency of *tnaA* and indole production in *P. gingivalis* greatly reduced *luxS* expression and biofilm formation, while LuxR family protein levels rose significantly when the inhibitory indole signal was absent. Furthermore, the AI-2/LuxS quorum sensing system is universally expressed by periodontal pathogens, such as *P*. *gingivalis*, *F. nucleatum*, *P. intermedia*, and it acts as the most important signal system in their interspecies interactions^43–45^. These results implicate that indole signals produced by keystone pathogens such as *P*. *gingivalis* can also play pivotal role by regulating their quorum sensing activities.

In a healthy subgingival environment, the dominant bacteria are mainly gram-positive cocci and rods like *Streptococcus* and *Actinomyces*, along with some gram-negatives such as *Capnocytophaga*. A shift towards a pathogenic microbiota is marked by an increase in pathogens, including the “red-complex triad” (*P*. *gingivalis*, *T. denticola* and *Tannerella forsythia*) and other key pathogens like *P. intermedia, F. alocis, and F. nucleatum*^2–4^. This study examined changes in an oral biofilm model using microbiota from healthy individuals and specific pathogens. We confirmed that periodontitis biofilm showed significant increases in *P. gingivalis, F. alocis, F. nucleatum*, and other periodontitis-related genera, while *Streptococcus oralis* and *Streptococcus* genera significantly decreased. Interestingly, these trend changes were all reversed when the *tnaA*-deficient *P*. *gingivalis*, rather than the WT was added to the biofilm, while the supplementation of exogenous indole restored the pathogenic structure of the community, inducing increased abundances of *F*. *alocis*, *F*. *nucleatum* and the *Prevotella*, *Filifactor* and *Fusobacterium* genera.

In the oral cavity of mice, however, the dominant pathogens in periodontitis progression differ from that of human. *Streptococcus danieliae* is the dominant health-related species in healthy mice, while *Enterococcus, Bifidobacterium, and Bifidobacterium pseudolongum* are the primary pathogens in periodontitis-afflicted mice^29,46^. The analysis of mice microbiota infected with *P. gingivalis* confirmed previous findings, showing increased levels of *Porphyromonas*, *Bifidobacterium* and *B*. *pseudolongum*, and decreased levels of *Streptococcus* and *S*. *danieliae*. A decline in oral microbiota alpha diversity was also was also observed as previously reported^28^. Similar with our observations of the *P*. *gingivalis*-modulated oral biofilm, the pathogenic changes in oral microbiota and tissue damage were alleviated in Δ*tnaA*-infected mice, while indole supplementation during infection restored the increasing trend of periodontitis-related microbes, as well as aggravating alveolar bone resorption and gingival inflammation. Furthermore, even without administering a chemical colitogen, changes in gut microbiota which resembled the disrupted microbial community in the state of colitis were also observed. Colitis-associated microbes including *Erysipelotrichaceae* and *Duncaniella* were characterized as the representative taxa in the gut of the WT-infected mice^47,48^, indicating that the oral colonization of *P*. *gingivalis* adversely affected the homeostasis of both oral and gut microbiota. Meanwhile, in Δ*tnaA*-infected mice, the emergence of characteristic microbes such as *Turicibacter* and *Faecalibaculum* was identified, which were reported as the dominant members in the healthy murine gut^32,33^, showing that the inhibition of *P. gingivalis* tryptophan-indole pathway could partially reverse gut microbiota. dysbiosis.

In addition to compositional changes, pathogenic alterations in the functional activities of the polymicrobial community are also key to periodontal microbiota dysbiosis. Vigorous proteolytic activity is not only the core feature of *P*. *gingivalis* metabolism, but has also been the functional signature of dysbiotic subgingival microbiota, as a result of the alterations in the dominant community members from commensals that specialize in carbohydrate metabolism to proteolysis-centered pathogens including *P*. *gingivalis*, *T. denticola*, *T. forsythia*^6,49^. Functional analysis showed significant upregulation of amino acid transport and metabolism in periodontitis biofilm, while carbohydrate transport and metabolism were inhibited. Other upregulated pathways included lipid transport, cell wall biogenesis, and vesicular transport. Indole addition further increased bacterial cell motility activity. Functional analysis revealed significant upregulation of amino acid transport and metabolism in periodontitis biofilm, while carbohydrate transport and metabolism pathway was inhibited. Other notable upregulated pathways in the pathogenic biofilm included lipid transport and metabolism, cell wall/membrane/envelope biogenesis and vesicular transport. The addition of indole further increased the predicted activity of the bacterial cell motility pathway of the community.

This study confirms the positive relationship of microbial tryptophan metabolism and disease severity in periodontitis patients, and highlights the regulatory role of the *P. gingivalis* tryptophan-indole pathway in its virulence, oral and gut microbiota dysbiosis and tissue destruction. These effects are mediated through mechanisms such as the modulation of gingipain and hemin-transporter production, disruption of quorum sensing activities, and a shift in microbiota functional activities from carbohydrate to amino acid metabolism. Consequently, this pathway emerges as a significant therapeutic target for the treatment of periodontitis. However, much work is still needed in the elucidation of its underlying molecular mechanism, including the identification of the long-debated microbial indole receptors and the regulation target of the indole signals in the quorum sensing system of periodontal pathogens. In addition, continued understanding of the role of tryptophan metabolism in host-microbe interactions in the periodontal environment is still needed in the elucidation of the mechanism of periodontitis and development of novel management strategies to address this disease.

## Methods

### Analysis of salivary tryptophan metabolites in periodontitis patients

The combined metabolomics-microbiomics data analyzed in this study were referenced from a previous study conducted by our research team^11^. Unstimulated whole saliva samples were collected from 18 Stage III and IV periodontitis patients and 13 healthy subjects, with full-mouth periodontal parameters recorded. Microbiota composition was determined via 16S rRNA sequencing, and metabolites were identified using ultra-high performance liquid chromatography (UHPLC). Spearman’s rank correlation was used to assess the relationship between microbial levels, tryptophan metabolites, and periodontal clinical parameters, with heatmaps illustrating the results. All procedures followed were in accordance with the ethical standards of the responsible committee on human experimentation (institutional and national) and with the Helsinki Declaration of 1975. Informed consent was obtained from all patients for being included in the study. Ethical approval for collection of saliva samples and gingiva from volunteers was approved by the Medical Ethics Committee of Hospital of Stomatology, Sun Yat-sen University (Issuing number: KQEC-2022-16-01).

### Bacterial strains culture and mutant construction

*P. gingivalis* W83 and *Fusobacterium nucleatum* ATCC 25586 were cultured anaerobically in a modified BHI medium (37 mg/ml BHI broth, 5 mg/ml yeast extract, 0.5 mg/ml L-cysteine hydrochloride, 5 μg/ml hemin, and 1 μg/ml vitamin K). Blood agar plates for *P. gingivalis* included these components plus 12 mg/ml agar and 5% defibrillated sheep blood.

The construction of the *P. gingivalis* mutant was performed as previously reported^25^ with several modifications. Genomic DNA from *P. gingivalis* was extracted using a TIANamp Bacteria DNA Kit (Tiangen, China). The *tnaA* gene’s upstream and downstream fragments were amplified with specific primers (Table 1). The pUC57 plasmid containing the emrAM-emrAF cassette (BIO SCI, China) was cut with KpnI, and the upstream tnaA fragment was inserted using the CloneSmarter Zero Background Topo TA DNA Cloning Kit (Epoch, USA). The plasmids were digested with BamHI, and the downstream tnaA fragments were inserted. The resulting plasmid, Tna-up-emrAM-emrAF-down-pUC57, was transformed into *Escherichia coli* DH5α and selected on ampicillin plates. The recombinant fragment, containing the upstream fragment, emrAM-emrAF cassette, and downstream fragment, was amplified using the Tna-up-F/Tna-down-R primers (Table 1). This fragment was then electroporated into *P. gingivalis* using an ECM 399 system (BTX, USA) at 2500 kV for 5 s and spread on erythromycin blood agar plates. The plates were incubated anaerobically until single clones (Δ*tnaA*) were formed. The *tnaA* gene deletion and *emrAM-emrAF* cassette insertion results were verified by polymerase chain reaction (PCR) and DNA gel electrophoresis.

**Table 1:** Primers for mutant strain construction

Primer	Sequence (5ʹ-3ʹ)
Tna-up-kpnI-F	GAATTCGAGCTCGGTACCGAATCCTATTCTCAAATATATC
Tna-up-kpnI-R	AGCGGAAGCTATCGGGAGGATTGTAACTGTGATTTTGAAG
Tna-down-BamHI-F	TTAACGGGAGGAAATAATAGAGTTAGAGAAAATACTGAAAGAG
Tna-down-BamHI-R	AGTCGACGGGCCCGGGATCCGTTGCTTCCACTGGTCTCTC
Tna-up-F	AAAGGAAGTGGAGCCGATA
Tna-up-R	GTTGCTTCCACTGGTCTCTCG
Tna-down-F	GCGGTAAGAAATCTGTATAAAG
Tna-down-R	GGTGAATGTTCCGTCTTTCAGAG
emrAM-JD-F	CAATCGAGAATATCGTCAACTGT
emrF–JD-R	TATTAGCACTTTATCAATAGTAA
Tna-ter-F	CTATCCGCAAGAGTACGCGTGAA
Tna-ter-R	CTCGATCTGCGTATCGGCAGCTT

### Indole assay

Liquid culture of *P. gingivalis* harvested at the log phase was diluted to 10^9^ CFU/ml and stimulated with 3 mM exogenous tryptophan. The bacterial culture was anaerobically incubated at 37 °C for 24 h and then harvested. The *P. gingivalis* culture was centrifuged (10,000 rpm, 2 min, 4 °C) and all supernatant was transferred into a clean tube where 500 µl of Kovac’s reagent (Solarbio, China) was added. A pink to red ring indicated positive indole production, while a yellow layer indicated a negative result.

### Untargeted metabolomics

Liquid culture of *P. gingivalis* harvested at the log phase was diluted to 10^9^ CFU/ml and stimulated with 3 mM exogenous tryptophan. The bacterial culture was harvested after 24 h of anaerobic incubation at 37 °C. Metabolomic analysis was performed using a Waters UHPLC I-Class Plus system with a QTRAP 6500 Plus mass spectrometer. A BEH C18 column (2.1 mm × 10 cm, 1.7 µm) was used for chromatography, with a mobile phase of water with 0.1% formic acid (solvent A) and acetonitrile with 30% isopropanol (solvent B). The ion source was set to 400 °C with a spray voltage of 4500 V (positive) and −4500 V (negative). The MRM method included transitions, collision energy, declustering potential, and retention time for target metabolites. Data was processed with Skyline software (v21.1) for peak picking, alignment, and metabolite identification.

### Proteomics

*P. gingivalis* was lysed and proteins were extracted using SDT buffer, then quantified with a BCA Protein Assay Kit. Protein digestion was performed with the filter-aided sample preparation (FASP) procedure as described previously^50^.

Liquid chromatography and mass spectrometry were conducted using a timsTOF Pro Mass Spectrometer (Bruker) and a Nanoelute nanoflow system (Bruker). Peptides were loaded onto a C18-reversed phase column (25 cm, 75 μm, 1.9 μm resin, Thermo Scientific) in solvent A (water with 0.1% formic acid) and separated with a linear gradient of solvent B (99.9% acetonitrile, 0.1% formic acid) at 300 nl/min. Mass spectrometry was conducted in positive ion mode with a 1.5 kV electrospray voltage and a TOF detector range of 100–1700 m/z. Data collection used the PASEF mode on the timsTOF Pro with an ion mobility coefficient (1/K0) of 0.6 to 1.6 Vs cm², including 1 MS and 10 MS/MS PASEF scans. Active exclusion was set with a 24-s release time. MaxQuant software (v1.6.14) was used for analyzing the MS data for protein identification and quantification.

### Quantitative real-time PCR (qPCR)

Bacterial RNAs were extracted with the E.Z.N.A.^®^ Bacterial RNA Kit (Omega, USA), and cell RNAs with the Super Total RNA Extraction Kit (Promega, China), per manufacturer instructions. RNAs were reverse transcribed to cDNA using HiScript III All-in-one RT SuperMix (Vazyme, China). The qPCR reaction mixture was prepared with qPCR SYBR Green Master Mix (No Rox) (Yeasen, China) and specific primers (Table 2), following manufacturer guidelines.

**Table 2:** Primers for quantitative real-time PCR

Gene	Primer sequence (5ʹ-3ʹ)
*16S rRNA*	F: AGGAACTCCGATTGCGAAGG R: TCGTTTACTGCGTGGACTACC
*tna*	F: CGAGCAGTGGATCAAGGAAGCC R: CTCTCATCGCCGAGCATCATGG
*luxS*	F: GAATGAAAGAGCCCAATCG R: GTAATCGCCTCGCATCAG
*kgp*	F: GCTTGATGCTCCGACTACTC R: GCACAGCAATCAACTTCCTAAC
*rgp*	F: CCGAGCACGAAAACCAA R: GGGGCATCGCTGACTG
*fimA*	F: TTGTTGGGACTTGCTGCTCTTG R: TTCGGCTGATTTGATGGCTTCC
*Il-1β*	F: TTCAGGCAGGCAGTATCACTC R: GAAGGTCCACGGGAAAGACAC
*Il-6*	F: CCACTTCACAAGTCGGAGGCTTA R: CCAGTTTGGTAGCATCCATCATTTC
*TNF-α*	F: ACCTCCTCTCTGCCGTCA R: AAGTAGACCTGCCCGGAC
*GAPDH*	F: AAGAAGGTGGTGAAGCAGG R: GAAGGTGGAAGAGTGGGAGT

### Biofilm assay

Saliva samples were collected from periodontally healthy volunteers in the morning between 9 a.m. to 11 a.m. The unstimulated whole saliva from each individual was pooled, and centrifuged (10,000 rpm for 30 min) to separate it into cell-free saliva (CFS) and cell-containing saliva (CCS). CFS was obtained by diluting the supernatant with sterile phosphate-buffered saline (PBS) (1:4, v/v), while CCS was prepared by adding sterile glycerol (1:1, v/v) to the precipitate. CFS served as the culture medium, and CCS was used as the biofilm inoculum for oral biofilm formation.

To form a mono-species biofilm, 1 × 10^9^ CFU/ml of *P. gingivalis* was added to sterile 24-well plates and incubated anaerobically at 37 °C for 48 h, with the medium changed every 24 h. Different concentration of tryptophan stimulant was also added to the biofilm at the starting time of the experiment. For polymicrobial oral biofilm, 15 µL CCS and 985 µL CFS were mixed per well in sterile 24-well plates and incubated anaerobically at 37 °C for 48 h in order to develop healthy oral biofilm. For the development of periodontitis-associated pathogenic biofilm, 15 µL CCS, 985 µL CFS, 10^7^ CFU/mL *F. nucleatum* and 10^7^ CFU/mL *P. gingivalis* were mixed per well in sterile 24-well plates and incubated under the same conditions. The CFS medium for polymicrobial biofilm formation was refreshed every 24 h^51^.

The taxonomic composition of polymicrobial oral biofilm was obtained by 16S rRNA sequencing. The growth of biofilms was measured by crystal violet staining and confocal laser scanning microscopy.

### Crystal violet staining

After removing the medium, biofilms were fixed with formaldehyde for 15 min, washed with Milli-Q water, and air-dried. Each well received 500 µL of 0.1% crystal violet solution and was incubated for 20 min. The plate was washed three times to eliminate excess stain. Then, 300 µL of 33% acetic acid was added to each well, and the plates were shaken for 20 min. The supernatant was collected, divided into three wells of a 96-well plate, and quantified using a Microplate Spectrophotometer (BioTek, USA) at 600 nm.

### Confocal laser scanning microscopy

For confocal laser scanning microscopy (CLSM), the biofilm was cultured in 35 mm confocal dishes for 48 h. It was then fixed with formaldehyde, washed, and stained using the LIVE/DEAD™ BacLight™ Bacterial Viability Kit (Invitrogen, USA) as per the manufacturer’s instructions. Live bacteria were stained with Syto9 (green), and dead bacteria with Propidium iodide (PI) (red). The dishes were incubated in the dark for 20 min, washed three times, mounted, and examined with a Zeiss 980 Confocal Microscope. Biofilm analysis was performed using the Comstat2 plugin (www.comstat.dk)^52^ of ImageJ software (v1.54).

### Cell culture and treatment

Primary human gingiva epithelial cells (hGECs) were isolated and cultured as previously described^53^. These cells were grown in keratinocyte growth medium at 37 °C with 5% CO_2_, and passages 2–4 were used for experiments. For bacterial stimulation, hGECs were seeded in 24-well plates and co-cultured with *P. gingivalis* (MOI = 200) for 2 h. After removing the supernatant, RNA was extracted from the cells for qPCR analysis.

### Bacteria adhesion and invasion assay

The hGECs were cultured with *P. gingivalis* harvested at log phase (MOI = 200) for 90 min in sterile 24-well plates. The unattached bacteria were removed by washing the plates with sterile PBS for three times. The hGECs were then lysed by sterile ultrapure water for 20 min and the lysates were 100-fold diluted and plated on blood agar plates to grow anaerobically for 7 days. The number of CFUs was counted to calculate the total amount of *P. gingivalis* that adhered and invaded hGECs. For the calculation of invaded *P. gingivalis*, hGECs were incubated with 300 µg/ml gentamycin and 200 µg/ml metronidazole for 1 h before being lysed to eliminate all the adhered bacteria. The hGECs were then lysed, diluted and plated as described above. The agar plates were grown anaerobically for 7 days and the number of CFUs was counted to calculate the amount of invaded *P. gingivalis*. The amount of adhered *P. gingivalis* was then acquired by subtracting the amount of invaded bacteria from all the bacteria that adhered and invaded hGECs. The results were calculated from recovered CFU values as a percentage of total bacteria in the initial inocula added each well.

### Infection and treatment of mice

A total of 25 6-week-old specific-pathogen-free (SPF) female C57BL/6 mice (Guangdong Medical Laboratory Animal Center, China) were housed in SPF conditions, exposed to 12 h cycles of light and dark, and fed with standard rodent chow and water. The mice were randomly distributed into five groups (five mice per group) : Control, Ligation, Ligation with *P. gingivalis* WT infection (WT), Ligation with ΔtnaA infection (Mutant), and Ligation with Δ*tnaA* infection plus indole supplementation (Mutant + Indole). Sample size was based on our previous study, which measured similar outcome variables that revealed significant differences^53^. All researches performed on mice were approved by the Institutional Animal Care and Use Committee (IACUC), Sun Yat-Sen University (Issuing number: SYSU-IACUC-2023-001102).

Bacterial infection and periodontal ligation were performed as described previously^54^. Non-absorbable 5-0 silk ligature was placed in the gingival sulcus around the mice second maxillary molars at both sides following intraperitoneal injection anesthesia. Oral inoculation with 1 × 10^9^ *P. gingivalis* per mouse was administered topically every other day for 28 days. After 28 days of oral infection, all mice were euthanized via overdose anesthetics. Oral swabs were collected using cotton-tipped applicators, and fecal samples were obtained from the gut lumen to assess microbial status through 16S rRNA sequencing (*n* = 5). Maxillae were resected from each mouse for subsequent micro-computed tomography (Micro-CT), histological, and immunohistochemical analyses (*n* = 5).

### Micro‑CT analysis

Mice maxillae were fixed in 4% PFA for 24 h, washed in PBS, dehydrated in 75% ethanol, and scanned using a μCT50 micro-CT (SCANCO, Switzerland) with settings of 70 kV, 200 μA, and 15 μm increments. Three-dimensional images were analyzed with RadiAnt (v2021.2). Alveolar bone loss was measured by the distance between the cemento-enamel junction (CEJ) and alveolar bone crest (ABC) at the mesial and distal points of second molars on both buccal and palatal sides.

### Histology and immunohistochemistry staining

Mice maxillae were fixed in 4% PFA for 24 h, decalcified in 0.5 M EDTA for 6 weeks, dehydrated, paraffin-embedded, and sectioned at 5 μm intervals. Sections were H&E stained. For IHC staining, slides were blocked with 5% goat serum for 10 min, then incubated overnight at 4 °C with primary antibodies, followed by 50 min with secondary antibodies. Visualization was done using a diaminobenzidine kit. Images were captured with a Leica Aperio AT2 and analyzed for positive staining area using ImageJ (v1.54).

### 16S rRNA amplicon sequencing

Bacterial genomic DNA was extracted using the QIAamp DNA Mini Kit (QIAGEN Sciences, USA). The 16S rRNA gene V3–V4 region was amplified by PCR with primers 338F (5′-GTACTCCTACGGGAGGCAGCA-3′) and 806 (R5′-GTGGACTACHVGGGTWTCTAAT-3′). PCR products were purified with Agencourt AMPure Beads and quantified using an Agilent 2100 Bioanalyzer. The amplicons were then pooled equally and sequenced on the Illumina MiSeqPE300 platform.

### Bioinformatic and statistical analysis

Data are shown as means ± SEMs from at least three independent experiments. Normality was assessed; Gaussian-distributed data were analyzed using Student’s *t* test and one-way ANOVA, while non-parametric data were analyzed using the Mann–Whitney *U* test and Kruskal–Wallis *H* test. Statistical significance was set at *p* < 0.05. Analyses and figure preparation were performed using R (v4.2.0) and Graphpad Prism (v8.0).

The 16S rRNA sequencing data were analyzed using QIIME2 (v1.16) and R packages. OTU clustering was performed with the DADA2 plugin (v1.16) in QIIME2. The resulting files were used for further analysis, with taxonomic assignment done via the RDP Classifier (v2.2) against the default database, using a 97% identity cut-off for OTUs. Alpha diversity was calculated with mothur (v1.31.2), and LEfSe analysis was conducted using an online tool (http://huttenhower.sph.harvard.edu/galaxy)^25^. The Phylogenetic Investigation of Communities by Reconstruction of Unobserved States software (PICRUSt, v2.3.0-b) was used to predict microbial community functions from marker gene sequences.

Metabolomics data were analyzed using metaX (v1.0.3). Orthogonal partial least squares discriminant analysis (OPLS-DA) assessed overall differences between treatment groups. Differential metabolites were identified using the Wilcoxon test, considering those with a false discovery rate correction (FDR)-adjusted *p* value < 0.01 and variable influence in projection (VIP) score >1 as statistically significant. Metabolite level changes between control and treatment groups were calculated using the log2 fold change (FC) ratio of normalized median signal intensities. Metabolites with FC > 1.2 were deemed increased, and those with FC < 0.67 were deemed decreased. Metabolites were categorized and annotated using the Human Metabolome Database (HMDB), and pathway enrichment was analyzed using the Kyoto Encyclopedia of Genes and Genomes (KEGG) database.

Proteomics data were analyzed using MaxQuant (1.6.14) with the uniprot_Porphyromonas_gingivalis_24320_20230124.fasta database. Proteins were quantified via LFQ in MaxQuant. Proteins with an FDR-adjusted *p* value < 0.01 and FC > 2 were deemed increased, while FC < 0.5 indicated a decrease. GO annotation and analysis were performed using InterProScan (v90.0) and Blast2GO (v1.5.1). Differentially expressed proteins were compared against the KEGG database.

## Supplementary information

Supplementary Information

## Data Availability

The data generated and analyzed during the current study are available at https://figshare.com/articles/dataset/Data_accompanying_the_paper/26388823?file=47975287.
