# Supplementary material for: Regulation of tryptophan-indole metabolic pathway in Porphyromonas gingivalis virulence and microbiota dysbiosis in periodontitis
Source: NPJ Biofilms Microbiomes. 2025 Feb 27;11:37. doi: 10.1038/s41522-025-00669-y (PMC11865485; doi:10.1038/s41522-025-00669-y)
Supplement: Supplementary file 1 — Supplementary Information [file 41522_2025_669_MOESM1_ESM.pdf]

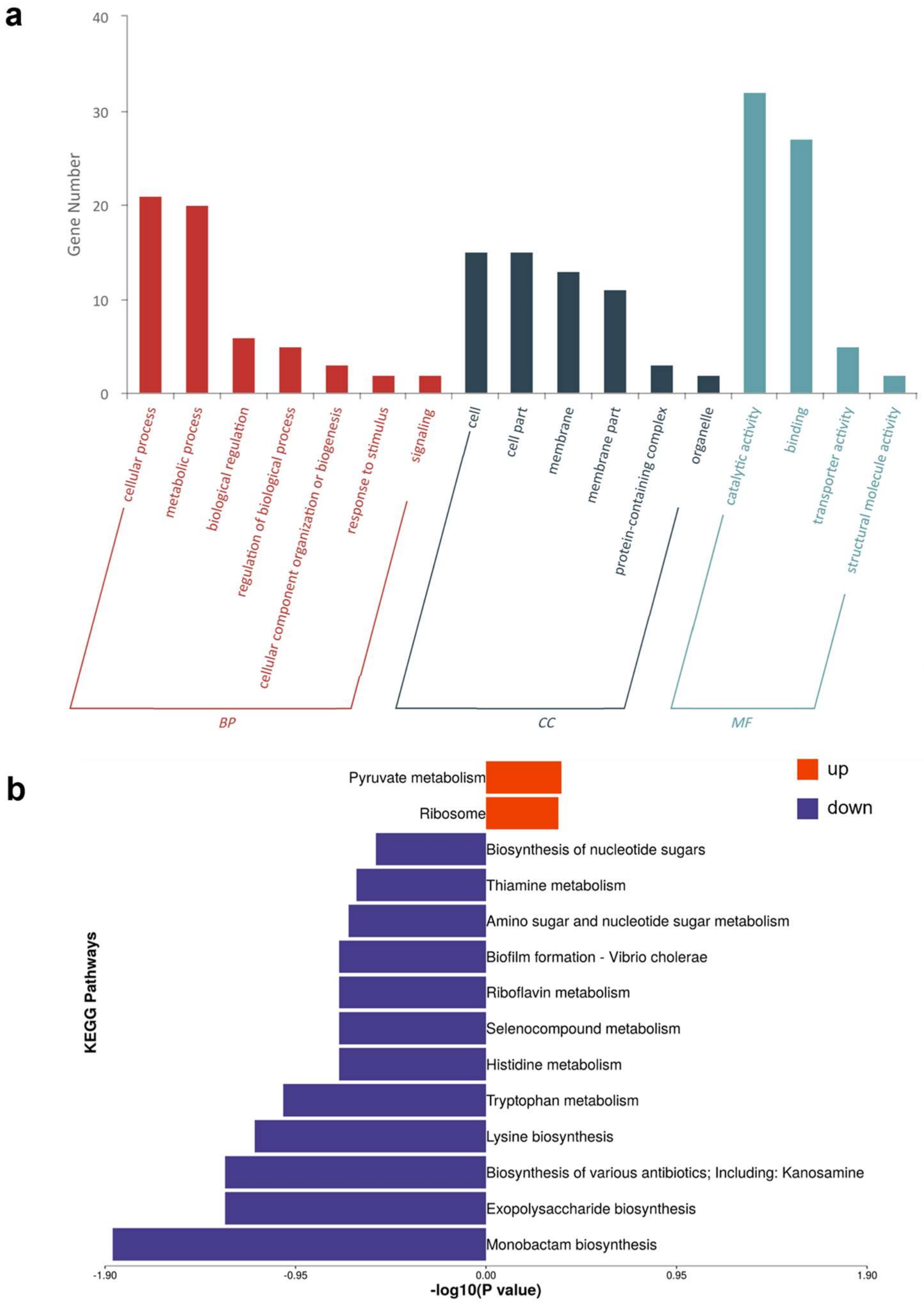

Supplementary Figure. 1 Functional analysis of *P. gingivalis* WT and Δ*tnaA* measured by combined proteomics-metabolomics after 24-hour tryptophan stimulation. **a** Gene ontology (GO) analysis of WT and Δ*tnaA*. **b** KEGG pathway enrichment analysis of Δ*tnaA* compared with WT based on the differential proteins detected. BP: Biological process; CC: Cellular component; MF: Molecular function.

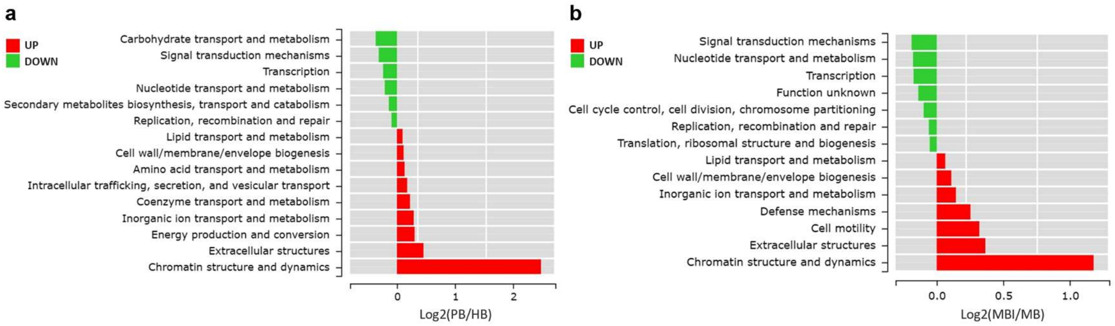

Supplementary Figure. 2 Functional prediction analysis of healthy and periodontitis-associated oral biofilm. **a** Differential function analysis between HBiofilm and PBiofilm. Red and green bars represented upregulation and downregulation trend of COG pathways in the PBiofilm compared with HBiofilm. **b** Differential function analysis between MBiofilm and MBiofilm stimulated with exogenous indole (0.25 mM) for 24 hours. Red and green bars represented upregulation and downregulation trend of COG pathways in the indole-supplemented MBiofilm compared with MBiofilm. HB: HBiofilm. PB: PBiofilm. MB: MBiofilm. MBI: indole-supplemented MBiofilm. Functional pathways presented in this figure were all statistically different between the compared groups ($p < 0.05$ and FDR < 0.05).

Supplementary Table 1 List of Differential Metabolites

Adduct ion	Metabolite	VIP	Fold Change	p-value
[M+H-H2O]+	Dl-2-methylglutamic acid	5.774982758	53.58126668	6.40576E-13
[M+H-NH3]+	5-methoxytryptamine	2.452940512	5.332848579	7.7957E-13
[M+H]+	Indole-3-acetonitrile	4.856962032	5.828235194	2.14158E-12
[M+H-C2H5N]+	4-(1-piperazinyl)-1h-indole	5.676485289	3.833025121	8.39836E-12
(M+CH3COO+2H)+	N-Carbamylglutamate	0.714820065	3.681884827	2.10398E-11
[M+H-CO2]+	Indole-3-carboxylic acid	4.995278956	4.843032792	8.49379E-11
[M+H-H2O]+	2-amino-1-phenylethanol	11.81994095	0.552865049	1.40824E-10
[M-H]-	Tryptophan	33.70807551	6.795585079	1.80966E-10
[M+H]+	Indole-3-acetamide	3.523553303	4.335647996	2.07006E-10
(M+H)+	3-Indoleacetonitrile	8.394708913	4.966491428	3.92543E-10
[M-H-CO2]-	Indoleacrylic acid	3.636334944	5.713345584	3.93736E-10
[M-H]-	Carbenoxolone	3.040731188	0.000376155	4.21309E-10
[M+HCO2]-	1,2-dioleoyl-sn-glycero-3-phosphatidylcholine	3.039495064	0.001182564	4.24786E-10
[M+H]+	DL-phenylalanine	8.114327532	0.582252711	1.68527E-09
[M+H-H2O]+	Phenylacetaldehyde	2.990112549	0.605664018	2.71279E-09
[M+H-H2O]+	Trans-cinnamaldehyde	2.391797587	5.162059849	3.80256E-09
[M-H]-	3-hydroxyoctanoic acid	3.717075181	6.452648078	6.92212E-09
[2M-H]-	DL-tryptophan	7.486590962	11.5918399	8.99741E-09
[M+H-H2O]+	3-(2-hydroxyethyl)indole	1.641039981	2.006863982	1.19545E-08
[M+H-CH6O2]+	Lappaconitine	0.780776667	3.166931924	1.63022E-08
[M+H]+	Cytosine	1.657204991	0.608401451	2.40268E-08
[M+H-C6H10O4]+	N-.alpha.-(tert-butoxycarbonyl)-l-histidine	3.102294999	0.670922052	4.46768E-08
[M+H-CH5N3]+	L-arginine, methyl ester	4.213004057	3.324262343	5.40121E-08
[M+H-H2O]+	Fenirofibrate	0.793435961	2.802446055	1.50371E-07
[M-H]-	Indole-3-carboxaldehyde	4.011298383	2.546743371	2.58006E-07
(M+H-H2O)+	Indoleacetic acid	9.015004453	3.695006438	2.90014E-07
[M+H]+	Adenosine	14.41249118	0.816141831	4.59307E-07
(M+NH4)+	Tyr-Ala	0.882975551	1.307758152	5.74587E-07
[M+H]+	Guanine	5.01049082	0.748918628	5.89111E-07
[2M+H]+	Dimefox	1.339672022	0.776945222	6.15195E-07
(M+H)+	Oxyquinoline	4.307253357	2.337498619	7.23333E-07
(M+CH3COO+2H)+	N-(omega)-Hydroxyarginine	0.774747483	1.916955706	8.9741E-07
[M+H]+	Pro-pro	2.884653687	0.83394737	1.1773E-06
[M-H]-	Indole	4.214649329	2.406746537	1.21513E-06
[M+H]+	Genistin	3.316772251	0.864779057	1.546E-06
[M+H]+	Ethyl vanillate	0.839969362	0.747405777	2.02558E-06
[2M+H]+	Alitame	1.589863546	1.320580224	3.17838E-06
[M+H]+	Soyasapogenol b base + o-hexa-hexa-dhex, o-c6h7o3(ddmp)	0.361073607	1.582865374	3.40069E-06
(M-H2O-H)-	L-Homocysteine	1.688594982	1.586822877	3.54358E-06
[M+H]+	Noscapine	0.788483805	1.207944283	3.78608E-06
[M+H]+	N2-isobutyryl-2'-deoxyguanosine	0.948333015	1.651482912	3.83787E-06

[M-H]-	L-Arabinono-1,4-lactone	0.617538785	1.875952648	5.42882E-06
(M+H)+	5-Amino-4-carbamoylimidazole	2.625291801	0.373766249	6.3396E-06
[M+H-OH]+	3-pyridinemethanol	0.733517696	0.511400236	9.21876E-06
[M+H]+	Pyroglu-tyr	0.998700459	0.792044601	1.12914E-05
[M+H]+	N6-methyladenine	1.437706881	0.885161072	1.1557E-05
[M+H]+	5-methyl-7-methoxyisoflavone	0.532590055	0.89491961	1.43014E-05
[M+2H]2+	Phe-Cys-Arg	0.65630481	0.75425433	1.6524E-05
[M+Na]+	Glipizide	0.59270957	1.107157713	2.28127E-05
[M+H]+	N-acetyl-l-aspartic acid	1.238295434	0.609837571	2.39707E-05
(M+H-H2O)+	Dopamine	1.691315329	0.755623823	2.60441E-05
[M+H]+	Linderalactone	2.363206099	2.187657077	2.80162E-05
(M+CH3CN+H)+	L-Norleucine	0.691702652	0.82029668	3.45884E-05
[M+H]+	5,10-methylene-thf	0.591621898	0.441503343	3.56119E-05
[M+H-C2H3NO]+	Bendiocarb	2.786893305	0.918960426	3.5731E-05
[M+H-NH3]+	1,5-pentanediamine	5.70943541	0.845476597	3.84451E-05
[M+H]+	DL-tyrosine	2.103024082	0.774880013	4.7786E-05
[M-H]-	Thalsimidine	1.312691387	1.765797439	4.95731E-05
[M-H]-	Taurolithocholic acid sulfate	0.824246294	2.011770033	5.05047E-05
(M+CH3CN+Na)+	DL-O-tyrosine	2.508168822	0.891373858	5.25469E-05
[2M+H]+	2'-deoxycytidine	0.489592382	0.514397421	5.4201E-05
[M+H]+	N-myristoyl-d-erythro-sphingosine	0.192376278	1.447802754	5.44719E-05
[2M+Na]+	N-acetylglucosamine	0.737228866	0.451072533	5.89967E-05
[M+H-C2H8O4]+	N-acetyl-.beta.-d-mannosamine	1.310831227	0.85124684	6.19211E-05
[M+H]+	G-guanidinobutyrate	0.821973996	1.152462011	6.35427E-05
[M+H-NH3]+	6-hydroxymelatonin	1.042209847	0.465087257	6.75551E-05
[M+H-CH3O5P]+	O-phosphotyrosine	1.701976792	0.78433419	7.45271E-05
(M-H)-	N-Acetyl-D-glucosamine	1.777878128	1.685801426	7.98582E-05
[M+Li]+	Temazepam glucuronide	0.610274394	1.328666763	8.60793E-05
(M+NH4-2H)-	Barbituric acid	0.890376146	2.475891571	9.02664E-05
[M+H]+	(2e,4e)-hexa-2,4-dienoic acid	2.020215576	0.880583464	9.89579E-05
[M+H]+	Dauricine	0.971636918	1.301742534	0.000102049
[M-H]-	3-hydroxyglutaric acid	1.562733212	1.563085316	0.00011073
[M-H]-	1,2-dimyristoyl-sn-glycero-3-phospho-l-serine	0.321229616	2.526999299	0.000111895
[M+H]+	Uridine	1.621862423	0.880604319	0.000117228
[M+H]+	L-valinamide	1.333661735	0.860408285	0.000120207
[2M+H]+	Naratriptan n-oxide	1.773331733	1.139052243	0.000123345
[M+2H]2+	His-His-Arg	2.38290219	0.927591936	0.000125638
[M-H]-	Tyrosine	4.329092253	0.725474576	0.000145239
[M-H]-	Pe(18:0/18-hepe)	0.071313824	3.23439226	0.000147627
[M+H]+	Fluocinolone acetonide	0.576730881	1.118482048	0.000149627
[M+H]+	Norvaline	2.173293893	2.543519794	0.00016616
[M+H-C5H8O3]+	5-methyl-2'-deoxycytidine	1.239399442	0.862762955	0.000166512
[M-H-H2O]-	Taurodeoxycholic acid	0.789099512	1.587182644	0.00017603
[M+H]+	4-piperidinecarboxamide	1.580205012	0.916043298	0.000178086
(M+H-H2O)+	L-Kynurenine	1.770850842	2.386830158	0.000189412
[M+H]+	Vincristine sulfate	0.597381217	1.227821465	0.000193735

[M+Na]+	Dihydrofolic acid	1.864007277	1.683490474	0.000209054
(M+Na)+	Ornithine	0.955221205	0.889212969	0.000210458
[M-H]-	2-hydroxy-3-methylbutyric acid	1.897825271	6.490442667	0.000212657
[M+H]+	Mesaconitine	1.353009876	1.09120132	0.000230079
[M+H]+	Pro-asn	0.709703807	0.893168449	0.000231773
(M+H)+	2-Hydroxyadenine	1.575306907	0.912735973	0.000259413
[M+H-H2O]+	4,8-dimethylquinolin-2-ol	0.802504929	0.812123571	0.000260196
(M+H)+	2'-O-methylcytidine	1.621348352	0.853735752	0.000260226
[M+2H]2+	Phe-Phe-Arg	3.210441848	0.826958431	0.000293872
[M-H]-	Ser-Phe	1.148493316	1.896776017	0.000316127
[M+H-H2O]+	Benzyl alcohol	0.983436074	1.400276773	0.000347146
[M+H]+	Ethosuximide	1.080131682	0.845702791	0.000381336
[M+H-H2O]+	Norbuprenorphine glucuronide	1.837070622	1.195096146	0.000401645
[2M+H]+	Mianserin n-oxide	2.313772134	1.377211565	0.000411202
(M+Na)+	Glutaraldehyde	0.86880294	0.792770864	0.000417558
[M+Na]+	Fluvastatin	3.22922949	1.09320298	0.000419483
[M-H]-	Histidine	4.588661374	0.706825151	0.00044224
[M-H]-	1-methyluric acid	0.54065756	0.514371337	0.000443792
[M+H]+	Purine	1.09501589	0.857183952	0.000446681
(M+CH3CN+H)+	alpha-Guanidinoglutaric Acid	1.40351797	0.542525489	0.000448778
[M+H]+	Glu-Val-Phe	0.361175589	0.78002894	0.000485709
[M+H]+	Malonylhexcer t18:0	0.972753722	1.509980182	0.000489212
[M+H]+	Manumycin a	0.78755579	1.264333355	0.000523336
[M+H]+	Doxycycline	1.054213296	1.402612456	0.000526621
(M+H-H2O)+	His-Val	3.920161905	0.929690258	0.000544681
[M+H]+	N-acetyl-p-fluoro-dl-phenylalanine	0.542799682	0.837814534	0.000554099
[M+H]+	Benzoylstaurosporine	0.796793425	1.153637693	0.000564011
(M+CH3COO)-	3'-O-methyladenosine	1.065474241	0.697632262	0.000570906
[M+H]+	4-[[2-[(e)-1-hydroxybut-2-enyl]-5-oxooxolan-3-yl]amino]benzoic acid	0.910268638	0.916720244	0.000637551
[M+H-C3H7ON]+	Zolpidem	7.953830413	0.939884205	0.000663886
(M-H+2Na)+	L-Alanine	2.978492144	0.927094655	0.00070397
[M+H-H2O]+	Aloesin	0.288559289	1.277582812	0.00071187
[M+H]+	Homopterocarpin	0.24548142	1.141567813	0.000720263
[M+H-2H2O]+	P-tolyl-.beta.-d-glucuronide	1.751296091	0.263274688	0.000721666
[M+Na]+	Hydrocodone	0.57763082	2.065622056	0.000735791
[M+H]+	L-glutathione, reduced	0.477990813	1.352668647	0.000751531
[M-H]-	N-acetyltryptophan	0.95304253	1.561172675	0.000764469
[M+H]+	Nintedanib	0.753171575	1.492363151	0.000772869
[M+H]+	1,2-dilauroyl-sn-glycero-3-phosphoethanolamine	0.32091909	1.384554205	0.000784111
[2M-H]-	2',6'-dihydroxy 4'-methoxydihydrochalcone	0.736987208	1.506439098	0.000821736
[M+H-NO2]+	6-quinoxalinecarbonitrile, 1,2,3,4-tetrahydro-7-nitro-2,3-dioxo-	1.296046285	2.591511103	0.000840973
[M+H]+	Dihydroergotamine	1.238971594	1.242965	0.000894232
[M+H]+	Lewis y tetrasaccharide	1.670129825	1.133203434	0.000898306

[M-H]-	Plantamajoside	0.238786669	1.378819707	0.000917509
[M+H]+	Puberanidine	0.976882134	1.25634854	0.000958532
[M+H-C5H8O3]+	Capecitabine	1.772619848	0.837357383	0.000983421
[M+H]+	S-lactoylglutathione	0.732574842	0.914307794	0.001032809
(M+H)+	Val-Ile	0.698186972	0.795244394	0.001074481
[M+Na]+	Cucurbitacin e	0.672026265	1.408505412	0.001094744
[M+H-C9H10O3]+	Scopolamine	0.789277855	0.849828913	0.001170042
[M-H+2Na]+	1-palmitoyl-2-linoleoyl-sn-glycero-3-phospho-(1'-rac-glycerol)	0.110286531	0.70529421	0.001199612
[M+H]+	S-methyl-l-cysteine	0.554013776	0.894702626	0.001228659
[M+H]+	Deoxycarnitine	1.310744893	0.961394521	0.001236236
(M+H-2H2O)+	Arg-Ser	0.993831854	0.816507162	0.001262401
[M-H]-	D-mannose 6-phosphate	0.590439561	1.464709883	0.001289812
[M+H]+	(-)-.alpha.-kainic acid	0.81717394	1.541235107	0.001293671
[M+2H]2+	Trp-Arg	1.531805469	0.923540801	0.001316534
(M-H2O-H)-	S-Allyl-L-cysteine	6.496364117	0.754065736	0.001344192
[M+Na]+	Nomilin	1.43811264	1.191892536	0.001417566
[M-H]-	D-ribose 1-phosphate	1.651046052	1.403267037	0.001434026
[M+H-H2O]+	2-hydroxybenzyl alcohol	1.662241919	0.718531871	0.001466101
[2M+H]+	D-glucosamine 6-phosphate	0.62088109	0.901469958	0.001851379
(M+CH3CN+Na)+	N-Methyltyramine	0.613811826	1.266845427	0.001858271
[M+H]+	Trigonelline	1.61444201	0.951141098	0.001894468
[M+H]+	Citrulline	5.561681918	0.899605001	0.001906756
[M+Na]+	Eleutheroside b	0.69676061	0.798111958	0.001927564
[M-H]-	Malonic acid	0.421725312	1.519224427	0.001955833
[M+H]+	Pyroglu-phe	0.981506778	1.056574542	0.001957697
[M+Na]+	2-isopropylmalic acid	1.597637904	0.927574501	0.001957943
[M-H-H2O]-	Lasalocid	0.432636536	1.455653335	0.001972908
[M+H]+	4-ohe1-1-n3ade	0.273441829	1.127369373	0.001988467
[M+H]+	Trp-Pro	0.837121886	1.788049334	0.002061585
[M+Na]+	Cyanazine	0.26552851	1.166488678	0.002081181
(M+CH3COO)-	Ribulose 5-phosphate	0.288558971	1.36716553	0.002121752
[M-H]-	Phenylalanine	8.396853867	0.768147	0.002181941
[M+H]+	Gly-Pro-Arg	1.135635375	1.159116048	0.002202067
[M+H]+	Pyroglu-pro	1.785965623	0.914505818	0.002272387
[M+H-H2O]+	Glycyl-l-4-hydroxyproline	1.847411135	0.928702447	0.002306432
[M+H]+	Indole-3-acetaldehyde	1.400669873	1.614994162	0.002347648
[M+Na]+	Norcocaine	0.489875956	0.823521326	0.002373119
[2M-H]-	N-acetylglucosaminylasparagine	0.077172619	1.346273122	0.002386087
[M+H]+	Pyrazophos	0.387867774	0.916797131	0.002447425
[M+H-C5H8O3]+	Pentostatin	0.985075034	0.937171779	0.002470844
[M+H-C10H10O2]+	Bisdemethoxycurcumin	3.12039745	2.713254003	0.002500945
[M-H]-	8-hydroxyquinoline-2-carbaldehyde	1.499225436	2.436868505	0.002545166
[M+H-H2O]+	Diacetoxyscirpenol	0.610319136	1.141984101	0.002634099
[M+Na]+	Aal toxin tb	0.832297376	1.205554962	0.002639433
[M+Na]+	Acetylpodocarpic acid anhydride	0.641678319	1.767250179	0.002680166
(M+H)+	Sissotrin	0.833658411	0.906771276	0.002768155

[M+Na]+	Entacapone 3-.beta.-d-glucuronide	0.844208225	1.619484526	0.002782992
(M+H)+	Tyr-Glu	1.36950805	3.769293432	0.002820679
[2M+H]+	2-ethylhexyl diphenyl phosphate	0.868732257	1.103427203	0.002825218
[M-H]-	Esculetin	1.201828835	1.501955625	0.002906353
[M+H]+	N(6)-oh-me-adenosine	2.392911656	4.812287559	0.003049534
[M+Cl]-	Jujuboside b	0.357414348	0.784017155	0.003065637
(M-H+2Na)+	(+)-Methamphetamine	0.541853881	0.847919594	0.003073349
[M+H]+	Decoyinine	1.23276512	0.794612183	0.003182908
[M+H]+	Zafirlukast	0.507091795	0.849132435	0.003222358
[M-H]-	Guanosine diphosphate mannose	0.016068121	1.09097547	0.003245993
[M+H]+	Rhaponticin	0.319021695	1.187633344	0.003290215
[M+H]+	Methylguanidine	1.285925839	1.042278761	0.003357356
[M+Na]+	Dulcoside a	0.836157369	1.173302571	0.003390194
[M+H-H2O]+	Amikacin	2.244727829	1.144113972	0.003399791
[M+H]+	Hymenamide c	0.709531509	1.351696533	0.003539854
[M+H+2i]+	Halofantrine	0.649489225	1.16578965	0.003672903
[M+H-H2O]+	4-hydroxybenzyl alcohol	1.002079621	0.698009524	0.003811859
[M+H]+	Calpain inhibitor ii	0.518282376	0.881385674	0.003848539
[M+H-CH4O3]+	Anisatin	0.779218188	1.36087373	0.004043927
[M+H-2H2O]+	Cyasterone	3.452295468	1.253612462	0.004180874
[M+HCO2]-	1,2-di-(9z-tetradecenoyl)-sn-glycero-3-phosphocholine	0.121370444	1.621376998	0.004220626
[M+H-H2O]+	2-acetylamino-3-mercapto-3-methylbutyric acid	0.535642335	0.886581441	0.004260282
[M-H]-	Rifapentine	0.414201104	0.789846234	0.004307647
[M+H-H2O]+	1,2-dihexadecanoyl-sn-glycerol	1.091830417	1.358903664	0.004319979
[M-H]-	N-acetyl-d-mannosamine	1.022064171	1.453324857	0.004362946
(M+CH3COO)-	D-Mannose	1.830951736	1.696608487	0.004371619
[M+H]+	2-dimethylamino-6-hydroxypurine	0.698418897	0.899184855	0.004450257
[M-H]-	Asn-Phe	0.526181945	1.425483529	0.004497393
[M+H-C6H13O]+	2-heptyl-4-hydroxyquinoline n-oxide	4.510459529	9.193898126	0.004597486
[2M-H+2i]-	Tecloftalam	0.314762938	2.476540954	0.004600521
[M+H]+	Arg-Asp-Arg	1.288796937	1.321953973	0.004677441
[M-H]-	Udp-n-acetylglucosamine	0.188515779	1.555063823	0.004718536
[M+H]+	Syringic acid	0.651550812	0.906448261	0.004843166
[M+Na]+	Fumonisin b1	0.668639262	1.218477416	0.004895526
[M+H]+	Phe-arg	0.389015563	0.699926974	0.005025743
[M+Na]+	Ikarugamycin	1.524742634	1.213575769	0.005084788
[M+H]+	Methylprednisolone succinate	0.669743857	1.262334993	0.005138382
(M+CH3COO)-	Dihydrouracil	0.994134212	1.293762922	0.005141054
[M+H-C6H10O5]+	Narirutin	0.916124428	0.895077933	0.005177922
[M+H]+	Pyroglu-Lys-Arg	0.983531495	1.419604886	0.005246645
[M-H]-	3-hydroxy-7,8,2',3'-tetramethoxyflavone	0.557734315	1.318716229	0.005341161
[M+Na]+	Scopolin	0.48125151	1.253290191	0.005372776
(M+H)+	.beta.-Homoproline	5.212418315	3.686220356	0.005431837
[M+H]+	Thiazolidine-4-carboxylic acid	0.731592328	0.908218615	0.00545441

[M+Na]+	Obacunone	0.627987342	1.040113207	0.005485561
[M+H]+	2',4,4',6'-tetramethoxychalcone	0.508140072	0.922166313	0.005691978
[M-H]-	Dl-a-hydroxybutyric acid	1.055253208	1.274498107	0.005777752
[M-H]-	8-gingerol	0.37218512	1.54214089	0.005875098
[M+H]+	Monalide	0.425435905	0.895173295	0.005980459
[M+H]+	Tyr-Leu	0.138199869	1.064208606	0.006033288
[M-H]-	Asp-Val	0.882824392	1.518734428	0.006151636
[M+H-C2H4O2]+	N-.alpha.-acetyl-l-ornithine	2.228695462	0.945369536	0.00619999
[M+Na]+	Dofetilide	0.458467749	1.085657789	0.006212989
[M+H]+	(-)-1,2:5,6-di-o-cyclohexylidene-l-inositol	0.27492406	0.801666123	0.006339798
[M+H]+	Pro-cys	3.594691924	2.568461891	0.006383043
[M+Cl]-	K-strophanthoside	0.371642799	1.360444711	0.006569709
[M+H]+	Orientin	0.491981649	1.259171089	0.006575217
[M+H]+	1-methyladenosine	2.867187507	0.910856797	0.007022681
[M-H-CO2]-	1h-indole-3-carboxylic acid, 1-pentyl-	1.073869862	1.313249302	0.007136766
[M+H]+	2-mohsa [dmed-fahfa]	0.642448358	1.389586094	0.007239537
[M+Na]+	Beta-octylglucoside	0.305203626	1.134137759	0.007384545
[M-H]-	Xanthine	5.081984378	1.241110146	0.007390567
[M+H]+	2-methylbutyryl-l-carnitine	0.66736052	0.948669111	0.007467102
[M+H-C5H8O4]+	Cytidine	1.317948687	0.962299259	0.007525642
[M+H]+	Ecgonine	0.430681131	0.864879156	0.007600074
[M+H-H2O]+	Indole-3-carbinol	11.93337665	4.007269164	0.007792668
[M+H]+	Guan-fu base y	0.857969445	1.167223175	0.00787197
[M+H]+	Leu-Asp-Lys	0.481866831	1.173643958	0.007877449
[M+Na]+	3'-fucosyllactose	0.881655799	1.745623323	0.007973887
[M-H]-	Hexsose + hexsose-deoxyhexose + c20h32	0.05823516	1.818860209	0.008124723
[M-H]-	5-hydroxyindole	0.922951117	1.346104906	0.008162334
[M+H-H2O]+	4',6'-dimethoxy-2'-hydroxyacetophenone	0.36118612	0.900222049	0.008325637
(M+NH4)+	Uridine 5'-diphosphate (UDP)	0.296219818	0.562727711	0.008354548
[M+H]+	Ser-Thr	0.503882704	1.337423619	0.008366192
(M+H-H2O)+	His-Ile	6.335020901	0.9535011	0.008443135
[2M-H]-	2-cis-4-trans-abscisic acid	0.722311093	1.343012043	0.008509749
[M+H]+	N-stearoylsphinganine	1.000116723	1.333436349	0.008594881
[M+H]+	Ser-Trp	0.368352965	1.115157275	0.008858056
[M-H]-	2',6'-dihydroxy-4'-methoxyacetophenone	0.405892217	0.711040741	0.008938085
[M+H]+	1-(3-pyridyl)-1-butanone-4-carboxylic acid	1.467636068	0.712683222	0.009046301
[M-H]-	Dipyridamole mono-o-.beta.-d-glucuronide	1.091011921	1.225952008	0.009201195
[M+H]+	3-methylcatechol	1.205474752	0.880580658	0.009297454
[M+H]+	Tyr-Gly	0.387433119	0.858910801	0.009519547
[M+K]+	Glycocholic acid	0.517786465	1.428045613	0.009543027
[M+H]+	Pyroglu-Gln-Lys	0.995812755	1.238373961	0.009543934

[M+H-H2O]+	Latrunculin a	1.912917735	1.787740274	0.009591921
[M+H-H2O]+	Cytotrienin a	0.404287109	1.163400767	0.009954571
[M+H]+	PC(16:0/16:0)	2.141744917	205.7704812	0.010136385
[M+H]+	5,2'-O-dimethylcytidine	0.441002028	1.17238593	0.010212595
[M+H]+	Leu-Val-Lys	2.099779262	0.002117064	0.010221361
[M+H]+	Eprosartan	0.427124706	1.371061166	0.010294964
[M+K]+	NCGC00380845-01	0.267403691	1.362566171	0.010402868
[M+Na]+	Cucurbitacin i	1.115012067	0.894026655	0.010426733
[2M-H]-	Tetradecylphosphonate	2.12634658	1678.60939	0.010511121
[M+H]+	Hoiamide b	0.546618933	1.368424193	0.010528736
[M+H-H2O]+	Montelukast	0.339654563	1.175297357	0.010564337
[M-H-CH2O2]-	Indolelactic acid	9.020094766	4.895645448	0.010837351
[M+H]+	Nitromide	0.35283564	0.922657322	0.010917256
[M+H]+	Adenine	5.162233208	0.94746218	0.010925555
[M-H]-	Hexosyl lpe 18:3	1.846068027	1.27635793	0.010960189
[M+K]+	2-hexadecanoylthio-1-ethylphosphorylcholine	0.507951588	0.88138838	0.011017794
[M+H-H2O]+	Baccatin iii	0.511589956	1.044180085	0.011164758
[M+H]+	Cyanophos	0.781508587	0.665575252	0.011316206
[M+H]+	Nelfinavir	2.883582062	1.131175702	0.011350066
M-	m-Chlorohippuric acid	1.284760722	0.760910715	0.011396045
[M+K]+	Nocardamine	0.873863625	1.132422764	0.011467713
(M-2H+3Na)+	N-Desmethylselegiline	0.505835032	0.775240835	0.011503938
[M+H]+	Withaferin a	0.223588052	1.080432846	0.011550299
[M-H]-	Kynurenic acid	0.8190215	1.476719702	0.011758541
[M+H]+	Telmisartan	0.491160209	1.687431584	0.011780802
[M+H]+	Pyridoxine	4.175608482	0.935474517	0.012107648
[M+Na]+	Thapsigargin	0.879688068	1.16134817	0.012170231
[M-H]-	Voacamine	0.12323549	1.554388816	0.012185963
[M+H]+	Loperamide	0.316844525	1.368858464	0.012307329
[M+H]+	Bipleiophylline	0.465321324	1.318455182	0.01277725
[M-H]-	Pentaerythritol tetrakis(3,5-di-tert-butyl-4-hydroxyhydrocinnamate)	0.459452816	0.641305752	0.012876241
(M+H)+	Guanosine	0.989233736	0.861707786	0.012955926
[M+Na]+	Taxol c	0.570675388	1.382665771	0.012999634
[M+H]+	Phytosphingosine	0.138059973	0.575596697	0.013039307
[M+H+2i]+	Jasplakinolide	1.3122249	1.168405723	0.013226893
[M+H]+	Docetaxel	0.356254243	1.115561043	0.013427923
[M-H]-	Gly-Phe	0.613911728	1.294038151	0.01342897
[M+H]+	Gln-Tyr-Arg	0.425124726	1.312869285	0.01359641
[M+H]+	Ethambutol	0.403421115	1.211443755	0.0136919
(M+H)+	Adenosine monophosphate (AMP)	0.723454084	0.544593493	0.013784023
[M+H]+	Diethyl phthalate	0.193748117	1.312996651	0.013944926
[M+H]+	Vandetanib	0.274399623	1.242175752	0.013954092
[M+H-C4H10O]+	Tulobuterol	0.186672179	0.827972785	0.014013122
[M+H]+	Daidzin	1.015834309	0.939610904	0.014267359
[M+H]+	Palmitoyl sphingomyelin	0.183212163	1.526033431	0.014281356

[M+H]+	Atrazine-desisopropyl-2-hydroxy	0.703510082	2.261237134	0.014752676
[M-H]-	Cytidine 3'-monophosphate	0.286328126	1.290323087	0.015581585
[M+H]+	Aspartame	0.518467063	1.318763362	0.015585732
[M+NH4]+	Kasugamycin	1.114576264	0.916730738	0.015717222
[M-H]-	Pg(18:1/8,9-eet)	0.05830592	2.060156898	0.015766688
[M+H]+	Hydroxyflutamide	0.1662672	1.318167389	0.015924101
[M+H]+	Pro-val	0.569665562	1.114586013	0.016103831
[M+H]+	His-Glu	1.916009552	0.957926364	0.016231706
[M+H]+	Solanidine base -2h + 1o, o-hex-dhex-dhex	0.522337277	1.352520021	0.016694454
[M+H]+	Trp-Met(o)	0.538091066	0.970969006	0.016750561
[M+Na]+	1,2-ditetradecanoyl-sn-glycero-3-phosphocholine	0.173333118	1.388772602	0.016896539
[M-H]-	Soyasapogenol b base + o-hexa-pen	0.088323086	1.391906867	0.01706898
[M+H]+	2'-o-methyladenosine	7.631453092	0.946352504	0.01730192
[M-H]-	4-ketopimelic acid	0.925440078	1.197659268	0.017522172
[M+H-C2H4O2]+	Hypaconitine	1.046686795	0.918356063	0.017884238
[M-H]-	N-3,4-tridhydroxybenzamide	0.633835693	1.295675098	0.017911104
[M-H]-	Adenosine 3',5'-diphosphate (pap)	0.173012295	0.684199771	0.018455898
[M+H-H2O]+	Folinic acid	0.473489048	1.053829001	0.018638094
[M+H-H2O]+	3-methyl-2-buten-1-ol	0.753889553	0.855984517	0.018681211
[2M-H]-	Loxistatin acid	0.07497298	1.326197857	0.018825018
[M+H-CH5O5P]+	D-erythro-imidazolylglycerol phosphate	3.103768708	0.729843868	0.01887576
(M-H)-	D-Glucosamine 1-phosphate (Glucosamine-1P)	0.306250981	1.874135671	0.019064054
[M+H]+	Diethyl azelate	0.264144877	0.924560794	0.019067874
[M+CH3COOH-H]-	Deoxycholic acid methyl ester	0.521903488	0.810962211	0.01913617
[M-H]-	Pe(16:1/8-hete)	0.047079503	2.086002348	0.019174082
[M-H]-	Aleuritic acid	0.236598283	0.581155806	0.019193072
[M+H-C4H6N2O2]+	Dflnha	8.317884604	0.917183618	0.01953559
[2M+H]+	5-aminooxindole	0.321074318	1.048709679	0.019601007
[M-H]-	Ile-Val	0.25692701	1.258077899	0.019733987
[M+2H]2+	Phe-Cys-Lys	0.395161085	0.813741832	0.019877927
(M-H)-	Salinomycin	0.236008609	1.301892554	0.020771186
[M-H]-	11-ketofusidic acid	0.180054456	0.653483429	0.020784779
[M+H]+	Irbesartan	0.715331312	1.200866637	0.020800269
[M+H]+	Lys-Ala-Lys	0.378135262	1.287151804	0.02100173
(M+H-H2O)+	Phe-Gln	4.54814639	0.869747597	0.02172867
[M+H]+	Isobutyric acid	0.795490957	0.898550432	0.022247628
[M+H-H2O]+	Glycerol 1-myristate	0.284291665	1.225170267	0.022284
[M+Na]+	Prosulfuron	0.253834702	0.892658216	0.022413075
[M+Na]+	1-palmitoyl-2-glutaryl phosphatidylcholine	0.432418616	1.181354893	0.022501523
(M+H)+	Anthranilic acid (Vitamin L1)	0.671777725	0.678023483	0.022588527
[M+H]+	Pyridaben	2.110477494	0.827496428	0.022664153
[M+H]+	Purpurin	0.441878419	1.343044631	0.02282087

[M+H]+	5-(2-hydroxyethyl)-4-methylthiazole	1.377211376	0.93441309	0.022876885
[M+H]+	Isopentenyladenosine	2.154976118	0.900893469	0.0230891
(M+H)+	1-Palmitoyl-2-oleoyl-sn-glycero-3-phosphoethanolamine	0.363551392	1.409510893	0.023290223
[M-H]-	Glycochenodeoxycholate	0.184635457	1.22099278	0.023319986
(M+H)+	Leu-Tyr	1.433185865	3.180865073	0.024669485
[M+H]+	4-hydroxyquinoline	7.1033931	3.240900159	0.024727943
[M-H-C6H9N3O2]-	Trifloxysulfuron	0.193420653	1.336862064	0.024803392
[M+H]+	Ferrichrome	0.931380899	1.26202342	0.024838802
[M-H]-	hydrocortisone 21-acetate	0.666731817	1.540494715	0.024953745
[M-H]-	Phe-met-arg-phe-amide	0.540989235	0.738220123	0.02498464
[M+H]+	Pyroglu-val	2.096698339	0.957894423	0.025029598
[2M+H]+	16-oxocafestol	0.280278571	0.900680291	0.025320462
[M-H]-	His-Lys	4.244341452	0.828924812	0.025414723
[M+H]+	Permethrin	0.384913407	1.492231825	0.025878992
[M+H-C3H6]+	Stachydrine	0.591281706	0.783010875	0.025957245
[M+H]+	Thioperamide	0.379389983	1.141343579	0.026029805
[M+H]+	Biphalin	0.348082647	0.939761188	0.026165433
[M+H]+	Trp-Ser-Lys	0.530210076	1.153291371	0.026745184
[M-H]-	2-despiperidyl-2-(5-carboxypentylamine)repaglinide	0.347174204	1.174696638	0.026947802
[M+Na]+	Rifaximin (xifaxan)	0.592280698	0.725610157	0.02727159
(M+H-H2O)+	DL-Indole-3-lactic acid	8.419137989	5.620153686	0.027547459
(M+H)+	L-Tryptophan	3.52808127	1.520638616	0.027708003
(M+H)+	L-Threonine	2.36628206	0.330009657	0.028059457
[M+H]+	3-aminoquinoline	13.95694787	11.11906146	0.028558994
[M+OH]-	Platencin	0.394148082	1.287325196	0.029393594
[M+H]+	N-lauroyl-d-erythro-sphinganine	0.34880597	0.793828004	0.029601911
[M-H]-	Xylitol	0.676806861	1.283314775	0.029833239
[M-H-H2O]-	Mevalonic acid 5-pyrophosphate	0.305885478	0.763787663	0.030950748
[M+H]+	Ile-Asp-Lys	0.504390714	1.095445337	0.031707968
[M-H]-	Deoxyguanosine	1.054228868	0.793130409	0.031884041
[M+H]+	Deoxypumiloside	0.649598476	0.621347012	0.032627119
[M+H]+	Morphine-3-glucuronide	4.118721961	0.684274296	0.033152249
[M-H]-	Hematoporphyrin	0.170487808	1.115325965	0.033259706
[M+2H]2+	Pro-Glu-Arg	0.834075226	0.8700926	0.033789776
[M+K]+	Convallatoxin	0.550998818	1.295809139	0.033816999
[M+H]+	Asp-Glu	0.699642366	0.943438262	0.033854122
[M+H]+	Vindesine	0.151482653	0.92516301	0.033860648
[M+H]+	Trifluoroperazine	0.648295707	1.271999444	0.033985049
[M+H]+	Phe-thr	0.344690948	0.876976175	0.03439417
[M-H]-	N-isovaleroylglycine	0.152340513	1.405424013	0.034616584
[M+H]+	Glu-Leu	1.342671697	0.485837312	0.034876386
[M+H]+	Pentacarboxylporphyrin i	0.219499178	1.051879569	0.034961233
[M-H]-	4-hydroxyphenylacetic acid	2.811617532	1.120934358	0.035162711
[M+H]+	N-acetyl-d-lactosamine	0.240246852	1.30572464	0.035169728
[M-H]-	Neomangiferin	0.588491371	2.178404113	0.036074118

[M-H+2Na]+	Androstan-3-ol-17-one 3-glucuronide	3.43734437	1.18996144	0.036528962
[M+H-C6H10]+	Acetohexamide	0.816090062	0.906440652	0.036574008
(M+H-2H2O)+	2-Amino-2-methyl-1,3-propanediol	2.830950559	1.086124225	0.036993475
[M+H]+	N,n-dimethylguanosine	0.603042491	0.935208643	0.037254594
[M-H]-	Linoleic acid	0.86899843	0.709129992	0.037267202
[M+H]+	2(1h)-pyridinone	0.79392328	0.96225079	0.037331985
[M-H]-	N-formylmethionine	0.749032612	1.172264554	0.037337476
[2M+H]+	Indolmycin	1.360738644	1.240219716	0.037429314
(M+H-2H2O)+	Matairesinol	0.470258094	0.897449694	0.037623449
[M+H]+	Reserpic acid	0.32160237	1.148345175	0.037939662
[M-H]-	5-methyltetrahydrofolic acid	0.661019444	1.281884237	0.038210852
[M+H]+	Glu-Gly-Glu	0.65492548	0.924383497	0.038346559
[M+H]+	Pyroglu-Glu-Lys	0.570916448	1.13593268	0.038891552
[M+2H]2+	Pro-Ile-Lys	0.595398781	1.05192045	0.039191551
[M+H]+	Met-Glu-Lys	0.652325869	2.091048997	0.039199891
[M+H]+	Ser-Glu-Lys	0.449794602	1.226710471	0.039612033
[M-H]-	Tauroursodeoxycholic acid	0.343801382	1.327768709	0.039697491
(M+H)+	Lys-Ile	0.436856536	0.907264501	0.03977388
[M+H]+	1,2-dipentadecanoyl-sn-glycero-3-phosphoethanolamine	2.783328854	1.211043826	0.039809502
[M-H]-	Glu-Pro	1.043870351	0.303812121	0.039840965
[M+H]+	Hexylglutathione	0.30010951	1.104902226	0.039884929
[M-H]-	(+)-fluprostenol	0.483163027	1.214840758	0.039929751
(M+NH4)+	D-4-Hydroxyphenylglycine	0.676921137	0.599986848	0.039976922
[M+Na]+	Geldanamycin, 17-demethoxy-17-[[2-(dimethylamino)ethyl]amino]-	0.459122582	1.156311327	0.040686523
[M+H]+	N-palmitoylglycine	0.271085433	0.658250999	0.040787834
[M+H]+	Asn-Ala-Arg	0.811771391	1.081880706	0.040828808
[M+H]+	1-deoxynojirimycin	0.702864945	0.881062662	0.040983304
[M+H]+	Glu-Trp-Lys	0.509401166	1.139440487	0.041144557
[M+HCO2]-	.beta.-acetyldigoxin	0.24859008	1.293270368	0.041240505
[M-H]-	Gambogenic acid	0.446632868	1.633725425	0.041326825
[M+H-H3SO2N]+	Zonisamide	4.127177409	5.596373705	0.041466672
[M+H]+	Pyrrol	0.74723257	0.641583217	0.041481786
[M+H]+	Albiflorin	0.671509515	0.777566562	0.042751161
[M+H]+	Glu-Glu-Lys	0.472006242	1.321663226	0.043295326
[M+H]+	2-aminoadipic acid	0.571804476	1.157292247	0.043487072
(M+H-2H2O)+	gamma-L-Glutamyl-L-phenylalanine	4.063756569	0.842384306	0.04379584
[M-H]-	Pentadecanoic acid	2.318381757	1.490569324	0.04447289
[M+H]+	Neomycin	0.609322699	1.640345332	0.044539485
[M+H]+	Syrosingopine	0.527260103	0.963609551	0.04455854
[M-H]-	L-citrulline	5.532853961	0.85704407	0.044803739
[M+H-H2O]+	2-palmitoyl-rac-glycerol	1.175469483	1.216679636	0.045574431
[M+H-H2O]+	(+)-2,5-epi-goniothalesdiol	1.289124143	0.865005751	0.046324049
[M+H-NH3]+	1,2-diamino-2-methylpropane	2.509006572	0.93821512	0.046353027
[M+H]+	Gambogic acid	0.722891953	1.099013395	0.046421815

[M+H]+	Creatinine	8.962673814	1.062743614	0.046879179
[M-H]-	Glucose	0.68567927	1.104249152	0.047421667
[M+H]+	Diethofencarb	0.339245345	1.156046868	0.047556489
[M-H-H2O]-	4-hydroxy-l-glutamic acid	0.746236485	0.898267605	0.048518124
[M+H]+	Benzoic acid eugenyl ester	0.332615201	0.936390763	0.04866466
[M+H-C5H10]+	Pendimethalin	0.441917508	0.924833291	0.0488643
[M-H]-	Licoricidin	0.325205037	1.236594577	0.048894876
[M+H]+	Dl-.beta.-homoleucine	1.485174555	0.177071216	0.049206711
(M+Na-2H)-	Phosphocreatine	0.575628088	0.757126118	0.04965751

Supplementary Table 2 List of Differential Proteins and Metabolites

Protein	Protein Name	Mutant/WT	p value
Q7MTI7	Por_Secre_tail domain-containing protein	41.05570809	0.007181191
U2LI89	Transcriptional regulator, LuxR family	12.43311796	0.00014871
U2JJY1	Peptidase, M28 family	9.254853606	0.02403936
U2LJZ5	[2Fe-2S]-binding domain protein	9.242522992	0.018423354
T2N9L4	Putative DNA-binding protein	6.963422667	0.014422984
U2JQQ4	Rubrerythrin	5.511137002	0.001145613
T2N907	GDSL-like protein	5.489098953	5.33636E-05
A0A212FV32	SusC/RagA family TonB-linked outer membrane protein	3.391380202	0.038906352
U2KAE5	Outer membrane lipoprotein carrier protein LolA	3.169906851	0.02286507
Q7MUS0	Ferredoxin	3.015599764	0.013820004
U2K9K3	Sporulation and cell division repeat protein	2.989962179	0.005646621
Q7MW50	Membrane protein, putative	2.90309134	0.005836551
U2K971	50S ribosomal protein L14	2.787080112	0.006152321
A0A0K2J4X1	Por_Secre_tail domain-containing protein	2.682114022	0.022591687
U2L9M8	Peptidase Do	2.576544944	0.030502265
U2LDA4	DUF3078 domain-containing protein	2.428634787	0.001167578
U2K9N1	30S ribosomal protein S14	2.424245528	0.015961016
Q7MW91	Lactoylglutathione lyase, putative	2.414696172	0.00565644
U2K4N5	Bacterial transferase hexapeptide repeat protein	2.414626913	0.038183038
A0A212G043	Methylase_S domain-containing protein	2.343206945	0.015486309
Q7MVM2	Por_Secre_tail domain-containing protein	2.341943542	0.001520422
U2LIC0	DUF4494 domain-containing protein	2.331846229	0.010517049
T2N959	PF06803 family protein	2.314396616	0.004711373
U2K727	Chloride transporter, ClC family	2.20313887	0.005593782
Q7MW10	AbiTii domain-containing protein	2.105857268	0.005261276
A0A0E2M7Q2	KTSC domain-containing protein	2.104431151	0.000590197
T2NAP0	SH3 domain protein	2.081254797	0.003112026
Q7MV04	Chorismate synthase	2.079736532	0.032343087
U2K4C4	Transcriptional regulator, AsnC family	2.069553233	0.01683317
U2LKG5	Transcriptional regulatory protein RprY	2.054772176	0.000643424
U2K0E9	ABC transporter, ATP-binding protein	2.042872037	0.017142885
Q7MWJ7	DUF3421 domain-containing protein	2.040459031	0.026533488
A0A0K2J3R8	OMP_b-brl_3 domain-containing protein	2.026557391	0.001970966
T2NB88	Large-conductance mechanosensitive channel	2.003687684	0.042383539
T2NDT2	Ferrous iron transport protein B	0.499133094	0.001687399
Q7MT06	Putative pre-16S rRNA nuclease	0.497299577	0.021536928
U2KXF6	Elongation factor Ts	0.49584872	0.038873808
Q7MWU8	Polysaccharide biosynthesis-related protein	0.492199171	0.030862593
U2K3Q7	Acyl carrier protein	0.492134163	0.00317171
T2NE60	TonB-dependent receptor	0.475128733	0.007955918
Q7MUQ7	ATPase, putative	0.472650442	0.036940841
A0A1R4DWG7	Two-component sensor histidine kinase	0.464483767	0.004963971
A0A0K2J737	OMP_b-brl domain-containing protein	0.463270213	0.000927753
T2NBF2	Glycosyltransferase-like protein, family 2	0.462955164	0.003546188

T2NF68	DNA helicase	0.461731105	0.003266366
Q7MWC2	META domain-containing protein	0.46078711	0.010804914
U2JJS2	RNA methyltransferase, RsmD family	0.455051642	0.026500086
Q7M7B7	HTH hxlR-type domain-containing protein	0.450544441	0.020496147
Q9XBW2	Immunoreactive 47 kDa antigen PG97	0.446011988	0.00151086
Q7MUR5	6,7-dimethyl-8-ribityllumazine synthase	0.440975297	0.004856801
A0A829KMC8	Imidazolonepropionase	0.427329539	0.048421795
T2NCU8	Dihydrodipicolinate reductase	0.417291336	0.031821387
U2K3W7	Aspartokinase	0.414225763	0.000674433
U2K8F7	Nucleotidyltransferase	0.401778831	0.027740925
Q7MUI3	Conserved domain protein	0.399732417	0.019028324
U2KXI9	Fibronectin type-III domain-containing protein	0.396377125	0.00994139
Q7MUL1	DNA-binding protein, histone-like family	0.387168987	0.004341782
A0A1R4AD73	Peptidyl-prolyl cis-trans isomerase	0.38714012	0.002467941
A0A212G726	1-deoxy-D-xylulose-5-phosphate synthase	0.379555857	0.000306708
T2N8V3	Methyltransferase domain protein	0.378845028	0.01548081
A0A2D2ND24	CRISPR-associated helicase/endonuclease Cas3	0.37586301	0.014690982
T2N7Q1	Selenide, water dikinase	0.360571498	0.031658038
U2K599	Trk system potassium uptake protein TrkA	0.357587941	0.002753705
U2LHJ5	NADPH-dependent 7-cyano-7-deazaguanine reductase	0.343008747	0.006440965
T2NAD0	ATP-dependent Clp protease ATP-binding subunit ClpX	0.342635515	0.000285623
U2JKI8	HmuY family protein	0.340803673	0.000700906
Q9R4B5	24 kDa hemin-binding cell envelope protein (Fragment)	0.334988031	0.001188354
A0A829KGR5	Peptidase M16 inactive domain protein	0.311253325	0.037291095
U2KWU3	DUF1573 domain-containing protein	0.3109871	0.019252096
T2ND73	PF08984 domain protein	0.304327364	0.00747367
B2RM55	CRISPR-associated protein Cmr3	0.303057456	0.000284923
T2N9S1	TraB family protein	0.297483105	0.028141587
A0A212G719	Secretion protein	0.271515372	0.001184126
Q7MV98	Membrane protein, putative	0.253924306	4.07716E-05
U2JYU4	Peptidyl-prolyl cis-trans isomerase	0.243412162	0.019270268
Q7MT16	Transcriptional regulator, putative	0.211241119	0.012368962
Q7MUG8	CobN/magnesium chelatase family protein	0.185590487	0.000881413
U2K3E1	TonB-dependent hemin utilization receptor HmuR	0.138925148	0.0004146
U2LP79	UDP-N-acetylenolpyruvoylglucosamine reductase	0.134068222	0.017745626
U2K253	Transcriptional regulator, TetR family	0.017587388	0.006497026
A0A212G4K7	Biopolymer transporter TonB	0.002323058	0.000601104
T2NER6	Beta-eliminating lyase	0.000624062	5.59545E-06

Adduct ion	Metabolite	VIP	Fold change	p-value
[M-H]-	Oxypurinol	18.692007	4.724592969	2.1887E-15
[M-H]-	Pseudouridine	32.77936	0.008970852	6.3023E-14
[M+H]+	Gln-Glu-Lys	2.6210854	7.380034612	9.8013E-14
[M-H]-	Xanthosine	1.6206112	5.687734781	1.2782E-13
[2M+Na]+	Formononetin	3.3147335	0.068578808	1.3768E-13
[M-H]-	Uracil	20.11658	17.81184926	8.7219E-13
[M+H]+	14-benzoylaconine	9.9526595	5.70259187	1.0066E-12
[M+Na]+	7-(triethylsilyl)-10-deacetylbaccatin iii	3.0676221	17.54690969	1.0635E-12

[M-H]-	Medicagenic acid base + o-hexa	2.3213527	3.160018103	1.1114E-12
[2M+H]+	Cytidine	11.052278	0.003955745	1.2957E-12
[M+Na]+	Microcolin c	7.3982063	12.48063376	1.7927E-12
[M+H]+	Xanthine	5.3344112	5.101679517	4.899E-12
[M+H]+	Veratridine	5.6356922	10.0357744	6.0332E-12
[M+H]+	Cytosine	9.7957982	0.009606703	6.6991E-12
[M-H]-	DL-tyrosine	9.0750595	2.290345435	7.4908E-12
[M+H-C5H8O3]+	Deoxyguanosine	20.173256	0.004648164	1.0969E-11
[M-H]-	Leukotriene d4	2.9991682	6.662929393	1.2103E-11
[M+H]+	Bursin	2.2678017	3.490520664	1.2525E-11
[M+H]+	Hypoxanthine	20.740698	3.249076375	1.6973E-11
[2M-H]-	Pipemidic acid	1.6183882	3.705667156	1.9554E-11
[M+H-NH3]+	5-aminoimidazole-4-carboxamide	2.1028673	3.413783413	2.7443E-11
[2M-H]-	Benzoylnorecgonine	1.8825808	3.577089928	3.3028E-11
[M+H]+	Acetic acid, phenyl ester	7.8795963	0.134489263	4.0498E-11
[M+Na]+	1,2-bis(o-octanoyl)-sn-glycerylphophorylcholine	1.129596	3.153113057	5.1561E-11
[M+Na]+	Ouabain	1.6639854	2.912374369	7.8444E-11
(M-H)-	Thymidine	7.2762861	0.13169583	8.4469E-11
[M+H]+	Phe-Trp-Arg	4.1921565	4.82286288	8.7728E-11
[M-H]-	Inosine	7.2955587	0.130320611	9.3741E-11
[M+H]+	Gamma-l-glutamyl-l-glutamic acid	2.5449721	2.068420547	1.6638E-10
[M+H-H2O]+	Olmesartan medoxomil	2.2547553	2.381784284	2.0313E-10
[M+H]+	Pro-Glu	3.9814717	2.343096318	2.8743E-10
[M+H]+	Guanine	9.7482365	0.291393289	3.1268E-10
[M-H]-	His-Lys	18.410115	0.00286165	3.31E-10
[M+H]+	Vinblastine	1.3515958	3.264283662	3.9677E-10
[M+K]+	Apicidin	2.2837978	3.950603801	5.2804E-10
[M+H]+	Val-leu-pro-val-pro	9.7720247	19.99414721	5.2827E-10
[M+H-C9H15NO3S]+	Captopril disulfide	3.1591981	0.463231603	5.4096E-10
(M+CH3COO)-	Hydroxyacetone	3.6125291	0.43639288	5.5877E-10
[M+Cl]-	Lnfp v	1.1611135	0.116217507	7.541E-10
[M+Na]+	3,7-o-diacetyl-5,14-o-dibenzoylmyrsinol	1.93427	2.254375789	7.7358E-10
[M+H]+	N-palmitoyl-d-sphingosine	0.4880815	5.344921071	8.692E-10
[M+Cl]-	K-strophanthoside	1.88506	0.040084779	9.5072E-10
[M+H]+	Uridine	2.9495299	0.101455034	9.8393E-10
[M+H]+	Cerivastatin	0.9346867	3.525449999	9.9031E-10
[M+H]+	Ser-Asp-Lys	1.8521432	1.604747135	1.0001E-09
(2M+Na)+	Phenacetine	3.4298796	2.152748492	1.0906E-09
(M+H)+	5-Methylcytosine	1.6065344	0.170133673	1.1181E-09
[M+H]+	Thr-Glu-Lys	1.6667311	3.086492156	1.2406E-09
[M+H+K]2+	G2f	0.9138655	0.536858783	1.6452E-09
[M+H]+	5-methylcytidine	1.7004242	0.111510662	2.4772E-09
[M-H]-	Leucine	22.843771	0.567765411	2.5338E-09
[M+H-H2O]+	Montelukast	0.9148798	2.080816703	3.2008E-09
[M+H]+	Erythromycin	54.372118	24901.54403	3.8019E-09
[M+Na]+	Euphorbiasteroid	1.5506806	5.279734687	3.8311E-09

[M+Na]+	3-(n-maleimidopropionyl)biocytin	1.7111166	3.460484741	3.9774E-09
[M-H-C6H5NO2]-	Nicotinate d-ribonucleotide	1.8973702	0.144611472	4.4087E-09
[M+Na]+	Turkesterone	1.0004019	4.499174991	4.5249E-09
[M+H]+	2'-deoxycytidine	2.0394599	0.276652163	4.9359E-09
[M+H+Na]2+	G1s neuac(2-3) a	1.2277873	0.478043193	6.3529E-09
[M+H]+	Met-Tyr-Arg	1.5217605	0.629030244	6.5443E-09
[M+H]+	Pheophorbide a	2.3348556	3.419856779	6.6449E-09
[M+H-H2O]+	Lewis a trisaccharide	1.3601317	2.122892344	6.7157E-09
[M+H]+	Lys-Ala-Lys	0.8420206	2.012959554	9.3757E-09
[M+H-C3H5Cl]+	Tri(3-chloropropyl) phosphate	1.0722154	3.034946898	1.1783E-08
[M+H]+	Phe-pro	3.177625	2.051852898	1.2428E-08
[M+H-C5H9O7P]+	Cytidine 5'-monophosphate	2.0261906	0.59108309	1.7024E-08
[M+Cl]-	1-pentadecanoyl-sn-glycero-3-phosphocholine	0.8682551	2.370225602	2.006E-08
[M+Na]+	Arctiin	0.9231606	2.262499134	2.1135E-08
[M-H]-	D-ribose 1-phosphate	3.8729054	0.095199464	2.2673E-08
[M+Na]+	Codeine-6-.beta.-d-glucuronide	1.4976067	2.156937321	2.6287E-08
[M+H]+	Fluphenazine	1.6333413	0.62753992	2.6564E-08
[M+H]+	Cytarabine	1.8739467	2.644815246	2.6969E-08
[M+H]+	Virginiamycin component a	1.4154159	10.3928586	2.8456E-08
[M+Na]+	Cysteine conjugated chenodeoxycholic acid	1.4510078	3.962625965	2.8704E-08
[M+H]+	Asp-Glu-Lys	1.532813	1.494547516	2.9225E-08
[M+K]+	Glycocholic acid	4.1025413	2.011399378	3.0022E-08
[M+H]+	[glu1] trh	1.8136258	0.652212037	3.1019E-08
[M+H]+	Gln-Tyr-Arg	1.5101183	1.975026199	3.1086E-08
[M-H]-	Guanosine 3',5'-cyclic monophosphate	2.1622292	0.076206465	3.7069E-08
[M-H]-	C.i. acid blue 90	0.982306	0.466510172	4.113E-08
[M-H]-	Asp-Glu	2.7574654	2.02702212	4.3074E-08
[M+H]+	N-demethylerythromycin	4.3590901	3300.817823	4.3748E-08
[M-H]-	Dl-mevalonic acid lactone	1.1163302	0.110175345	5.3473E-08
[M+H]+	Adenosine	34.200966	0.001035184	6.1493E-08
[M+H]+	Clindamycin	0.9619073	0.301723393	6.1797E-08
[M+H]+	Nodularin	0.661387	0.421088519	6.2476E-08
[M+H]+	Fenbendazole	3.5797156	0.227188319	6.2497E-08
[M+H]+	Pro-asn	4.1297841	0.878224421	6.594E-08
[M+Na]+	Prosapogenin a	0.9869064	3.35807168	6.8477E-08
[M+H]+	Quinaldic acid	2.9750065	0.380556257	6.9382E-08
[M+H]+	.gamma.-l-glu-.epsilon.-l-lys	3.2166573	2.234390612	7.0101E-08
[M+H-C10H10O2]+	Bisdemethoxycurcumin	1.7734327	2.643856479	7.1493E-08
[M-H]-	N-fructosyl s-(2-carboxypropyl)glutathione	1.6107792	2.211136567	7.7437E-08
(M+H-H2O)+	Lys-Val	2.4861606	1.673062941	1.0485E-07
[M+H]+	Dl-lanthionine	1.6623488	0.619104057	1.1752E-07
[M+H-NH3]+	D-glutamine	5.2669812	0.677253711	1.3777E-07
[M+H]+	Glu-Glu-Lys	1.9581788	1.672130747	1.4218E-07
[M+H-C6H10O4]+	N-.alpha.-(tert-butoxycarbonyl)-l-histidine	6.4452033	0.670416387	1.5208E-07
[M+H-C8H14O3]+	Erythromycin a enol ether	1.8765968	317.9653079	1.6571E-07
[M-H]-	3'-dephosphocoenzyme a	0.4380405	1.970957714	1.7466E-07
[M+H]+	Leu-Asp-Lys	0.6280544	0.663757608	1.9165E-07

[M+H]+	Pyrrol	0.8270956	2.719775513	2.0232E-07
[M-H]-	Glutathione ethyl ester	1.9246291	2.062961317	2.186E-07
[M+Na]+	Euphodendroidin m	1.5710506	1.953763202	2.1946E-07
[2M-H]-	Rac-2-despiperidyl-2-aminorepaglinide	1.0475943	2.594505888	2.3714E-07
[M+H]+	Pro-hyp	3.1329857	0.826894786	2.5949E-07
[M+H]+	15-deoxy-.delta.12,14-prostaglandin j2 glutathione	1.3253057	1.740569504	2.7521E-07
[2M-H]-	Oxyresveratrol	10.067054	0.001396233	2.9911E-07
[M+H]+	Clarithromycin	5.1296053	194.1640797	3.5315E-07
[M+H-C8H14O3]+	Pseudoerythromycin a enol ether	7.5123099	801.0268068	3.7661E-07
[M+H-C6H10O5]+	Neohesperidin	1.2303145	0.635125957	3.9276E-07
[M+H]+	L-threonine	2.2341369	0.766685917	4.409E-07
[M-H]-	Adenosine 3',5'-cyclic monophosphate	0.8689214	0.029674332	4.7615E-07
[M+H-C3H6O2]+	Meperidine	1.2031503	0.506183374	5.0243E-07
[M+H]+	Trp-Ile-Lys	0.3216125	0.579951127	5.055E-07
[2M+H]+	Valganciclovir	1.5681893	1.546464387	5.8727E-07
[M-H]-	4-hydroxyphenylacetic acid	7.4083109	0.722464345	6.1526E-07
[M+H]+	Glu-Gly-Lys	1.0232021	1.575941584	6.439E-07
[M-H]-	Cytidine 2',3'-cyclic phosphate	3.6740484	0.531420526	6.5922E-07
[M+H]+	N-oleoyl-d-erythro-sphinganine	0.4483392	6.94200633	6.6109E-07
[M+H]+	Histidine	8.1011354	0.655964118	6.6182E-07
[M+H]+	Pro-gln	1.9879818	1.320205989	6.8877E-07
[M+H]+	Aloeemodin	1.6192582	0.600375701	7.3991E-07
[M+K]+	Convallatoxin	0.9539845	1.559776372	7.9151E-07
[M+H]+	Tozasertib	2.1305315	4.24547932	8.5771E-07
[M+H-C5H8O]+	Trans-zeatin	2.3698439	0.46477936	9.0785E-07
[M-H]-	Guanosine 5'-monophosphate	2.2758477	9.2514045	9.2234E-07
[M+H]+	15.beta.-hydroxycyproterone acetate	2.0258007	0.690806713	9.3994E-07
[M+Na]+	Forskolin	2.1045391	1.161742957	9.4486E-07
[M-H]-	Tyr-Pro	1.1272409	2.37657759	9.7336E-07
[M+Na]+	5-methyltetrahydrofolic acid	1.8791603	1.802888698	9.8262E-07
[M-H]-	Glutathione, oxidized	1.1562318	0.355063338	1.078E-06
[M+H-C4H6SO]+	Captopril	3.6682962	1.423985367	1.0867E-06
[M+H]+	Phe-Pro-Lys	3.0217283	3.526125745	1.1531E-06
[M+H]+	Aconitine	2.2960628	2.021694773	1.1758E-06
[M+H]+	Spectinomycin	1.7686028	0.693837874	1.1849E-06
[M+H]+	Rifaximin	0.7390413	1.366519653	1.2189E-06
[M-H]-	Glu-Lys	1.6847658	1.662111459	1.4088E-06
[M+H]+	His-Asp-Lys	0.6527613	1.866752517	1.5559E-06
[M-H]-	4-methylphenol	3.205484	0.744039943	1.6468E-06
[M+H-C2O3]+	Psoralen	1.5212595	1.238146158	1.7661E-06
[M+H-H2O+2i]+	Chloramphenicol palmitate	0.7570348	2.019160813	1.8022E-06
[2M+Na]+	1,4-d-xylobiose	0.9277556	3.626807491	1.9222E-06
[2M-H]-	Ciclopirox .beta.-d-glucuronide	0.6663474	1.936755416	1.9403E-06
[M+H]+	Ser-Gln	0.8972492	1.525729866	1.9583E-06
[M+Na]+	Astrasieversianin xv	0.7808916	1.480827201	2.0577E-06
[M+H]+	Hydrocortisone 21-hemisuccinate	1.5596068	0.852242531	2.1195E-06
[M+H]+	Metconazole	3.766494	0.853852989	2.1857E-06

[M-H]-	Dl-a-hydroxybutyric acid	2.1137175	1.414567214	2.4377E-06
[M-H]-	Ser-Phe	1.8997656	1.69882432	2.7192E-06
[M+H]+	Pro-his	0.6944016	0.860154541	2.7657E-06
[M+H-H2O]+	Swertisin	0.4925427	0.600125897	2.8425E-06
[M+H]+	Pyroglu-Gln-Lys	4.6506617	0.692513256	2.8682E-06
[M-H]-	Dihydrothymine	1.6578194	0.663354352	2.8742E-06
[M+H]+	Bromocriptine	0.95753	1.199903278	2.9917E-06
[M+H-H2O]+	4-hydroxyphenethyl alcohol	1.7817483	1.268111676	3.0425E-06
[M+H]+	L-carnosine	1.1446249	1.561738782	3.3238E-06
[M+Na]+	Psychosine	1.2755396	0.758427335	3.5834E-06
[M+H]+	Pyroglu-trp-lys	0.8268364	1.409047589	3.8053E-06
[M+H]+	3'-o-methylinosine	1.1593385	2.00657267	4.2375E-06
[M+H]+	His-Glu-Lys	0.6207802	1.481471213	4.3085E-06
[M+H]+	Ala-Val	1.0570406	1.947543128	5.1292E-06
[M-H]-	Thalsimidine	0.6102715	1.902318584	5.41E-06
[M+H-3H2O]+	16-phenyltetranorprostaglandin e2	0.7945994	1.546210446	5.4786E-06
[M+H-2H2O]+	Penitrem a	1.088919	0.325224902	5.5838E-06
[M+H-C11H7ClN4O2]+	Eszopiclone n-oxide	2.3620536	0.496418862	5.9566E-06
[M+H]+	Arg-gly	0.4081855	0.794398334	6.2513E-06
[M+H]+	N-acetylglucosaminylasparagine	1.3000423	1.458962121	6.7536E-06
[M+H-2H2O]+	Ascomycin	1.7520404	5.519667981	7.7067E-06
[M-H]-	Glutamine	3.1755881	0.625560926	7.742E-06
(2M+K)+	Terbutaline	0.8916014	0.804484194	7.8166E-06
[M-H]-	2'-O-methylinosine	3.3550556	2.25022051	7.8947E-06
[M-H]-	Dethiobiotin	1.7497613	2.212189769	8.379E-06
[M-H]-	Glu-Leu	3.7945619	2.313341829	8.4368E-06
[M+H]+	.gamma.-aminobutyric acid	3.9179771	0.775450928	8.4604E-06
[M-H]-	Chebulinic acid	0.3304532	0.251227471	8.4682E-06
[M+H]+	Methionine sulfoxide	2.9209525	0.815583646	8.5869E-06
[2M-H]-	Cytidine 2',3'-cyclic monophosphoric acid	0.6155301	0.352373639	9.3259E-06
[M-H]-	N-oxalylglycine	4.2734713	1.389583342	9.452E-06
(M+H)+	L-Aspartate	0.9555894	1.3645095	9.6612E-06
[M+H]+	Methylphenidate	1.0893628	1.321864203	1.0047E-05
[M-H]-	Deoxyinosine	2.0397331	2.24697913	1.0484E-05
[M+H]+	Gln-Asp-Lys	0.9042687	1.325090554	1.0746E-05
[M+H]+	Ornithine	2.5239946	1.485843031	1.1523E-05
[M+H+2i]+	Jasplakinolide	2.9473579	1.464226893	1.1536E-05
[M+H-H2O]+	Dl-2,4-diaminobutyric acid	0.6888668	0.691394165	1.226E-05
[M+H-H2O]+	L-homoserine	1.5941357	0.779641765	1.2329E-05
[M+H]+	Candesartan cilexetil	1.5967388	0.803043635	1.4256E-05
[M-H]-	Chlorothricin	0.3955263	0.272999125	1.5042E-05
(M+H-H2O)+	His-Gln	3.8764619	0.809048019	1.6171E-05
[M+H]+	.alpha.-L-Asp-L-Lys	2.7207376	1.398044461	1.6413E-05
[M-H]-	2-chloro-2'-hydroxy-4'-methylbenzophenone	3.0316503	5.241970125	1.7737E-05
[M+H-C5H8O5]+	Repin	0.7399344	1.663720086	1.8675E-05
[M+H]+	Isoquinoline	1.147366	1.160378281	1.9142E-05

[M-H]-	Beta-hydroxybutyrate	1.8643661	0.778295433	1.9403E-05
[3M+H]+	Mildronate	0.3176559	0.532833335	1.984E-05
[M+H]+	S-adenosyl-l-homocysteine	0.9089832	0.721543485	2.0308E-05
[M+H]+	Anabaenopeptin a	1.1060606	0.748968382	2.059E-05
[M+H]+	Ng,ng-dimethyl-l-arginine	4.3029767	0.860302865	2.088E-05
[M-H]-	Linoleoyl-CoA	0.1946937	0.69692213	2.5138E-05
[M-H]-	Adenylyl(3'-5')cytidine	1.1549216	0.565494225	2.7015E-05
[M+H-C5H5ClO3]+	11,17-difuroatemometasone	2.2780408	0.849350252	2.7171E-05
[M-H]-	D-ornithine	2.7182557	1.464608147	2.7766E-05
[M+Na]+	Obacunone	1.8858718	0.89357749	2.8594E-05
[M+H]+	Arg-Ile-Lys	0.3210987	1.618413024	2.8929E-05
[M+Na]+	Isradipine	1.1473906	0.846136707	2.8954E-05
[M+H-CH6O2]+	Lappaconitine	0.9956696	0.643023585	3.2886E-05
[M+H]+	Cyclic gmp	1.4679422	0.082532905	3.3541E-05
(M-H)-	N-Acetylmannosamine	2.7582609	1.316949568	3.598E-05
[M+Na]+	NCGC00381123-01	0.6778702	0.883313268	3.6622E-05
[M+H-H2O]+	Prostaglandin a1-biotin	0.378953	1.467925586	3.9057E-05
(M+H-H2O)+	.alpha.-D-(+)-Talose	1.9864468	0.880278229	3.9527E-05
[M+H]+	Docetaxel	0.9226147	2.099141248	4.0476E-05
[M-H]-	3'-sialyllactose	0.7454376	0.809801271	4.0936E-05
[M-H-O3S]-	Taurolithocholic acid sulfate	0.7959889	0.768764123	4.1079E-05
[M-H+2Na]+	1-oleoyl-l-.alpha.-lysophosphatidic acid	0.578959	0.477334043	4.1546E-05
[M+H-C2H3NO]+	Isoprocarb	4.0108677	0.745387601	4.3475E-05
[M+H]+	Lys-Gln	0.8996505	2.109071094	4.6145E-05
[M+H]+	Lycopsamine	0.6739071	0.785894932	4.9573E-05
[M+H]+	Pepstatin a	1.141123	1.839325003	5.0952E-05
[M+H]+	4-deoxypyridoxine	0.4863024	0.857773567	5.3622E-05
[M-H-CO2]-	Gambogic acid	1.6194469	2.112801992	5.9258E-05
[M+H]+	Diprotin b	2.3539305	1.291701805	6.0906E-05
[2M+H]+	Vildagliptin	0.6088722	0.653438442	6.1032E-05
[M+H]+	Arg-phe	0.5422978	0.870511302	6.1453E-05
[M+H]+	Irbesartan	0.9056576	1.255895661	6.3134E-05
[M+H]+	Asp-Met	0.9040865	1.185128465	6.4994E-05
(2M+Na)+	Gemcitabine	0.6254416	1.320991624	6.7043E-05
[M+H-CH2O2]+	4-ketopimelic acid	1.5898296	0.898633633	7.4258E-05
[M+2H]2+	Phe-Asp-Lys	1.1218473	0.816611164	7.7117E-05
[M+H]+	Pro-arg	1.000602	1.513534614	7.7369E-05
[M+Na]+	Terracinolide c	0.550252	1.634932676	8.008E-05
[M-H]-	Udp-n-acetylglucosamine	0.4558315	1.460859246	8.3783E-05
[M+H]+	S-Adenosylmethionine	0.2969861	0.642058637	8.4862E-05
[M+H-NH3]+	Ala-Ala	2.8601847	1.183155433	8.5663E-05
[M+Na]+	Glimepiride	0.7274582	1.283802468	8.7249E-05
[M-H]-	3-(methylthio)benzoic acid	2.1295775	0.376658752	8.7438E-05
(M-H)-	2'-Deoxyuridine	1.7673276	1.251191185	8.7902E-05
[M-H]-	L-methionine	5.1396937	0.776563093	9.2995E-05
[M+Na]+	Kaempferitrin	1.3544222	0.700064494	9.3955E-05
[M-H]-	Tryptophan	20.452895	1.164976	9.7486E-05

[2M+Na]+	Flumetasone	0.7772905	0.635262257	0.00010392
[M+Na]+	N.epsilon.-acetyl-l-lysine	1.6070191	0.819162517	0.00010479
[M+H]+	Lewis y tetrasaccharide	0.5457888	1.223864554	0.00011216
[M+H-C14H12O10]+	Chicoric acid	0.2572259	1.150393261	0.00011336
[M-H]-	Thiazolidine-2-carboxylic acid	1.1127159	1.240645781	0.00011691
[M-H]-	2-keto-3-deoxyoctonic acid	1.4298447	0.263852227	0.00012299
[M+H-C3HF3N2OS]+	Flufenacet	1.1797278	0.569355585	0.00012316
[M-H]-	Ser-Lys	0.7693077	1.404492852	0.00012445
[M+H]+	Ala-Glu-Lys	1.197419	2.818452629	0.00012572
[M-H]-	Uridine 5'-monophosphate	2.3583722	1.575480494	0.0001373
[M+K]+	Lnnt	0.3804883	1.601096021	0.00014028
[M+H]+	Stachydrine	1.058717	0.886203497	0.00014184
[M+H-H2O]+	Cimicifugoside h 2	0.591379	0.824713091	0.0001478
[M+H-CO2]+	Indole-3-carboxylic acid	2.5527596	1.151516858	0.00015327
[M-H]-	2-oxoadipic acid	0.6041989	0.803422716	0.00015767
[M+H]+	Lasiocarpine	0.8089879	0.876084643	0.00015911
(M+H-2H2O)+	Phe-Lys	0.678173	0.873274398	0.00016181
[M+H-NH3]+	1-acetyl-3-piperidinamine	0.5215494	0.890540603	0.00016223
[M+H]+	Leu-Lys-Lys	0.8038739	1.584967996	0.00016955
[M+H]+	Cantharidin	1.2120481	0.809430542	0.00017157
[M+Na]+	Cabergoline	0.8078381	1.854922553	0.00017437
(M+CH3CN+H)+	L-Norleucine	1.4428143	0.616811998	0.00017697
[M+H]+	Pro-pro	4.5488951	1.473784033	0.00018932
[M+Na]+	Genipin-gentiobioside	0.4530337	1.598829334	0.00019134
[M+H]+	Acetaminophen sulfate	1.2666932	1.390257285	0.00019449
[M+Na]+	Stevioside	0.3280605	1.205616166	0.00019798
[M+H]+	2-phenylpiperidine-2-acetamide	1.1089181	0.838211429	0.0001992
[M-H]-	4-imidazoleacrylic acid	1.6561034	0.632749883	0.00020109
[M+H]+	G-guanidinobutyrate	1.7185836	0.908953015	0.00020896
(M+CH3CN+H)+	Gamma-Glutamylcysteine	1.5116817	0.830353392	0.00021026
[M+Na]+	Angeloylgomisin h	0.610842	1.552519243	0.00021162
[M+H]+	Ala-Pro-Lys	2.3285125	6.534477603	0.00022564
[M+Na-H2O]+	2'-fucosyllactose	1.1924444	0.867103678	0.00024073
[M+H]+	Pro-Val-Lys	1.0353698	1.45189553	0.00024962
M+	Trp-Phe	0.4765952	0.623617491	0.00025322
[M+H]+	Asp-Asp	0.7182383	1.18329993	0.00026945
[M-H]-	Buprenorphine glucuronide	0.5410833	2.138254225	0.00028478
[2M-H]-	Lividic acid	0.3681972	1.626098347	0.00029373
[M-H]-	N-acetylneuraminic acid	2.5212651	1.275297191	0.00029786
[M+H]+	Ala-Ile	0.6676825	1.700604789	0.00029827
[M+H]+	Val-Asp-Lys	1.5007968	1.563186513	0.00030051
(M-H)-	L-Valine	1.0942225	0.675003457	0.00030455
(M+CH3COO+2H)+	Diethylcarbamazine	1.1436567	0.818813499	0.00031826
[M-H-H2O]-	D-glucose 6-phosphate	0.3188394	2.00955714	0.00034743
[M+H]+	Pyroglu-Glu-Lys	2.0052373	0.87869464	0.00036985

[M+H]+	Pyroglu-Gly-Lys	1.7987273	0.838801052	0.00039443
[M+Na]+	Pyripyropene a	1.1735181	0.839435443	0.00039895
[M+H]+	Methyldopa	3.2502691	0.870477633	0.00043035
[M+H]+	Neomycin	1.1237516	1.810413179	0.00043053
[M+H-C2H5N]+	4-(1-piperazinyl)-1h-indole	2.2953997	1.145326289	0.00049296
[M+H]+	Pro-Ala-Arg	1.7254749	1.577772495	0.00049395
[M-H]-	Lignoceroyl coenzyme a	0.1932342	0.813090717	0.00049968
[M+H]+	Agomelatine	2.1612479	0.918967779	0.00050575
[M+H-2H2O]+	(+)-pinoresinol	1.2312622	0.797368019	0.00051349
[M+H]+	Met-Phe	0.9542611	0.786115857	0.00057808
[M-H]-	Beta-D-Fructose 6-phosphate	0.6556814	0.703699386	0.00058292
(M+H-H2O)+	His-Thr	2.9374011	1.210242728	0.00058721
[M+H]+	Pyroglu-pro-lys	2.2972048	0.932956948	0.00059389
[M+H]+	Pyroglutamylglycine	1.0835677	0.866586395	0.00062063
[M-H]-	Lysine	11.234449	0.75534078	0.00062641
[M+H]+	Leu-Gly-Lys	0.5751493	0.89338962	0.00063758
[M+H]+	Rescinnamine	0.8298634	1.185369898	0.00064279
[M+H]+	Trigonelline	4.4273129	0.852826088	0.00065141
(M-H)-	Urocanic acid	0.5262212	0.781718167	0.00065462
[M+H]+	Asn-Val-Lys	0.5022736	1.207028622	0.00065538
[M-H-CO2]-	Indoleacrylic acid	2.0431478	1.139216895	0.00065583
[M-H]-	[(2r,3s,4s,5r,6s)-6-[3,5-dihydroxy-4-[3-(4-hydroxyphenyl)propanoyl]phenoxy]-3,4,5-trihydroxyoxan-2-yl]methyl 3,4,5-trihydroxybenzoate	0.6074321	0.690475695	0.00068745
[M-H]-	Ile-Tyr	0.5841835	1.485558768	0.0007107
[M-H]-	Thr-Val-Leu	1.6272405	1.458172817	0.00072046
[M+H]+	Dibutyl phthalate	0.5692447	0.789016667	0.00074081
[M+H]+	(2s,3s)-3,5,7-trihydroxy-6-methyl-2-(3,4,5-trihydroxyphenyl)-2,3-dihydrochromen-4-one	0.9976514	0.872521037	0.00076104
[M+H]+	N.epsilon.-methyl-l-lysine	1.2837826	0.890585696	0.00079196
[M-H]-	Doxorubicin	0.4482784	1.603250085	0.00079334
[M+H-H2O]+	4-hydroxy-l-glutamic acid	1.0426385	0.795908414	0.00080316
[M+H]+	Pro-Gly-Pro	1.2389058	0.731077273	0.00082109
[M+H]+	Pro-leu	2.0870059	0.81674952	0.00082622
[M+H]+	L-homocitrulline	1.1405905	0.867867154	0.00083709
[M+H]+	N6,N6,N6-Trimethyl-L-lysine	3.5091483	1.129666822	0.00084556
[M+H]+	Ser-Asp	0.9572804	0.874974956	0.00085296
[M+H-H2O]+	Glutaraldehyde	0.7512782	0.906736815	0.000872
[M+H]+	Aloesin	0.4550675	0.732989386	0.00087746
[M+H]+	Pyroglu-ser-lys	1.0055486	0.858482121	0.00088746
[M+H]+	Fruleuile	2.3336329	1.19154544	0.00089397
[M+H]+	Benzoylmesaconine	2.2429642	0.914436781	0.00092372
[M+Na]+	1-palmitoyl-2-glutaryl phosphatidylcholine	0.2297984	1.39357354	0.00093041
[2M-H]-	1h-indole-1-butanoic acid, 3-(1-naphthalenylcarbonyl)-	0.3966903	0.595485632	0.00093637
[M+H]+	Leu-Ser	1.3108401	1.576635099	0.0009901
[M+H]+	.alpha.-amanitin	0.7019855	0.636050934	0.00099421
[M-H]-	Malonic acid	0.3612555	0.883869001	0.00102943

[M+H]+	Morphine-3-glucuronide	0.3658507	0.836678751	0.00108931
[M-H]-	4-aminobenzoate	0.882913	0.78580047	0.0010899
[M+H]+	Gamma-glu-glu	0.7554014	0.896607073	0.00109155
[M-H]-	2,6-di-tert-butylphenol	2.5225847	0.760826516	0.00109327
[M+2H]2+	Val-Thr-Arg	0.5408447	0.664012364	0.0011275
[M+H]+	5-aminovaleric acid betaine	1.8488524	0.874607816	0.00116484
[2M-H]-	Rhapontigenin	0.9414851	0.761044544	0.0011742
[M+H]+	Fusarinin c	0.1793018	1.717500608	0.001182
[M+H]+	Pro-Pro-Lys	1.2679602	1.207856818	0.00118342
[M+H]+	Pyridoxamine	0.5708177	0.784777021	0.00118518
[M+H]+	Pidotimod	2.2806809	0.789425774	0.00120001
[M+H]+	Leu-Val-Lys	0.6559207	1.360193182	0.00121198
[M-H]-	[(6e,10z)-6-formyl-10-(hydroxymethyl)-3-methylidene-2-oxo-3a,4,5,8,9,11a-hexahydrocyclodeca[b]furan-4-yl] 3-methylbutanoate	0.5925947	0.661513351	0.00122484
[M-H]-	3-hydroxyglutaric acid	1.560993	0.464624089	0.0012646
[M+H-NH3]+	2-thiophenecarboxamide, 3-[(aminocarbonyl)amino]-5-(3-fluorophenyl)-n-(3s)-3-piperidinyl-	0.9736341	1.831975843	0.00128049
[M-H]-	Esculetin	1.9036234	1.351904791	0.00128532
[M+Na]+	Acetyl isogambogic acid	0.6057566	0.879477594	0.0012993
[M+Na]+	1-(1,2-dihexanoylphosphatidyl)inositol	0.8454919	0.800754141	0.00129943
[M+H]+	Asp-His	1.1951081	0.826128235	0.00132392
[M+H]+	Harringtonine	0.7395541	1.138983424	0.00133529
[M-H]-	D-Galactarate	1.58181	0.784227204	0.00133589
[M-H]-	Cyclo[3-(2-naphthalenyl)-l-alanylglycyl-d-tyrosyl-l-arginyl-l-arginyl]	3.0032903	1.438480636	0.0014056
[M-H]-	Thr-Glu	1.3459826	1.302056919	0.00143847
[M+H]+	L-cystathionine	0.7651276	0.828137494	0.00146073
(M+H)+	Allopurinol riboside	0.3215585	1.156132525	0.00147572
[M-H-H2O]-	2-keto-l-gulonic acid	1.589558	0.510921253	0.00148195
[M+H]+	Ile-Asn	0.5554095	1.253702829	0.00148929
[M+H]+	Reserpine	1.0190731	2.170131192	0.00149654
[M+H]+	Glu-Thr-Lys	0.6365076	0.867660677	0.00150607
[M+H]+	Ganoderic acid h	1.3385412	2.708034896	0.00152824
[M+H]+	Norvaline, 5-phosphono-	0.499887	0.888570198	0.00164982
[M-H]-	Kynurenic acid	1.2476242	1.217171289	0.00165182
[M-H-H2O]-	Taurodeoxycholic acid	0.6356746	1.470955662	0.00166068
[M-H]-	Biotin	7.0672049	0.044610127	0.0016609
[M+H]+	7-hydroxymitragynine	0.6256612	1.096080125	0.00168406
[M+H]+	D-lactose	3.4586034	0.846738318	0.00169857
[M+H-NH3]+	6-hydroxymelatonin	0.9115239	0.673063034	0.00172988
[M+H-H2O]+	Leukotriene c4	1.1007756	1.101202145	0.00184064
[M+Na]+	Fissinolide	0.5191347	0.903476415	0.00184871
[M-H]-	N-acetyl-l-aspartic acid	0.6277035	0.887882301	0.00186678
[M+H]+	Val-Glu-Arg	0.1887947	0.591455745	0.00193559
[M+Na]+	Condelphine	0.8381579	1.431903795	0.00200241
[M+H]+	Arg-Asp-Arg	0.9401569	2.869051356	0.00204535

[M-H]-	N-fructosyl isoleucine	0.7212113	0.868532691	0.00205921
[M+H-H2O]+	Acarbose	0.5596857	1.326374623	0.00206332
[M+H]+	Cis-jasmone	0.7796605	0.755098401	0.00208024
[M-H]-	Glu-Gly-Glu	1.017327	1.229110839	0.00214129
(M-H)-	D(-)-beta-hydroxy butyric acid	0.5854821	1.25407051	0.00224449
[M+Cl]-	1h-indazole-1-pentanoic acid, 3-[(tricyclo[3.3.1.1(3,7)]dec-1-ylamino)carbonyl]-	1.4971796	0.325024519	0.00227026
[M+Na]+	Antibiotic k 252b	0.3336951	0.890089115	0.00230728
[M-H]-	Ltc4-[d5]	0.2781048	1.335880609	0.00230967
[M+H]+	Pro-Gln-Arg	0.551999	0.872271485	0.00234203
(M+H-H2O)+	Lys-Ser	2.0075785	0.879190475	0.00237011
[M+H]+	Phe-tyr	0.3676304	1.278793941	0.00243237
[M+Na]+	1-o-hexadecyl-2-deoxy-2-thio-s-acetyl-sn-glyceryl-3-phosphorylcholine	0.9997652	0.857629026	0.00251196
[M+H]+	N-trifluoroacetyl deacetylcolchiceine	0.7453832	0.91748521	0.00251237
(M+NH4-2H)-	Phosphocreatine	1.2816323	0.16917567	0.00252044
[M+H]+	Mycophenolate mofetil	0.8533169	2.001818836	0.00255312
[M+H-H2O]+	Swainsonine	0.4608187	0.874917844	0.0025687
[M+H-NH3]+	Eflornithine	0.6197275	0.898566679	0.00259113
[M+H]+	Ser-Glu-Lys	1.577159	2.154909594	0.00259716
[M+H]+	Anthranoyllycoctonine	0.1548069	1.414015695	0.00265613
(M+NH4-2H)-	alpha-D-Glucose 1-phosphate	0.4321493	0.781736806	0.00269993
[M+H+i]+	Ala-Arg	1.5366661	0.872336147	0.00271143
[M+H]+	Asp-Met-Arg	0.6899778	1.689860207	0.00280194
[M-H]-	Malate	1.7366887	0.887547968	0.00298423
[M+Na]+	Paeonolide	0.8128849	0.903205914	0.00303419
[M+H]+	4-tert-butyldimethylsilyloxynebivolol	1.6608308	0.869066029	0.0030455
[M+H]+	Pyroglu-thr-lys	1.1322759	0.773798109	0.00317208
[M+H]+	Lys-lys	1.331149	1.495269237	0.00326068
[2M-H]-	DL-tryptophan	5.3054273	1.192294002	0.00328614
[M-H]-	3-methyl-4-nitrophenol	0.5263395	0.559799614	0.0033026
(M+CH3COO)-	Acetyl-L-Cysteine	0.2714086	0.792462678	0.00338177
[M-H]-	N-acetyl-dl-serine	1.6654802	1.342737488	0.00338382
[M-H]-	Acetylcysteine	3.1817708	0.786701311	0.00344364
(M+H-2H2O)+	Desipramine	0.2168912	1.460156215	0.00346322
[M+H]+	Eprosartan	0.6277522	0.810252676	0.00347229
[M+H]+	Prim-o-glucosylcimifugin	0.5777579	0.808583674	0.0034818
[M+Na]+	Puromycin	0.5389294	0.697513421	0.00349817
[M+H]+	Aldosterone	1.1341797	0.580887037	0.00357576
[M+H]+	Methyl 4,6-o-benzylidene-.alpha.-d-glucopyranoside	0.7065619	0.706983757	0.00358167
[M-H]-	2,3-dideoxyuridine	1.7246936	0.859895441	0.00368768
[M+H]+	Nicotinate	2.7242866	0.90736394	0.00380551
[M+Na]+	(2e,6e,11e,13e)-18-(2,6-dioxopiperidin-4-yl)-9-hydroxy-8-methoxy-10,12,14-trimethyl-15-oxooctadeca-2,6,11,13-tetraenoic acid	1.5914705	0.744194166	0.00382594
[M+H-CH3ON]+	L-homoarginine	0.6852392	0.898216105	0.00387232
[M-H]-	3-aminohexanoic acid	0.4045798	0.867802319	0.00394032

[M+Na]+	Herbimycin a	0.6906478	0.804800982	0.00396644
[M+Na]+	3-deacetylsalannin	0.7067855	1.243642032	0.00397421
[M+H]+	Asp-Asn	0.9981988	0.813386134	0.00412718
[M+H]+	Gly-Asp	0.7535528	0.869713839	0.00416773
[M-H]-	Pantothenate	2.0800439	0.894362852	0.00422132
[M+H]+	Gly-Trp	0.3969999	1.276569115	0.00422375
[M+H]+	Pyroglu-Ala-Lys	0.9065472	0.725571739	0.00438479
[M+H]+	Ethyl gallate	1.3089064	1.138933569	0.00455318
(M+K)+	Tyr-Phe	0.3445646	0.633546102	0.00457201
[M+Na]+	Phytolaccasaponin G	1.0266032	1.813622963	0.00465832
[M+Cl]-	1-oleoyl-sn-glycero-3-phosphocholine	3.1157612	1.285960519	0.00471151
[M+NH4]+	Lewis x trisaccharide	1.4275238	0.867146181	0.00493518
[M-H]-	D-erythrose 4-phosphate	0.7857406	0.735545044	0.00518843
[M+H]+	Ile-Val	0.7486632	1.352891037	0.00520212
[M+H]+	Acadesine (drug)	0.832877	0.858370066	0.00528823
(M+H-H2O)+	Ile-Glu	6.6724392	0.912493977	0.00535047
[M-H]-	Phenylacetic acid	10.176241	0.640423	0.00541213
[M-H]-	Leukotriene e4	0.9528641	1.285961394	0.0055089
[2M+H]+	Cumyluron	0.8474054	0.687704298	0.00554651
[M+H]+	Pyroglu-val-lys	1.6532424	0.919604241	0.00559502
[M+H]+	4-imidazoleacetic acid	0.467696	1.355346223	0.00559553
[M+H]+	Gln-Ala-Lys	0.9151122	0.645062483	0.00562695
[M+H-H2O]+	Cytochalasin e	1.1991631	1.23849454	0.00572452
[M+H]+	Triamcinolone diacetate	0.7891694	0.781623884	0.00591045
[M+H]+	Simmondsin	0.9333327	1.169864288	0.00601802
[M+H]+	Sinefungin	0.2440469	0.533740813	0.00608328
[M+H-NH3]+	Doxycycline	0.2243519	0.919373348	0.00610776
[M-H]-	Kushenol f	0.9533913	0.824279976	0.00618324
[M+H]+	Leukotriene f4	1.043068	0.8268876	0.00636505
[M+2H]2+	Phe-Asp-Arg	1.7525662	0.753756266	0.0063709
[M-H]-	Ser-His	0.9624177	1.365921542	0.00639237
[M+H-H2O]+	Proscillaridin a	0.4493562	0.925511089	0.00653464
[M+H-H2O]+	Batimastat	0.8032758	0.840740606	0.00654924
[M+2H]2+	His-His-Arg	3.2449093	0.914813299	0.00656528
[M+H]+	Gln-leu	0.7308526	0.698945982	0.00691599
[M+H]+	Gly-Asn-Arg	2.4344918	0.677432539	0.00693451
[M-H]-	Pantetheine	1.6766311	0.549538449	0.00697232
[M-H]-	Thr-Lys	0.376722	0.702420117	0.00699459
[M+H-C5H8O3]+	5-methyl-2'-deoxycytidine	0.8679618	0.418352132	0.00705949
[M+Na]+	Fluvastatin	4.0499691	0.931385324	0.00707116
[M+H-H2O]+	Benzenepropanoic acid, 5-(3-carboxybenzoyl)-2-[[(5e)-6-(4-methoxyphenyl)-5-hexen-1-yl]oxy]-	0.5960506	0.87434286	0.00714472
[M+H]+	Gly-Glu-Lys	0.5796909	0.842846759	0.00715077
[M+2H]2+	His-His-Lys	0.9356235	0.836932298	0.00715415
[M+H-NH3]+	1,5-pentanediamine	0.5711535	0.876411763	0.00719543
[M+H]+	Trilostane	2.0039163	0.721232013	0.00756083
[M-H]-	Succinimide	0.3475123	0.802009811	0.00788401

[M+H]+	Thiazolidine-4-carboxylic acid	1.1610966	0.88795124	0.00792348
[M+H]+	Thr-His	0.4769604	0.873893215	0.00794535
[M-H]-	N-.alpha.-(tert-butoxycarbonyl)-l-proline	0.6276219	0.83303375	0.00801456
[M+H]+	Ile-Ala	0.3259315	1.113166707	0.00811921
(M+H-H2O)+	Gly-His	1.4454988	1.112002067	0.00817964
[M+H]+	3-methyl-3,4-dihydro-1h-1,4-benzodiazepine-2,5-dione	2.4819605	0.803926601	0.00820888
[M+H]+	4-hydroxy-l-phenylglycine	0.7949481	0.884979128	0.00830068
[2M+H]+	7-hydroxy-2-acetylaminofluorene	0.4659928	0.817805249	0.00839423
[M-H]-	Antipain	0.6511802	0.842785742	0.00851176
[M+H]+	Erythromycylamine	0.0905996	1.415916653	0.00854324
[M+H]+	Yunaconitine	1.126655	1.693737512	0.00855666
[M+H-NH3]+	3-methoxytyramine	0.2969266	0.853472562	0.00861707
[M+H]+	Pro-Ser-Arg	0.8532639	1.378280653	0.00862483
[M-H]-	Garcinol	1.2648206	0.877296151	0.00866483
[2M-H]-	Norfloxacin	0.9082103	0.738131446	0.00870662
[M+H]+	Pyroglu-trp	2.759676	0.916935549	0.0088915
(M+NH4-2H)-	Bestatin	2.1915002	1.792305529	0.00891659
[M+H]+	3-hydroxybutyrylcarnitine	0.646335	0.871316945	0.00891956
[M+HCOO]-	Deoxynivalenol-3-glucoside	2.4810399	1.144974028	0.00905795
[M+H-H2O]+	Prostaglandin e2 p-acetamidophenyl ester	0.33721	0.900957528	0.00937265
[M+H]+	3-aminoquinoline	0.797599	1.099816813	0.00956468
[M-H2O+H]+	L-leucine, n-acetyl	1.2183537	0.814172387	0.00987128
[M+H]+	Beclomethasone	0.8560999	0.906908389	0.00992463
[M+H-C10H11ON]+	Syrosingopine	0.556211	0.78362371	0.00994206
[M+Na]+	Crustecdysone	0.8631982	1.637008536	0.01004697
[M+H]+	Ketanserin	0.5881316	1.252329535	0.01021585
[M+H]+	Homoharringtonine	0.5068148	1.343235787	0.0103351
[M+H]+	Ergocristam	0.4375125	1.28089511	0.01043167
[M-H-H2F2]-	Fulvestrant 9-sulfone	0.2181962	0.878605705	0.01052672
[M-H]-	Aloin	0.3592157	0.678825873	0.01054598
[M+H-NH3]+	Thyrotropin-releasing hormone	1.1812385	0.682981823	0.01056948
M-	Salicylamide	0.5192618	0.743415628	0.01060168
[2M+H]+	1-(4-fluorobenzyl)-n-(naphthalen-1-yl)-1h-indole-3-carboxamide	0.3549752	0.824422564	0.01062865
[M+H-NH3]+	Tyramine	1.8752135	0.853583341	0.01097265
(M+CH3CN+Na)+	Ile-Phe	0.5045523	1.625291565	0.01116647
(M-H2O-H)-	myo-Inositol	1.821927	0.874706664	0.01126559
[2M-H]-	Methanone, (6-hydroxy-1-pentyl-1h-indol-3-yl)-1-naphthalenyl-	0.5457723	1.205378261	0.01148106
[M+H]+	Leu-Glu	0.9073188	0.856494674	0.01157439
[M+Na]+	1,7-bis(4-hydroxyphenyl)-5-[(2r,3r,4s,5s,6r)-3,4,5-trihydroxy-6-(hydroxymethyl)oxan-2-yl]oxyheptan-3-one	0.6737673	1.722191492	0.01192008
[M+H]+	N6-(1-iminoethyl)-l-lysine	1.1237997	0.77760715	0.01195962
[M+H-H2O]+	Leu-Val	0.9921298	1.44004126	0.01206278
[M-H]-	Pi 40:6	0.3664809	0.545417663	0.01223986
[M+H]+	Flunisolide	0.6383113	0.926235653	0.01265081

[M+Na]+	Norbuprenorphine glucuronide	0.7230744	0.857726773	0.01269063
[M+H]+	Dihydrocapsaicin	0.423146	0.664477549	0.01306172
[M+Na]+	Dihydrofolic acid	0.8329682	0.839771554	0.01326479
[M+H]+	4-hydroxy-1-(2-hydroxyethyl)-2,2,6,6-tetramethylpiperidine	4.8050345	0.79014427	0.01363207
[M+H]+	Dronedarone	0.3560649	0.805535568	0.01368426
(M+Na-2H)-	Stavudine	0.3520889	0.915365801	0.01390158
[M-H-C2O3]-	Benazolin	1.131389	0.783076184	0.01425642
[M+H-C7H6N2]+	1,5-naphthyridine, 2-[3-(6-methyl-2-pyridinyl)-1h-pyrazol-4-yl]-	0.2170947	1.13802957	0.01465087
[M+H]+	.delta.-octalactone	0.2475528	0.893935445	0.01476056
[M+Na]+	Mitraphylline	0.7145654	0.839904037	0.01504879
[M+H]+	Gly-Lys	0.4767435	0.866884607	0.01508539
[M-H-C6H6O6]-	Dehydro-l-(+)-ascorbic acid dimer	1.4168723	0.66678503	0.01518332
[M+H]+	Disulfiram	0.3869889	1.215265328	0.0152702
(M+H)+	Lys-Pro	1.2941802	2.320994801	0.01530492
[M-H]-	Labetalol	1.2744011	0.836376527	0.01533747
[M+H-C10H16O13N5P3]+	Acetyl coenzyme a	1.6712599	0.791678527	0.01535496
[M-H]-	3'-cmp	0.2924116	1.185685747	0.01540779
[M+H]+	Chlorhexidine	1.0390677	0.908180109	0.01545121
[M-H]-	Maleic acid	0.7770762	0.91389122	0.01576514
[M+H-C4H9NO2]+	N-phenylacetyl-l-prolylglycine ethyl ester	0.4683206	1.119546542	0.01596995
(M-H)-	3-methylcytidine	0.3294414	0.627851908	0.01623037
[M+H-2H2O]+	Calcimycin	0.553142	1.302697147	0.01649653
[M+Cl]-	Asiatic acid	1.1914046	1.189345489	0.01652656
[M+H-H2O]+	1,2-dioleoyl-sn-glycerol	0.552223	1.65831557	0.01664777
[M+K]+	Gentianose	0.3664654	0.860387251	0.01665254
[M-H]-	Isorhapontin	0.6423328	1.129308212	0.01679311
(M+NH4)+	Tyr-Ala	0.3224783	1.233114554	0.01694003
[M-H]-	Gdp-l-fucose	0.3968732	0.729700481	0.01718006
[M+2H]2+	Lys-Trp-Lys	0.4147436	0.895854888	0.01742527
[M-H]-	Corilagin	0.2449079	0.745088993	0.01748238
[M+H]+	D-glucosamine 6-phosphate	0.4889591	0.891204544	0.01757381
[M+Na]+	Fumonisin b1	0.4776217	0.936245528	0.01759756
[M+H]+	Mesaconitine	0.6088168	1.66776134	0.01763812
[M+H]+	Thr-Val-Lys	0.5730641	1.521450952	0.01766643
[M+H]+	Didodecyl 3,3'-thiodipropionate oxide	3.5401748	2.109965396	0.01790829
[M+CH3COOH-H]-	Haploperoside c acetate	0.211118	0.69962806	0.01801031
[M+H]+	Gamabufotalin	0.5407231	1.094489501	0.01803729
[M+H]+	Leu-Pro	1.5236209	0.928400288	0.01811151
(M-H)-	Uridine 5'-monophosphate (UMP)	0.633102	1.143056922	0.01820414
[M+FA-H]-	Gentiopicroside	1.1120387	0.90676516	0.01825018
[M+H]+	Gly-Ser	0.6315507	0.898318193	0.01841069
[M+H]+	Anorexigenic peptide	0.784053	0.820195093	0.01849694
[M-H-H2O]-	Geranyl diphosphate	1.1889051	1.691967108	0.01858179

[M+H]+	Acetamide, n-[2-[5-(acetyloxy)-3,6-dimethoxy-1-phenanthrenyl]ethyl]-n-methyl-	0.8836367	1.201352972	0.01877341
[M-H]-	Hematoporphyrin	0.7226938	1.643077743	0.01890705
[M+H-C2H4O2]+	Hypaconitine	1.3199209	0.738184203	0.0192685
(M+H)+	L-Isoleucine	0.5934518	0.899706352	0.01950121
[M+2H]2+	Pro-His-Arg	0.5099889	0.68404834	0.01959023
[M+HCOO]-	S4:18(p3:16/f1:2)	0.6261786	0.542821132	0.01967651
[M-H-C8H12O]-	Nabilone	0.4461151	0.768313123	0.0199967
[M-H]-	Ile-His	0.7120974	1.109139923	0.02005486
[M+Na]+	Scopolin	2.0666945	0.607775242	0.02038413
[2M+H]+	DL-proline	0.4512842	0.920231701	0.02049243
[M+H]+	Met-Pro-Lys	0.7211582	0.633410662	0.02116755
(M+CH3COO)-	Citramalic acid	0.6158731	0.757752714	0.02192215
[M-H]-	1,3,5(10)-estratrien-3,17.beta.-diol diglucosiduronate	0.8418914	1.476265985	0.02234149
[M+H-H2O]+	Cellobiose	4.062336	0.822913515	0.02245078
(M-H)-	2-Methylbenzoic acid	0.9217649	1.455997993	0.02246246
[2M+Na]+	4.alpha.-mannobiose	0.7105509	0.898410878	0.02247272
[M+H]+	Aerucyclamide a	1.2385739	0.297413765	0.0225137
[2M-H]-	N-acetyl-s-farnesyl-l-cysteine	1.3872739	0.709084313	0.02261975
[M+H]+	5-Amino-4-carbamoylimidazole (AICA)	0.5350635	1.096601761	0.02336542
[M+H]+	Histamine	0.8428425	0.909500348	0.02341083
[M+H]+	Pyroglu-Asp	0.5291066	1.159218166	0.02387546
[M+H]+	Pyroglu-tyr	1.7129152	0.910217535	0.02390594
[M-H]-	Alanine	2.1035599	0.783503303	0.02393312
[M+H]+	Arg-his	1.3205514	0.754893334	0.02409187
[M-H]-	Raffinose	1.0390495	0.892091685	0.02446921
[M-H]-	Glyphosine	0.5584908	0.778734645	0.02469802
[M+H]+	Atrazine	0.2938601	1.234299836	0.02491899
[M+H]+	D-pyroglutamic acid	3.3877105	0.934093392	0.02494411
(M+H-2H2O)+	Phe-Phe	0.9901555	0.483414604	0.02505033
[M-H]-	Glyceric acid	0.3093103	0.774304559	0.0252509
[M+H-H2O]+	3-(2-hydroxyethyl)indole	0.501566	1.15102121	0.02537759
[M+H]+	Carboline base + 4h, carboxylic acid	0.8997667	0.909253057	0.0255034
[M-H]-	Alpha-d-glucose	5.9809052	0.875298409	0.02577754
[M-H]-	Indole	2.6356195	1.082288335	0.02593272
(2M-H)-	3-Hydroxyisovaleric acid	2.3294256	0.874948268	0.02616585
[M-H]-	Hydroxyisocaproic acid	4.4930008	1.120190757	0.02618544
[M+H-H2O]+	Ancymidol	0.3923805	0.878714262	0.02633104
[M-H]-	D-proline	3.3007497	0.919848334	0.02664643
(M+CH3CN+Na)+	Arg-Cys	0.3560131	0.784897416	0.02719465
[M+Na]+	N,n',n''-triacetylchitotriose	0.7750723	1.655939353	0.02756969
[M+NH4]+	6-acetylacteoside	2.8689705	0.877435166	0.02802292
[M+H]+	N-acetylcarnosine	0.3730746	0.894883038	0.02812865
(M-H2O-H)-	5-L-Glutamyl-L-alanine	2.7752922	0.676532175	0.0287049
[M+H]+	Asn-Ile	0.7635725	1.175628871	0.02874802
[M+H]+	N-acetylhistidine	0.4252098	0.880685679	0.02915362
[M+H-C16H30O2]+	Glyceryl tripalmitoleate	0.2965114	1.731396595	0.02942593

[M+H]+	Tyrosine	0.6638112	0.938678731	0.02943386
[M+H]+	Ile-Pro-Ile	1.0864814	1.303052304	0.02950404
[M-H]-	Pulchinenoside a	1.7793454	0.715325526	0.03012859
[M-H]-	Ile-Pro	4.5927369	0.47662056	0.03015823
[M+H-CH5ON3]+	L-hydroxyarginine	0.73581	0.904796165	0.03041465
[M+Na]+	1-hexadecyl-2-azelaoyl-sn-glycero-3-phosphocholine	0.3657839	1.277603648	0.03189461
[M+H]+	Pro-Glu-Lys	0.5235114	0.828146435	0.03260319
[M+H]+	Gln-ser	1.0488426	0.860332162	0.03327733
[M+H]+	Perindopril	2.6134675	0.911907181	0.03344688
[M-H]-	Taurine	2.6173912	1.360266602	0.03388255
[M-H]-	N-formylmethionine	1.1132209	1.116890921	0.03392271
[M+H]+	Asp-Ile-Lys	1.8500407	1.530535318	0.03394831
[2M+H]+	Dihydromethysticin	1.3796732	0.879681815	0.03398227
(M+CH3CN+Na)+	N6-Acetyl-L-lysine	0.806376	0.878840124	0.03399081
[M+H]+	Ala-Ala-Lys	0.3136015	0.96231438	0.03399216
[M+H-C5H9O7P]+	Cytidine 3'-monophosphate	1.752507	0.621817925	0.03417625
[M+Na]+	Hydrocodone	0.4324116	0.883113499	0.03515927
[M-H]-	N-acetyltryptophan	0.410506	0.895892182	0.03545308
[M+H-C5H11NO2]+	6-acetylmorphine	0.5987103	0.63910865	0.03547972
[M-H]-	Thr-Phe	0.188395	0.880004625	0.03548787
[M+H]+	Cys-Gly-Lys	0.3797161	1.454572949	0.03557448
[M-H]-	N-3,4-tridhydroxybenzamide	0.2450583	0.868153548	0.03561082
[M+H]+	4-piperidinecarboxamide	1.358283	1.094216424	0.03573694
[M+H]+	1-pentyl-3-(4-methylnaphthoyl)indole	0.2065024	1.08541848	0.03603237
[2M+H]+	Picrotoxinin	0.3327398	0.816040304	0.03684562
[M+H-H2O]+	.alpha.-propylaminopentiophenone	0.3379484	0.53160857	0.03685052
[M+CH3COOH-H]-	Genistein -7-o-glc-xyl, acetate	0.0918719	0.832047208	0.03717848
(M+H)+	Lys-Leu	0.9460701	0.916341735	0.03742659
[M+H-C2H4O2]+	N-trifluoroacetyl-l-phenylalanine methyl ester	0.7496005	0.55625338	0.03777935
[M-H+2Na]+	Androstan-3-ol-17-one 3-glucuronide	0.5017114	0.917861169	0.03815238
[M-H]-	Plantamajoside	0.4454678	1.130280214	0.03825598
M+	Securinine	0.4257266	1.743012197	0.0387646
[M-H]-	Xylitol	0.788689	0.684851175	0.03884355
[M+H]+	Thaxtomin a	0.2302987	1.115545307	0.0388853
[M-H]-	Cholic acid	0.1929215	0.747995042	0.0391347
[M+H]+	Phe-asp	0.5790743	0.942175986	0.04029304
(M+H)+	2'-O-methylcytidine	1.051686	0.909941033	0.04035852
[M+H]+	Asn-Lys	0.3781357	1.093199708	0.04069245
[M+H]+	Leu-Lys	0.524373	1.062727295	0.04127482
[M+K]+	Laminaritetraose	0.3619738	0.88123611	0.04201529
[M+H]+	Deoxyadenosine	0.5827879	0.890819014	0.04239737
(M+CH3CN+Na)+	Arg-Ile	0.1762651	0.88146214	0.0424602
[M+H]+	Gly-Pro-Lys	0.994885	1.777070821	0.04290498
[M-H]-	4-pyridoxic acid	1.505092	0.754744489	0.04399649
[M+H]+	Val-Pro	1.2331767	1.64676444	0.04407828
[M-H]-	Leu-Thr	2.45559	1.102806431	0.04429558

[M+H]+	Ile-Glu-Lys	1.1905397	1.535218476	0.04431235
[2M-H]-	Phosphonic acid, p-[[4-[(1-oxotetradecyl)amino]phenyl]methyl]-	1.1856685	0.741505928	0.04464549
[M+Na]+	Echinacoside	0.2616807	1.486456904	0.0446541
[M+H]+	2-phenoxyethanol	1.7977516	0.639501013	0.04548082
[M+H-H2O]+	(-)-norepinephrine	2.5029096	2.628041427	0.04552759
[M+Na]+	Cucurbitacin b	0.2055482	1.123401052	0.04598521
[M+H]+	4-hydroxybutanoic acid lactone	0.4659986	0.969900819	0.04606317
(M+H)+	Val-Leu	0.2472613	1.140117694	0.04651671
[M+Na]+	Cer 16:1-d7 (d18:1-d7/16:1)	0.4857123	0.64532746	0.04666107
[M-H]-	L-Arabinono-1,4-lactone	0.3139426	0.898110649	0.04677627
[M+H]+	Val-Gly	0.3705728	0.888939665	0.04716123
[M+H]+	17.beta.-nandrolone decanoate	1.4003382	0.890914452	0.04740964
[M+H]+	L-saccharopine	2.9396358	0.913249478	0.04807491
[M+H]+	Gamma-glutamylvaline	3.6966435	0.47146873	0.04814824
[M+H]+	Lyalosidic acid	0.3785215	1.365214343	0.04855001
[2M+H]+	Isoquinoline n-oxide	0.8248304	0.789406133	0.04857019
[M+NH4]+	Utilin	0.4563107	1.202727686	0.04887681
[M+H]+	Thr-Lys-Lys	0.4013281	1.419344153	0.04931246
[M+H]+	Asparagine	1.9081608	0.940791891	0.04964302
(M-H)-	Nname,cis-9,10-Epoxystearic acid	0.2610937	0.697256425	0.0496899
[2M-H]-	Trans-epoxysuccinyl-l-leucylamido(4-guanidino)butane	0.3048707	1.351269491	0.04973787

Supplementary Table 3 Microbial abundance of oral biofilms

Genus	HB	PB	MB	MBI	p-value	FDR
Abiotrophia	0.055067	0.021029	0.019292	0.011265	0.01951	0.04826
Acetatifactor	0.001607	0.000366	0.008058	0.000352	0.01181	0.03679
Acetobacter	0	0	0	0.002115	0.39163	0.45761
Acidiferrimicrobium	0.003717	0.002175	0.003271	0	0.05458	0.10866
Acidiphilium	0.002831	0.000363	0	0	0.01403	0.04132
Acidisoma	0.002038	0.000725	0.001093	0	0.15113	0.22564
Acidocella	0.001393	0.00145	0.002204	0	0.17446	0.25343
Acinetobacter	0.00035	0.004367	0.000743	0.023079	0.00306	0.01645
Actinomyces	0.13969	0.032338	0.118317	0.032613	0.04931	0.10293
Actinotalea	0.003347	0	0	0	0.00035	0.01645
Adlercreutzia	0.003043	0.000373	0	0.002179	0.04163	0.09134
Agathobacter	0.000321	0.007317	0.002587	0.032994	0.00188	0.01645
Agathobaculum	0	0	0	0.002546	0.00286	0.01645
Aggregatibacter	0.038498	0.078236	0.016453	0.138626	0.00172	0.01645
Agrobacterium	0	0.001119	0	0.002517	0.25441	0.33974
Akkermansia	0.005159	0.007293	0.003668	0.053212	0.01075	0.03449
Alistipes	0.001048	0.001454	0.015049	0.002521	0.00774	0.03005
Allobranchiibius	0.000359	0.001813	0	0.000352	0.41015	0.47157
Alloprevotella	0.03791	0.027347	0.011121	0.042642	0.05352	0.10754
Aminipila	0.000319	0.001088	0.003292	0.00072	0.01877	0.04748
Anaerobutyricum	0.00035	0.001843	0	0.004337	0.05076	0.10314
Anaerococcus	0	0.001836	0	0	0.09722	0.15483
Anaerostipes	0	0.00256	0	0.004016	0.04908	0.10293
Anaerotignum	0	0	0	0.001083	0.01873	0.04748
Arachnia	0.003908	0.001456	0.001824	0.002169	0.89433	0.90273
Arcticibacter	0.00035	0	0.000369	0	0.54873	0.58116
Asticcacaulis	0.000686	0.000725	0.000735	0	0.45346	0.5097
Bacillus	7.00E-04	0	0	0	0.39163	0.45761
Bacteroides	0	0.006203	0.00737	0.022825	0.00404	0.01928
Barnesiella	0.001972	0	0.000355	0.001449	0.45518	0.5097
Bifidobacterium	0.004648	0.001459	0.004025	0.010146	0.1586	0.23517
Blautia	0.001418	0.008017	0.000372	0.02094	0.00605	0.02549
Bradyrhizobium	0.00035	0	0	0.002523	0.00783	0.03005
Brevundimonas	0.000359	0.001464	0	0.005742	0.00347	0.01697
Buchnera	0	0	0	0.004348	0.39163	0.45761
Butyrivibrio	0.000321	0.000721	0	0.00108	0.3664	0.45761
Caballeronia	0	0.113894	0	0.391765	0.00136	0.01645
Campylobacter	1.074185	0.115068	0.080015	0.369722	0.01039	0.03439
Capnocytophaga	0.017532	0.113695	0.006626	0.286291	0.00233	0.01645
Cardiobacterium	0.00271	0.002539	0.001093	0.001443	0.40458	0.47019
Catonella	0.023214	0.01273	0.029632	0.014082	0.06111	0.11944
Chelonobacter	0	0.000746	0	0	0.39163	0.45761
Chryseobacterium	0	0.003694	0	0.005469	0.0183	0.04748
Clostridiumsensustricto	0.001315	0.000366	0.001071	0.002172	0.132	0.20272

Collinsella	0	0.000746	0	0.003622	0.00238	0.01645
Comamonas	0.00035	0.001477	0.000372	0.001788	0.43515	0.49765
Conexibacter	0.002024	0.000363	0.004766	0	0.00186	0.01645
Coprococcus	0	0.000724	0	0.002182	0.01314	0.03979
Corynebacterium	0.043196	0.017135	0.021269	0.026109	0.6075	0.64026
Cryptobacterium	0.001777	0.000363	0.002216	0.000367	0.47638	0.52689
Cutibacterium	0	0.00801	0	0.019141	0.00104	0.01645
Deinococcus	0	0.000735	0	0.002521	0.06768	0.12878
Desulfovibrio	0.000988	0.004393	0.00364	0.019186	0.00321	0.01651
Devosia	0.000686	0.000724	0	0	0.28389	0.37349
Dialister	0	0.004358	0.00181	0.010549	0.03037	0.0702
Diaphorobacter	0.000321	0.000362	0	0.001097	0.41015	0.47157
Dietzia	0.000643	0	0	0.001088	0.25441	0.33974
Dolosigranulum	0	0.002582	0	0.000363	0.0642	0.12324
Dorea	0.000729	0.000731	0.001838	0.004342	0.15977	0.23527
Duncaniella	0.00596	0.001812	0.016815	0	0.00873	0.03225
Dysosmobacter	0	0	0.000731	0	0.09722	0.15483
Eggerthia	0.000319	0	0.001104	0.000364	0.45518	0.5097
Eikenella	0.104622	0.009066	0.00182	0.011961	0.00137	0.01645
Elizabethkingia	0.00035	0.008426	0.000355	0	0.11108	0.17561
Enterocloster	0	0	0	0.005457	0.00035	0.01645
Enterococcus	0	0.000739	0	0.022182	0.00206	0.01645
Entomoplasma	0	0	0	0.002542	0.39163	0.45761
Escherichia	0.000359	0.010117	0.004712	0.037471	0.01022	0.03439
Eubacterium	0.020643	0.005094	0.01172	0.007201	0.06049	0.11932
Faecalibacillus	0	0.000731	0	0.002157	0.07899	0.14461
Faecalibacterium	0	0.025571	0	0.084143	0.00108	0.01645
Faecalibaculum	0.005182	0.003986	0.036623	0	0.00238	0.01645
Faecalicatena	0.003505	0.001094	0.005463	0.021766	0.00265	0.01645
Filifactor	0.0065	0.073879	0.021311	0.059672	0.0027	0.01645
Finegoldia	0	0.006978	0	0	0.01873	0.04748
Flavonifractor	0	0	0	0.00108	0.09722	0.15483
Flintibacter	0.001002	0.000724	0.003647	0.001442	0.03804	0.08431
Fluviicola	0	0.001477	0	0	0.09722	0.15483
Fretibacterium	0.001978	0.000358	0.000372	0.000352	0.36611	0.45761
Fusicatenibacter	0.00035	0.000717	0.000355	0.006508	0.00471	0.02156
Fusobacterium	0.084426	37.331214	9.570784	47.393355	0.00145	0.01645
Gemella	4.02714	2.387868	3.58678	1.591748	0.00915	0.03225
Gemmiger	0	0.005815	0	0.046065	0.0017	0.01645
Gp10	0	0.000363	0	0	0.39163	0.45761
Granulicatella	1.789127	1.230345	3.144121	0.921698	0.00669	0.02767
Granulicella	0.004487	0.000725	0.006594	0	0.00266	0.01645
Haemophilus	12.453629	14.789384	12.439446	14.693442	0.87522	0.89181
Helicobacter	0.000678	0	0	0	0.09722	0.15483
Holdemanella	0	0.000358	0	0.001447	0.01058	0.03447
Hominimerdicola	0	0.000358	0	0.002917	0.00783	0.03005
Hoylesella	0.923617	1.100808	0.854835	1.337644	0.12543	0.19593

Ihubacter	7.00E-04	0	0	0	0.39163	0.45761
Kineothrix	0.011322	0.015238	0.021156	0.023924	0.47788	0.52689
Kingella	0.000715	0.001094	0.000745	0.000731	0.89113	0.90273
Klebsiella	2.777974	0.177246	0.024958	1.238007	0.26505	0.35177
Kocuria	0	0.000717	0	0.005399	0.00172	0.01645
Kroppenstedtia	0.000669	0	0	0	0.09722	0.15483
Lachnoanaerobaculum	0.092718	0.107237	0.065601	0.100609	0.30874	0.4023
Lachnospira	0	0.013144	0	0.043732	0.00208	0.01645
Lacrimispora	0.008559	0.000729	0.001108	0.00073	0.79968	0.81872
Lacticaseibacillus	0	0	0	0.007229	0.00035	0.01645
Lactobacillus	0.011082	0.005077	0.009184	0.001089	0.02231	0.05389
Lactococcus	0	0.000717	0	0.007304	0.22218	0.30621
Laedolimicola	0.001031	0.00183	0.005504	0.001083	0.02887	0.06747
Lancefieldella	0.079984	0.041786	0.108883	0.009782	0.0096	0.03327
Lautropia	0.008783	0.004731	0.008403	0.006522	0.76536	0.78983
Lawsonibacter	0	0.000724	0.00109	0.001093	0.20504	0.28813
Leifsonia	0.000359	0	0.000373	0	0.54873	0.58116
Lentihominibacter	0.000964	0.000363	0	0	0.54873	0.58116
Leptotrichia	0.348488	0.173034	0.276162	0.188244	0.14192	0.21338
Lichenicoccus	0.002084	0.002901	0	0	0.09592	0.15483
Lichenicola	0.002831	0.002537	0	0	0.03404	0.07624
Ligilactobacillus	0.003387	0.00254	0.005487	0.004343	0.47052	0.52416
Limosilactobacillus	0.001386	0	0.000361	0.000726	0.22581	0.30923
Longibaculum	0.001636	0	0	0	0.09722	0.15483
Longicatena	0	0.000366	0	0.00073	0.25441	0.33974
Mammaliicoccus	0.000964	0.000363	0.000372	0	0.76778	0.78983
Marisediminicola	0.005484	0.002536	0.000372	0.000363	0.01575	0.04442
Massilia	0.000669	0.001094	0	0.002544	0.12576	0.19593
Megamonas	0	0.009303	0	0.012837	0.01539	0.04442
Megasphaera	0	0	0.000361	0.009692	0.00783	0.03005
Metamycoplasma	0.001022	0.000717	0.000372	0.000726	0.93098	0.93533
Methylobacterium	0.000709	0.00293	0	0.005787	0.00339	0.01693
Methylovirgula	0.00176	0.000725	0	0	0.07899	0.14461
Microbacterium	0.001668	0.000373	0	0.001089	0.12868	0.19904
Micrococcus	0	0.001463	0	0.012598	0.00117	0.01645
Mobiluncus	0	0.000362	0.00071	0.000716	0.54276	0.58116
Mogibacterium	0	0.000731	0.000733	0.000362	0.43781	0.49803
Moraxella	0.00035	0.032477	0	0.031507	0.01371	0.04094
Muribaculum	0.003	0.001812	0.004748	0.000352	0.04393	0.0954
Negativibacillus	0	0	0	0.001459	0.09722	0.15483
Negativicoccus	0	0.00692	0	0	0.01873	0.04748
Neglecta	0	0.000366	0.001838	0.002546	0.02697	0.06372
Neisseria	0.2787	0.482613	0.191099	0.755348	0.00298	0.01645
Odoribacter	0	0	0.001118	0	0.09722	0.15483
Olsenella	0.017308	0.003983	0.005525	0.001461	0.07937	0.14461
Oribacterium	0.185869	0.158863	0.162581	0.26911	0.16203	0.23698
Oscillibacter	0.00035	0.000358	0.00145	0	0.13528	0.20482

Paenibacillus	0	0	0	0.008078	0.39163	0.45761
Paeniclostridium	0.000643	0	0	0.001451	0.22218	0.30621
Parabacteroides	0	0.001109	0.002148	0.005014	0.0323	0.07387
Paraburkholderia	0	0	0	0.003268	0.39163	0.45761
Paracoccus	0	0.000366	0	0.010866	0.00085	0.01645
Paramuribaculum	0.001324	0.001083	0.004071	0.00073	0.37987	0.45761
Paraprevotella	0	0.001441	0	0.000367	0.25441	0.33974
Parasutterella	0.004467	0.002179	0.002545	0.000367	0.07669	0.14461
Parvimonas	0.547483	0.819986	0.907162	0.983231	0.38811	0.45761
Pedobacter	0.000359	0.000363	0	0	0.54873	0.58116
Peptidiphaga	0.003828	0	0.001825	0	0.19704	0.27871
Peptococcus	0.000321	0.00109	0	0.000734	0.4953	0.54331
Peptoniphilus	0	0.003248	0	0	0.01873	0.04748
Peptostreptococcaceae_incertae_sedis	0.016067	0.002546	0.003657	0.004684	0.32051	0.41512
Peptostreptococcus	0.248271	0.034888	0.103426	0.023885	0.0029	0.01645
Perlabentimonas	0.001631	0	0	0.000728	0.09508	0.15483
Phascolarctobacterium	0	0.008433	0	0.020701	0.00301	0.01645
Phenylobacterium	0.00099	0.000363	0.000741	0	0.2849	0.37349
Phocaeicola	0	0.038112	0	0.071168	0.00076	0.01645
Polaromonas	0.001976	0	0	0	0.09722	0.15483
Porphyromonas	0.973566	2.301938	3.22862	2.667737	0.05085	0.10314
Prevotella	0.585685	8.310556	1.46742	4.364835	0.00323	0.01651
Prevotellamassilia	0.001281	0	0.015462	0	0.00117	0.01645
Propionibacterium	0.000638	0.000358	0.000372	0	0.76778	0.78983
Providencia	0	0.002509	0	0.006149	0.07899	0.14461
Pseudoflavonifractor	0	0	0.001107	0	0.39163	0.45761
Pseudoleptotrichia	0	0	0	0.001092	0.09722	0.15483
Pseudomonas	0.00035	0.001094	0	0.005794	0.00184	0.01645
Ralstonia	0	0	0	0.001078	0.01873	0.04748
Rhodococcus	0	0	0	0.000725	0.39163	0.45761
Rickettsia	0	0.005105	0	0.007526	0.02276	0.05437
Rickettsiella	0	0	0	0.016704	0.39163	0.45761
Rodentibacter	0.002478	0.001814	0.001865	0.001819	0.97641	0.97641
Romboutsia	0.002383	0.002551	0.011665	0.001077	0.00911	0.03225
Roseateles	0	0	0	0.003637	0.00286	0.01645
Roseburia	0.001685	0.006602	0.001115	0.019198	0.02007	0.04904
Roseomonas	0	0.000373	0	0.003969	0.00122	0.01645
Rothia	3.916397	0.723214	3.39915	0.325146	0.01101	0.0348
Ructibacterium	0	0.000362	0	0.001079	0.0642	0.12324
Ruminococcoides	0	0.001485	0	0.001812	0.03404	0.07624
Ruminococcus	0.001607	0.001087	0.000372	0.00182	0.50551	0.5517
SR1	0.004594	0.009855	0.008	0.02433	0.01223	0.03757
Saccharibacteria	0.050104	0.117139	0.096741	0.287405	0.00081	0.01645
Scardovia	0.001355	0	0	0	0.00286	0.01645
Schaalia	9.511373	4.174311	5.533503	4.657395	0.7407	0.77306
Schaedlerella	0.001361	0.002557	0.002944	0.011592	0.0104	0.03439
Schwartzia	0.00064	0	0	0	0.09722	0.15483

Segatella	0.040001	0.189896	0.022114	0.200503	0.01591	0.04442
Selenomonas	0.026584	0.002192	0.003669	0.002887	0.01552	0.04442
Seramator	0	0.001119	0	0	0.39163	0.45761
Serratia	0.000359	0	0	0.012335	0.22218	0.30621
Shuttleworthia	0.002007	0.000725	0.001833	0	0.09532	0.15483
Skermanella	0	0	0	0.00073	0.09722	0.15483
Slackia	5.118735	6.86096	22.304078	0.030238	0.00891	0.03225
Sodalis	0	0.002904	0	0.00366	0.1951	0.2778
Solibaculum	0.000686	0.000724	0.001451	0.01195	0.0057	0.02452
Solirubrobacter	0	0.000363	0	0	0.39163	0.45761
Solobacterium	0.154622	0.1562	0.252094	0.236887	0.13311	0.20297
Sphingomonas	0.002061	0.006552	0	0.005047	0.00266	0.01645
Spodiobacter	0.001429	0.002191	0	0.009083	0.00501	0.02246
Staphylococcus	0.000365	0.060992	0.000361	0.013065	0.00873	0.03225
Stenotrophomonas	0.003485	0.001091	0.001831	0	0.0876	0.15483
Stomatobaculum	1.966703	0.223871	0.24648	0.081725	0.00127	0.01645
Streptococcus	45.798764	9.903098	23.501298	11.811783	0.00147	0.01645
Succinivibrio	0	0	0	0.003263	0.00035	0.01645
Sutterella	0	0.001434	0	0.003606	0.00435	0.02033
Tannerella	0.002713	0.004367	0.001847	0.003644	0.68167	0.71492
Thermus	0	0	0	0.001431	0.01873	0.04748
Trebonia	0.001088	0.001813	0	0	0.1951	0.2778
Treponema	0.003152	0	0	0.001443	0.04908	0.10293
Tsukamurella	0.000638	0	0	0.000728	0.54873	0.58116
Turicibacter	0.001031	0	0.00221	0	0.0454	0.0976
Unclassified	0.750024	1.47981	0.416837	2.904763	0.0006	0.01645
Undibacter	0.000678	0.001087	0	0	0.1894	0.27329
Veillonella	5.471772	5.638974	7.465722	0.50582	0.05085	0.10314
Vescimonas	0.000319	0.002937	0.000372	0.014134	0.0053	0.02324

**Species**	**HB**	**PB**	**MB**	**MBI**	**p.value**	**FDR**
Abiotrophia_defectiva	0.055067	0.021029	0.019292	0.011265	0.01951	0.05096
Acetatifactor_muris	0.001607	0.000366	0.008058	0.000352	0.01181	0.0399
Acetobacter_pasteurianus	0	0	0	0.002115	0.39163	0.45468
Acidiferrimicrobium_australe	0.003717	0.002175	0.003271	0	0.05458	0.11504
Acidiphilium_angustum	0.002831	0.000363	0	0	0.01403	0.04271
Acidisoma_tundrae	0.002038	0.000725	0.001093	0	0.15113	0.22878
Acidocella_aquatica	0.001393	0.00145	0.002204	0	0.17446	0.25979
Acinetobacter_calcoaceticus	0	0.000373	0	0.00869	0.00085	0.01908
Acinetobacter_haemolyticus	0	0.00109	0	0	0.09722	0.15221
Acinetobacter_johnsonii	0.00035	0.002546	0.000372	0.004949	0.33097	0.42979
Acinetobacter_junii	0	0.000358	0.000372	0.00762	0.00177	0.01908
Acinetobacter_rudis	0	0	0	0.001821	0.09722	0.15221
Actinomyces_dentalis	0.002334	0.000358	0.001104	0.001814	0.30667	0.40205
Actinomyces_graevenitzii	0.015649	0.004009	0.009172	0.002181	0.04426	0.0994
Actinomyces_massiliensis	0.016124	0.003246	0.013573	0.001824	0.25485	0.3423
Actinomyces_oris	0.101197	0.023261	0.091896	0.024971	0.07955	0.14828
Actinomyces_timonensis	0.003031	0.000363	0.000727	0	0.02304	0.05845

Actinotalea_fermentans	0.003347	0	0	0	0.00035	0.01896
Adlercreutzia_equolifaciens	0.000643	0	0	0.000362	0.54873	0.57606
Adlercreutzia_mucosicola	0.001763	0.000373	0	0.001816	0.14941	0.22743
Agathobacter_rectalis	0.000321	0.007317	0.002587	0.032994	0.00188	0.01908
Aggregatibacter_aphrophilus	0.016712	0.006555	0.002168	0.010135	0.0246	0.06127
Aggregatibacter_segnis	0.021785	0.071681	0.014285	0.128491	0.00196	0.01908
Akkermansia_muciniphila	0.005159	0.007293	0.003668	0.053212	0.01075	0.03682
Alistipes_finegoldii	0	0.000366	0.007676	0.001089	0.00233	0.0192
Alistipes_shahii	0	0	0.002209	0.000352	0.00783	0.03201
Allobranchiibius_huperziae	0.000359	0.001813	0	0.000352	0.41015	0.47022
Alloprevotella_rava	0.037267	0.02188	0.010008	0.037235	0.0643	0.12495
Alloprevotella_tannerae	0.000643	0.005467	0.001114	0.005407	0.00526	0.0262
Anaerobutyricum_soehngenii	0.00035	0.001843	0	0.004337	0.05076	0.10782
Anaerococcus_octavius	0	0.001836	0	0	0.09722	0.15221
Anaerostipes_hadrus	0	0.00256	0	0.004016	0.04908	0.10506
Anaerotignum_faecicola	0	0	0	0.001083	0.01873	0.04983
Arachnia_rubra	0.003908	0.001456	0.001824	0.002169	0.89433	0.89924
Arcticibacter_eurypsychrophilus	0.00035	0	0.000369	0	0.54873	0.57606
Asticcacaulis_solisilvae	0.000686	0.000725	0.000735	0	0.45346	0.50698
Bacillus_amyloliquefaciens	7.00E-04	0	0	0	0.39163	0.45468
Bacteroides_caccae	0	0.000746	0	0.004722	0.01403	0.04271
Bacteroides_rodentium	0	0	0.00737	0	0.00035	0.01896
Bacteroides_salyersiae	0	0	0	0.001092	0.09722	0.15221
Bacteroides_stercoris	0	0.003635	0	0.010884	0.01221	0.0399
Bacteroides_thetaiotaomicron	0	0.001821	0	0.006127	0.00108	0.01908
Barnesiella_intestinihominis	0.001972	0	0.000355	0.001449	0.45518	0.50698
Bifidobacterium_animalis	0.003051	0.000362	0.004025	0	0.03318	0.07771
Bifidobacterium_catenulatum	0.000321	0.001097	0	0.007962	0.00347	0.02209
Bifidobacterium_longum	0.001275	0	0	0.002185	0.09508	0.15221
Blautia_hominis	0	0.002926	0	0.004311	0.0454	0.09951
Bradyrhizobium_denitrificans	0.00035	0	0	0.002523	0.00783	0.03201
Brevundimonas_vesicularis	0	0.000739	0	0.005742	0.00206	0.01908
Buchnera_aphidicola	0	0	0	0.004348	0.39163	0.45468
Butyrivibrio_proteoclasticus	0.000321	0.000721	0	0.00108	0.3664	0.45468
Caballeronia_mineralivorans	0	0.036637	0	0.148444	0.0017	0.01908
Campylobacter_massiliensis	0.935969	0.088548	0.043139	0.319528	0.02546	0.06284
Capnocytophaga_granulosa	0.005248	0.090043	0.002954	0.219926	0.00178	0.01908
Capnocytophaga_leadbetteri	0.007238	0.019692	0.001832	0.05661	0.01061	0.03679
Capnocytophaga_ochracea	0.003385	0	0.000743	0.002889	0.028	0.06789
Capnocytophaga_sputigena	0.001662	0.001811	0.000723	0.005413	0.18665	0.27644
Cardiobacterium_hominis	0.00271	0.002539	0.001093	0.001443	0.40458	0.46775
Catonella_morbi	0.023214	0.01273	0.029632	0.014082	0.06111	0.12312
Chelonobacter_oris	0	0.000746	0	0	0.39163	0.45468
Chryseobacterium_taklimakanense	0	0	0	0.001089	0.39163	0.45468
Collinsella_aerofaciens	0	0.000746	0	0.003622	0.00238	0.0192
Comamonas_testosteroni	0.00035	0.001477	0.000372	0.001788	0.43515	0.49474
Conexibacter_arvalis	0.000715	0.000363	0.002189	0	0.03665	0.08369

Coprococcus_ammoniilyticus	0	0.000724	0	0.002182	0.01314	0.04187
Corynebacterium_argentoratense	0.0058	0.000358	0.002565	0	0.09384	0.15221
Corynebacterium_casei	0.007744	0.000725	0.000733	0.000731	0.95573	0.95573
Corynebacterium_durum	0.028691	0.006541	0.017601	0.005794	0.02825	0.06789
Corynebacterium_matruchotii	0.000962	0.001083	0.000369	0.001082	0.76331	0.78206
Corynebacterium_pseudodiphtheriticum	0	0.004016	0	0.001825	0.0454	0.09951
Corynebacterium_tuberculostearicum	0	0.002933	0	0.009815	0.00208	0.01908
Cryptobacterium_curtum	0.001777	0.000363	0.002216	0.000367	0.47638	0.52586
Cutibacterium_acnes	0	0.00801	0	0.019141	0.00104	0.01908
Deinococcus_antarcticus	0	0	0	0.002169	0.01873	0.04983
Desulfovibrio_piger	0	0	0.000369	0.00108	0.25441	0.3423
Desulfovibrio_porci	0.000988	0.004393	0.003271	0.018106	0.00402	0.02392
Devosia_insulae	0.000686	0.000724	0	0	0.28389	0.37397
Dialister_hominis	0	0.000731	0	0.004734	0.05933	0.12131
Dialister_invisus	0	0.003261	0.00181	0.003991	0.33946	0.43873
Dialister_pneumosintes	0	0	0	0.000362	0.39163	0.45468
Dialister_succinatiphilus	0	0.000366	0	0.000727	0.28389	0.37397
Diaphorobacter_nitroreducens	0.000321	0.000362	0	0.001097	0.41015	0.47022
Dietzia_lutea	0.000643	0	0	0.001088	0.25441	0.3423
Dolosigranulum_pigrum	0	0.002582	0	0.000363	0.0642	0.12495
Dorea_formicigenerans	0.000365	0.000731	0	0.001813	0.45518	0.50698
Duncaniella_dubosii	0.002248	0.001087	0.006579	0	0.05613	0.1174
Duncaniella_muris	0.00275	0.000363	0.00696	0	0.00572	0.02701
Dysosmobacter_welbionis	0	0	0.000369	0	0.39163	0.45468
Eggerthia_catenaformis	0.000319	0	0.001104	0.000364	0.45518	0.50698
Eikenella_corrodens	0.104622	0.009066	0.00182	0.011961	0.00137	0.01908
Enterocloster_bolteae	0	0	0	0.005457	0.00035	0.01896
Enterococcus_faecalis	0	0.000739	0	0.022182	0.00206	0.01908
Entomoplasma_freundtii	0	0	0	0.002542	0.39163	0.45468
Eubacterium_sulci	0.020643	0.005094	0.01172	0.007201	0.06049	0.12278
Faecalibacillus_intestinalis	0	0.000731	0	0.002157	0.07899	0.14825
Faecalibacterium_prausnitzii	0	0.025571	0	0.084143	0.00108	0.01908
Faecalibaculum_rodentium	0.005182	0.003986	0.036623	0	0.00238	0.0192
Faecalicatena_orotica	0.002119	0.000358	0.002538	0.001787	0.15326	0.23074
Filifactor_alocis	0.0065	0.073879	0.021311	0.059672	0.0027	0.01984
Finegoldia_magna	0	0.006978	0	0	0.01873	0.04983
Flavonifractor_plautii	0	0	0	0.00108	0.09722	0.15221
Flintibacter_butyricus	0.001002	0.000724	0.003647	0.001442	0.03804	0.08613
Fluviicola_hefeinensis	0	0.001477	0	0	0.09722	0.15221
Fretibacterium_fastidiosum	0.001978	0.000358	0.000372	0.000352	0.36611	0.45468
Fusicatenibacter_saccharivorans	0.00035	0.000717	0.000355	0.006508	0.00471	0.02583
Fusobacterium_nucleatum	0.084426	37.322027	9.570784	47.371926	0.00145	0.01908
Gemella_morbillorum	4.02714	2.387868	3.58678	1.591748	0.00915	0.03388
Gemmiger_formicilis	0	0.005815	0	0.046065	0.0017	0.01908
Granulicatella_adiacens	1.645631	1.133876	3.092799	0.763437	0.00901	0.03388
Granulicatella_elegans	0.143497	0.09647	0.051322	0.158261	0.00615	0.02762
Granulicella_cerasi	0.001723	0	0.000372	0	0.01825	0.04983

Haemophilus_haemolyticus	0.000365	0.000717	0	0.011214	0.00177	0.01908
Haemophilus_parainfluenzae	12.453265	14.788667	12.439446	14.682228	0.87522	0.88818
Holdemanella_porci	0	0.000358	0	0.001447	0.01058	0.03679
Hominimerdicola_aceti	0	0.000358	0	0.002917	0.00783	0.03201
Hoylesella_loescheii	0.001607	0.011293	0.000735	0.03581	0.00166	0.01908
Hoylesella_nanceiensis	0.595384	0.781238	0.738567	0.877945	0.47169	0.52325
Hoylesella_oralis	0	0.001087	0	0	0.09722	0.15221
Hoylesella_pleuritidis	0	0.000363	0	0.000362	0.54873	0.57606
Hoylesella_shahii	0.326626	0.306827	0.115534	0.423526	0.01223	0.0399
Ihubacter_massiliensis	7.00E-04	0	0	0	0.39163	0.45468
Kineothrix_alysoides	0.011322	0.015238	0.021156	0.023924	0.47788	0.52586
Kingella_oralis	0.000715	0.001094	0.000745	0.000731	0.89113	0.89924
Klebsiella_pneumoniae	2.777974	0.177246	0.024958	1.238007	0.26505	0.35427
Kocuria_palustris	0	0.000717	0	0.005399	0.00172	0.01908
Kroppenstedtia_eburnea	0.000669	0	0	0	0.09722	0.15221
Lachnoanaerobaculum_umeaense	0.079924	0.060712	0.058613	0.037284	0.57359	0.59987
Lachnospira_eligens	0	0.003335	0	0.011296	0.01221	0.0399
Lacticaseibacillus_rhamnosus	0	0	0	0.007229	0.00035	0.01896
Lactobacillus_johnsonii	0.011082	0.005077	0.009184	0.001089	0.02231	0.05713
Lactococcus_cremoris	0	0.000717	0	0.007304	0.22218	0.30902
Laedolimicola_ammoniilytica	0.001031	0.00183	0.005504	0.001083	0.02887	0.06878
Lancefieldella_parvula	0.079984	0.041786	0.108883	0.009782	0.0096	0.03505
Lautropia_mirabilis	0.008783	0.004731	0.008403	0.006522	0.76536	0.78206
Lawsonibacter_hominis	0	0.000724	0.00109	0.001093	0.20504	0.29261
Leifsonia_aquatica	0.000359	0	0.000373	0	0.54873	0.57606
Lentihominibacter_hominis	0.000964	0.000363	0	0	0.54873	0.57606
Leptotrichia_hongkongensis	0	0.000358	0	0.001088	0.22218	0.30902
Leptotrichia_wadei	0.000683	0	0.000373	0.001445	0.10194	0.15781
Lichenicoccus_roseus	0.002084	0.002901	0	0	0.09592	0.15221
Lichenicola_cladoniae	0.002831	0.002537	0	0	0.03404	0.07838
Limosilactobacillus_reuteri	0.001386	0	0.000361	0.000726	0.22581	0.31248
Longibaculum_muris	0.001636	0	0	0	0.09722	0.15221
Longicatena_caecimuris	0	0.000366	0	0.00073	0.25441	0.3423
Mammaliicoccus_sciuri	0.000964	0.000363	0.000372	0	0.76778	0.78206
Marisediminicola_antarctica	0.005484	0.002536	0.000372	0.000363	0.01575	0.0469
Megamonas_funiformis	0	0.009303	0	0.012837	0.01539	0.04635
Megasphaera_elsdenii	0	0	0.000361	0.009692	0.00783	0.03201
Metamycoplasma_orale	0.001022	0.000717	0.000372	0.000363	0.89596	0.89924
Metamycoplasma_salivarium	0	0	0	0.000363	0.39163	0.45468
Methylobacterium_aquaticum	0	0.001829	0	0.000731	0.06768	0.1306
Methylobacterium_bullatum	0.000709	0.000373	0	0.001444	0.21588	0.3049
Methylovirgula_ligni	0.00176	0.000725	0	0	0.07899	0.14825
Micrococcus_aloeverae	0	0.001463	0	0.012598	0.00117	0.01908
Mobiluncus_curtisii	0	0.000362	0.00071	0.000716	0.54276	0.57606
Mogibacterium_pumilum	0	0.000731	0.000733	0.000362	0.43781	0.4957
Moraxella_catarrhalis	0	0.020788	0	0	0.01873	0.04983
Moraxella_osloensis	0.00035	0.011689	0	0.031507	0.0023	0.0192

Muribaculum_gordoncarteri	0.000319	0	0	0	0.39163	0.45468
Muribaculum_intestinale	0.001688	0.00145	0.004377	0.000352	0.05904	0.12131
Negativibacillus_massiliensis	0	0	0	0.001459	0.09722	0.15221
Negativicoccus_succinicivorans	0	0.00692	0	0	0.01873	0.04983
Neglecta_timonensis	0	0.000366	0.001838	0.002546	0.02697	0.06598
Neisseria_bacilliformis	0	0	0	0.00072	0.09722	0.15221
Neisseria_elongata	0.026414	0.086941	0.022985	0.188152	0.00466	0.02583
Neisseria_oralis	0	0.000724	0.000372	0.004693	0.00609	0.02762
Odoribacter_laneus	0	0	0.001118	0	0.09722	0.15221
Oribacterium_sinus	0.185869	0.158863	0.162581	0.26911	0.16203	0.2426
Oscillibacter_valericigenes	0.00035	0.000358	0.00145	0	0.13528	0.20707
Paenibacillus_etheri	0	0	0	0.008078	0.39163	0.45468
Paeniclostridium_sordellii	0.000643	0	0	0.001451	0.22218	0.30902
Parabacteroides_distasonis	0	0.000736	0.002148	0.002514	0.05834	0.12109
Parabacteroides_merdae	0	0.000373	0	0.0025	0.0642	0.12495
Paraburkholderia_jirisanensis	0	0	0	0.003268	0.39163	0.45468
Paracoccus_aminophilus	0	0.000366	0	0.010866	0.00085	0.01908
Paramuribaculum_intestinale	0.001324	0.001083	0.004071	0.00073	0.37987	0.45468
Paraprevotella_clara	0	0.001441	0	0.000367	0.25441	0.3423
Parasutterella_excrementihominis	0.004467	0.002179	0.002545	0.000367	0.07669	0.14592
Parvimonas_micra	0.547483	0.819986	0.907162	0.983231	0.38811	0.45468
Peptidiphaga_gingivicola	0.003828	0	0.001825	0	0.19704	0.28267
Peptococcus_simiae	0.000321	0.00109	0	0.000734	0.4953	0.54068
Peptoniphilus_lacydonensis	0	0.003248	0	0	0.01873	0.04983
Peptostreptococcus_stomatis	0.248271	0.034888	0.103426	0.023885	0.0029	0.01984
Perlabentimonas_gracilis	0.001631	0	0	0.000728	0.09508	0.15221
Phascolarctobacterium_faecium	0	0.007687	0	0.018893	0.00355	0.02209
Phascolarctobacterium_succinatutens	0	0.000746	0	0.001808	0.02328	0.05852
Phocaeicola_coprocola	0	0.001094	0	0.002189	0.10016	0.15593
Phocaeicola_massiliensis	0	0.002589	0	0.009803	0.01604	0.04727
Phocaeicola_plebeius	0	0.008734	0	0.009765	0.00654	0.02889
Phocaeicola_sartorii	0	0	0	0.003267	0.09722	0.15221
Phocaeicola_vulgatus	0	0.025696	0	0.046145	0.00087	0.01908
Polaromonas_naphthalenivorans	0.001976	0	0	0	0.09722	0.15221
Porphyromonas_catoniae	0.041201	0.021864	0.00111	0.038734	0.01263	0.04072
Porphyromonas_endodontalis	0.828921	0.3163	0.179875	0.206329	0.04782	0.10316
Porphyromonas_gingivalis	0.00248	1.139378	2.988691	0.751355	0.00545	0.02664
Porphyromonas_pasteri	0.100964	0.823657	0.058945	1.67132	0.00253	0.01984
Porphyromonas_somerae	0	0.000739	0	0	0.09722	0.15221
Prevotella_aurantiaca	0.052501	0.22606	0.09545	0.139787	0.02035	0.05259
Prevotella_denticola	0.001309	0.000358	0.000372	0	0.22957	0.31608
Prevotella_intermedia	0.001657	0.005516	0	0.009773	0.00519	0.0262
Prevotella_melaninogenica	0.471371	7.717797	1.23583	3.954097	0.00393	0.02392
Prevotella_micans	0	0.000363	0.000373	0	0.54873	0.57606
Prevotella_nigrescens	0.000319	0.003248	0	0.003985	0.00497	0.02592
Prevotella_pallens	0.032828	0.071199	0.072158	0.047223	0.31452	0.41037
Prevotella_veroralis	0.025699	0.23766	0.063237	0.16473	0.00273	0.01984

Prevotellamassilia_timonensis	0.001281	0	0.015462	0	0.00117	0.01908
Propionibacterium_acidifaciens	0.000638	0.000358	0.000372	0	0.76778	0.78206
Pseudoleptotrichia_goodfellowii	0	0	0	0.001092	0.09722	0.15221
Pseudomonas_aeruginosa	0.00035	0.000735	0	0.002903	0.00483	0.02592
Pseudomonas_oryzihabitans	0	0.000358	0	0.001441	0.0642	0.12495
Ralstonia_pickettii	0	0	0	0.001078	0.01873	0.04983
Rhodococcus_fascians	0	0	0	0.000725	0.39163	0.45468
Rickettsia_conorii	0	0.005105	0	0.006437	0.03025	0.07146
Rickettsiella_massiliensis	0	0	0	0.016704	0.39163	0.45468
Romboutsia_timonensis	0.002383	0.002551	0.011665	0.001077	0.00911	0.03388
Roseateles_noduli	0	0	0	0.003275	0.00286	0.01984
Roseateles_saccharophilus	0	0	0	0.000362	0.39163	0.45468
Roseburia_faecis	0.001685	0.000363	0.001115	0	0.4953	0.54068
Roseburia_inulinivorans	0	0.006239	0	0.019198	0.00355	0.02209
Roseomonas_gilardii	0	0.000373	0	0.003969	0.00122	0.01908
Rothia_dentocariosa	0.948754	0.098601	0.364882	0.066569	0.00596	0.02762
Rothia_mucilaginosa	2.967643	0.624613	3.034268	0.258576	0.01395	0.04271
Ructibacterium_gallinarum	0	0.000362	0	0.001079	0.0642	0.12495
Ruminococcoides_bili	0	0.001485	0	0.001812	0.03404	0.07838
Ruminococcus_callidus	0	0.000724	0	0.001453	0.1894	0.27751
Ruminococcus_champanellensis	0.001607	0.000363	0.000372	0	0.76778	0.78206
Ruminococcus_flavefaciens	0	0	0	0.000367	0.39163	0.45468
Scardovia_wiggsiae	0.001355	0	0	0	0.00286	0.01984
Schaalia_odontolytica	9.490902	4.15313	5.506906	4.629702	0.7407	0.76876
Schaedlerella_arabinosiphila	0.001361	0.002557	0.002944	0.011592	0.0104	0.03679
Schwartzia_succinivorans	0.00064	0	0	0	0.09722	0.15221
Segatella_buccae	0.001777	0	0	0	0.09722	0.15221
Segatella_copri	0	0.084564	0.000373	0.053927	0.01371	0.04271
Segatella_oris	0.005226	0.001101	0.001106	0.002907	0.42386	0.48391
Segatella_oulorum	0.019651	0.000362	0.002954	0.00109	0.21423	0.30414
Segatella_salivae	0.013026	0.095803	0.015844	0.130247	0.01055	0.03679
Selenomonas_artemidis	0.00064	0	0	0.000362	0.28389	0.37397
Selenomonas_flueggei	0.001967	0	0	0	0.01873	0.04983
Selenomonas_noxia	0.010727	0	0.000373	0	0.00783	0.03201
Selenomonas_sputigena	0.013249	0.002192	0.003296	0.002524	0.07369	0.1412
Seramator_thermalis	0	0.001119	0	0	0.39163	0.45468
Serratia_marcescens	0	0	0	0.010885	0.09722	0.15221
Serratia_symbiotica	0.000359	0	0	0.001449	0.54873	0.57606
Shuttleworthia_satelles	0.002007	0.000725	0.001833	0	0.09532	0.15221
Skermanella_aerolata	0	0	0	0.00073	0.09722	0.15221
Slackia_exigua	5.118735	6.86096	22.304078	0.030238	0.00891	0.03388
Sodalis_praecaptivus	0	0.002904	0	0.00366	0.1951	0.28136
Solibaculum_mannosilyticum	0.000686	0.000724	0.001451	0.01195	0.0057	0.02701
Solirubrobacter_ginsenosidimutans	0	0.000363	0	0	0.39163	0.45468
Solobacterium_moorei	0.154622	0.1562	0.252094	0.236887	0.13311	0.2049
Spodiobacter_cordis	0.001429	0.002191	0	0.009083	0.00501	0.02592
Staphylococcus_capitis	0.000365	0.060992	0.000361	0.013065	0.00873	0.03388

Stenotrophomonas_maltophilia	0.003485	0.001091	0.001831	0	0.0876	0.15221
Stomatobaculum_longum	1.966703	0.223871	0.24648	0.081725	0.00127	0.01908
Streptococcus_agalactiae	0.000672	0	0	0	0.09722	0.15221
Streptococcus_anginosus	24.864081	1.597803	4.864566	2.67293	0.00877	0.03388
Streptococcus_constellatus	0.009305	0.001097	0.001854	0.001084	0.52203	0.5676
Streptococcus_mutans	0.010048	0.000725	0.002548	0.001442	0.00802	0.0323
Streptococcus_oralis	16.658611	6.920925	13.865946	8.120238	0.00424	0.02469
Streptococcus_parasanguinis	3.147147	1.213794	4.04912	0.925748	0.00209	0.01908
Succinivibrio_dextrinosolvens	0	0	0	0.003263	0.00035	0.01896
Sutterella_massiliensis	0	0.001434	0	0.003606	0.00435	0.02483
Tannerella_serpentiformis	0.002713	0.004367	0.001847	0.003644	0.68167	0.71018
Thermus_scotoductus	0	0	0	0.001431	0.01873	0.04983
Trebonia_kvetii	0.001088	0.001813	0	0	0.1951	0.28136
Treponema_medium	0.001022	0	0	0.00108	0.1951	0.28136
Treponema_socranskii	0.00213	0	0	0	0.09722	0.15221
Tsukamurella_pseudospumae	0.000638	0	0	0.000728	0.54873	0.57606
Turicibacter_bilis	0.001031	0	0.00221	0	0.0454	0.09951
Unclassified	6.72212	6.869471	7.399285	5.066345	0.35446	0.45468
Undibacter_mobilis	0.000678	0.001087	0	0	0.1894	0.27751
Veillonella_atypica	1.504651	1.423999	1.875723	0.130836	0.04685	0.10187
Vescimonas_fastidiosa	0	0.001843	0	0.00871	0.0034	0.02209

Supplementary Table 4 Microbial composition of mice oral microbiota

Phylum	Con	Lig	WT	Mu	MuInd	p.value	FDR
Acidobacteria	0.5269	0.093	0.08299	0.06951	0.04467	0.01279	0.06756
Actinobacteria	1.98916	1.14143	1.86838	2.2355	1.51739	0.14719	0.26985
Armatimonadetes	0.00387	0.00074	0	0	0	0.1916	0.28146
Bacteroidetes	8.1268	3.15787	4.21951	6.66902	0.99449	0.19505	0.28146
Candidatus_Saccharibacteria	0.17633	0.11885	0.02826	0.09819	0.01349	0.01969	0.07174
Chlamydiae	0.00162	0.00037	0	0	0	0.1916	0.28146
Chloroflexi	0.13785	0.02641	0.00975	0.00994	0.00891	0.01536	0.06756
Cyanobacteria	0.52185	0.17592	0.21517	0.21203	0.16681	0.03768	0.09211
Deferribacteres	0.00065	0.00385	0.00154	0.00112	0	0.23054	0.29046
Deinococcus_Thermus	0.0039	0	0	0	0	0.0801	0.16021
Firmicutes	74.3585	53.8024	55.7558	53.2442	64.3863	0.0566	0.12453
Fusobacteria	0.00032	0.00191	0.0011	0.00328	0.00989	0.00798	0.06756
Gemmatimonadetes	0.01703	0.00223	0.00227	0.00128	0	0.00338	0.06756
Latescibacteria	0	0.00073	0	0	0	0.40601	0.40601
Nitrospirae	0.01027	0.00264	0.00363	0.00085	0.0008	0.33075	0.38297
Parcubacteria	0.00065	0	0	0	0	0.40601	0.40601
Planctomycetes	0.11981	0.02267	0.01411	0.0084	0.00524	0.02731	0.07511
Proteobacteria	13.655	41.3752	37.7607	37.3617	32.8311	0.01359	0.06756
Tenericutes	0	0.0015	0	0.00181	0	0.23765	0.29046
Unclassified	0.29253	0.0608	0.03269	0.0263	0.01694	0.02283	0.07174
Verrucomicrobia	0.05497	0.01158	0.00419	0.05692	0.00401	0.2047	0.28146

Genus	Con	Lig	WT	Mu	MuInd	p.value	FDR
Acetatifactor	0.17357	0.12716	0.01571	0.00782	0.00119	0.0105	0.06007
Acetoanaerobium	0.00098	0	0	0	0	0.40601	0.48544
Achromobacter	0	0	0.00205	0.00109	0.00115	0.70005	0.70517
Aciditerrimonas	0.01452	0.00037	0.00103	0	0	0.01021	0.06007
Acidobacterium	0.00602	0	0.00034	0.00116	0	0.02409	0.0818
Acidovorax	0.01061	0	0	0.00223	0.00041	0.27099	0.44077
Acinetobacter	0.06957	0.00225	0.06175	0.0349	0.0051	0.00577	0.05309
Actinoallomurus	0.02496	0.00744	0.0062	0.00243	0.00361	0.03348	0.10171
Actinocorallia	0.01143	0.0037	0.00068	0.00043	0	0.02035	0.07362
Actinomadura	0.02379	0.00185	0.00444	0.00294	0.00041	0.01219	0.06445
Actinomyces	0.00355	0.00718	0.0135	0.01602	0.03085	0.00197	0.05309
Actinoplanes	0.00225	0	0	0	0	0.01114	0.06007
Actinospica	0.00875	0.003	0.00073	0	0.00082	0.01086	0.06007
Aeribacillus	0.00064	0	0	0	0	0.40601	0.48544
Aeromicrobium	0.00194	0	0	0.00043	0	0.1916	0.3446
Aggregicoccus	0.00226	0	0	0.00041	0.00041	0.41052	0.48661
Akkermansia	0.0423	0.00639	0.00171	0.05605	0.00284	0.51543	0.57779
Alishewanella	0.0018	0	0	0.00043	0	0.53577	0.57779
Alistipes	0.23864	0.25866	0.11311	0.06868	0.00351	0.0189	0.0732
Alkanindiges	0	0	0.00068	0	0	0.40601	0.48544
Allobaculum	0.00568	1.33962	0.8864	2.86755	3.02748	0.00491	0.05309

Alloprevotella	0.19664	0.1517	0.0953	0.41327	0.00519	0.21867	0.36892
Altererythrobacter	0	0	0	0.00163	0	0.01114	0.06007
Anaerofilum	0.00065	0.00037	0	0.00036	0	0.70005	0.70517
Anaerofustis	0.00101	0	0	0.00145	0	0.23765	0.38936
Anaeroplasma	0	0.00037	0	0.00073	0	0.53577	0.57779
Anaerosalibacter	0.00098	0	0	0	0	0.40601	0.48544
Anaerotruncus	0.06286	0.09056	0.00658	0.00836	0.00081	0.13753	0.27823
Aquicella	0.00355	0.0011	0	0	0	0.21538	0.36562
Aquihabitans	0.00288	0	0	0	0	0.40601	0.48544
Aquisphaera	0.0447	0.00853	0.00842	0.00168	0.00041	0.01329	0.0677
Aridibacter	0.00193	0	0	0.0004	0	0.53577	0.57779
Armatimonadetes_gp4	0.00226	0	0	0	0	0.40601	0.48544
Armatimonadetes_gp5	0	0.00074	0	0	0	0.40601	0.48544
Asticcacaulis	0.004	0	0	0	0	0.01114	0.06007
Atopobium	0.00098	0	0	0	0	0.40601	0.48544
Aureimonas	0.00033	0.0011	0	0	0	0.53577	0.57779
Azospira	0.0013	0	0	0	0	0.40601	0.48544
Azospirillum	0.02007	0.00073	0.00075	0.00079	0.00076	0.00353	0.05309
Bacillus	0.01392	0.00037	0.00114	0.00588	0.00114	0.01857	0.0732
Bacteroides	0.2167	0.04523	0.18545	0.2446	0.03196	0.64403	0.67342
Barnesiella	2.3674	0.57494	0.58348	0.82951	0.01779	0.03178	0.09888
Bauldia	0.00392	0	0	0	0	0.01114	0.06007
Bdellovibrio	0.00098	0.00117	0	0.0013	0	0.70005	0.70517
Beijerinckia	0.00321	0	0	0	0.00041	0.21538	0.36562
Bifidobacterium	0.01437	0.64189	1.14227	0.61489	0.69161	0.02712	0.0883
Bilophila	0.00487	0	0	0	0	0.01114	0.06007
Blastocatella	0.00278	0	0	0	0	0.0801	0.17395
Blastococcus	0.00209	0.00186	0	0	0.00042	0.07464	0.17395
Blautia	0.07972	0.05852	0.00569	0.00992	0.00079	0.0149	0.06829
Bosea	0.00244	0	0	0.00041	0	0.05183	0.1332
Bradyrhizobium	0.10886	0.04413	0.01566	0.00587	0.00518	0.03675	0.10601
Brevibacterium	0.00161	0	0.00107	0.00155	0.00242	0.17555	0.33293
Brevundimonas	0.01375	0.00149	0.00292	0.00602	0.00117	0.00358	0.05309
Burkholderia	0.06803	0.01155	0.00714	0.00246	0.0004	0.00588	0.05309
Butyricicoccus	0.1834	0.09451	0.00794	0.01531	0.00119	0.00304	0.05309
Byssovorax	0	0	0.00103	0	0	0.40601	0.48544
Caldimonas	0.00097	0	0	0	0	0.40601	0.48544
Catenulispora	0.00693	0.01249	0.00185	0.00194	0	0.04805	0.12585
Cellulosilyticum	0.00482	0	0	0	0	0.0801	0.17395
Chitinophaga	0.00161	0.0055	0	0	0	0.53577	0.57779
Christensenella	0.00552	0.00914	0.01419	0.0156	0.00791	0.41955	0.49306
Chryseobacterium	0.00533	0.00113	0.00106	0.00213	0.0028	0.4442	0.51761
Chthonomonas	0.00161	0	0	0	0	0.40601	0.48544
Clostridium_III	0.01424	0.00632	0.0021	0.00218	0.00038	0.6202	0.65346
Clostridium_IV	0.23854	0.15144	0.02551	0.09834	0.00661	0.05328	0.13566
Clostridium_XVIII	0.05391	0.01067	0.00137	0.00364	0	0.01953	0.0732
Clostridium_XlVa	1.37183	1.35933	0.05897	0.33492	0.02543	0.00579	0.05309

Clostridium_XlVb	0.30483	0.11991	0.01387	0.04717	0.00274	0.08295	0.17548
Clostridium_sensu_stricto	0.011	0.00151	0.02742	0.01792	0	0.02729	0.0883
Collinsella	0.00194	0	0	0	0	0.0801	0.17395
Comamonas	0.03274	0.00407	0.00068	0.00549	0.00157	0.01917	0.0732
Conexibacter	0.00568	0.00111	0	0	0	0.00079	0.05309
Coprococcus	0.00578	0	0	0	0	0.0801	0.17395
Corynebacterium	1.19137	0.10519	0.24788	1.14857	0.27598	0.00599	0.05309
Coxiella	0.0029	0	0.00039	0	0	0.1916	0.3446
Cupriavidus	0.00064	0.00257	0	0	0.00042	0.70005	0.70517
Deinococcus	0.0039	0	0	0	0	0.0801	0.17395
Delftia	0.00915	0	0.00115	0.0029	0	0.00418	0.05309
Desulfovibrio	0.32546	0.28702	0.02749	0.03427	0.00524	0.02	0.07334
Devosia	0.0055	0	0.00106	0.00043	0.00079	0.31914	0.48544
Dialister	0.00129	0	0	0	0	0.40601	0.48544
Diaphorobacter	0.01144	0.00514	0.00214	0.00193	0	0.0197	0.0732
Diplorickettsia	0.24558	0.05413	0.00769	0.00825	0.0008	0.06263	0.15657
Dolosigranulum	0	0	0.00479	0.01249	0.0339	0.00171	0.05309
Dongia	0.00162	0.00039	0	0	0	0.1916	0.3446
Dorea	0.04846	0.02246	0.0011	0.01686	0.00079	0.04899	0.12711
Duganella	0.00517	0	0	0	0	0.00117	0.05309
Dyella	0.01841	0.03852	0.00205	0	0.00041	0.00679	0.05657
Dysgonomonas	0.00161	0.00147	0	0.00218	0	0.35557	0.48544
Edaphobacter	0.00129	0.00037	0	0	0.00042	0.70005	0.70517
Eggerthella	0.04134	0.01186	0.00175	0.00151	0.00082	0.72989	0.73256
Eisenbergiella	0.02177	0.04789	0.00485	0.00078	0	0.03504	0.1036
Elizabethkingia	0.00967	0.00227	0.00313	0.00429	0.00117	0.41516	0.49
Enhydrobacter	0.01033	0.00076	0.00293	0.00278	0.00117	0.02844	0.08989
Enterococcus	0.62562	0.14583	0.08139	0.2358	0.06687	0.00816	0.06007
Enterorhabdus	0.0371	0.0139	0.00818	0.01429	0.00394	0.31846	0.48544
Escherichia	4.15395	0.86573	0.8384	0.30002	0.13261	0.02547	0.08479
Ethanoligenens	0.00289	0	0	0.00079	0	0.53577	0.57779
Eubacterium	0.04154	0.02503	0.00415	0.0147	0.00117	0.03582	0.10478
Exiguobacterium	0.00097	0	0	0	0.00041	0.21538	0.36562
Faecalibacterium	0.00801	0.00111	0.00217	0.00257	0.00202	0.04757	0.12579
Faecalicoccus	0.04594	0.0094	0.00689	0.00327	0	0.07377	0.17395
Ferruginibacter	0	0	0.00137	0	0	0.40601	0.48544
Finegoldia	0.00064	0.00037	0.00155	0.00122	0.00122	0.67032	0.69825
Flavobacterium	0.00161	0.00037	0	0.00073	0	0.41052	0.48661
Flavonifractor	0.25171	0.12093	0.0083	0.01596	0.00162	0.00446	0.05309
Franconibacter	0.00265	0	0.00039	0	0	0.1916	0.3446
Fulvimonas	0.00488	0.00366	0.00356	0.00042	0	0.13784	0.27823
Fusobacterium	0	0.00039	0.00038	0.00209	0.00437	0.20353	0.35879
Gaiella	0.03685	0.00446	0.00155	0.00244	0.00123	0.00949	0.06007
Gemella	0.00258	0.00075	0.01408	0.02255	0.02426	0.0031	0.05309
Geminicoccus	0.00097	0	0	0	0	0.40601	0.48544
Gemmata	0.01495	0.00297	0.00073	0.00256	0.00038	0.10313	0.21484
Gemmatimonas	0.01703	0.00223	0.00227	0.00128	0	0.00338	0.05309

Gemmiger	0.00205	0.00443	0.00038	0	0	0.27247	0.44077
Geobacter	0.00129	0	0	0	0	0.40601	0.48544
Geodermatophilus	0.02366	0.00373	0.00142	0.00442	0.00038	0.01309	0.0677
Georgfuchsia	0.00065	0.00037	0.00034	0	0	0.70005	0.70517
Gordonibacter	0.00234	0.00037	0.00034	0	0	0.11261	0.2311
Granulicatella	0.00362	0.00076	0.00374	0.00541	0.02196	0.00413	0.05309
Haemophilus	0.00202	0.00495	0.00543	0.01036	0.02432	0.00337	0.05309
Halomonas	0.01135	0.00187	0.00415	0.00073	0	0.03403	0.10171
Helicobacter	0.46448	0.35189	0.33557	0.04037	0.01361	0.10775	0.22278
Herbaspirillum	0.00033	0.00073	0	0	0	0.53577	0.57779
Herpetosiphon	0.00072	0	0	0	0	0.40601	0.48544
Iamia	0.00793	0.00073	0	0.00036	0.00041	0.04271	0.11699
Intestinimonas	0.03773	0.03215	0.00217	0.03875	0.00078	0.01814	0.0732
Janthinobacterium	0.07069	0.00781	0.01222	0.02192	0.00664	0.01705	0.07105
Kaistia	0	0.00332	0	0	0	0.0801	0.17395
Kofleria	0.00735	0.00147	0.00068	0.00043	0.00079	0.35304	0.48544
Kribbella	0.00162	0	0.00038	0	0	0.21538	0.36562
Kroppenstedtia	0.0013	0.00037	0	0	0	0.21538	0.36562
Ktedonobacter	0.03249	0.00888	0.00329	0.00043	0.00124	0.01114	0.06007
Kurthia	0.00586	0	0	0	0	0.01114	0.06007
Kutzneria	0.00259	0.00037	0	0	0	0.04297	0.11699
Labilithrix	0.01415	0.00558	0.00192	0.00202	0	0.01934	0.0732
Lachnospiracea_incertae_sedis	0.00161	0.00186	0	0.00145	0	0.33308	0.48544
Lactobacillus	14.1438	2.77908	12.9618	4.57959	0.71722	0.02117	0.0749
Lactococcus	0.11948	0.01592	0.0123	0.01398	0.01575	0.01557	0.0696
Latescibacteria	0	0.00073	0	0	0	0.40601	0.48544
Lautropia	0.00065	0.00227	0.00333	0.00503	0.0072	0.00543	0.05309
Legionella	0.00144	0	0	0	0	0.40601	0.48544
Leifsonia	0.00065	0	0	0	0	0.40601	0.48544
Leptotrichia	0.00032	0.00152	0.00073	0.00119	0.00552	0.00862	0.06007
Leucobacter	0.00292	0	0.00068	0.00036	0.00038	0.55199	0.59033
Luteimonas	0.00129	0	0	0	0	0.40601	0.48544
Lysinibacillus	0.00097	0	0	0	0	0.40601	0.48544
Marvinbryantia	0.03227	0.01502	0.00038	0.0015	0	0.07267	0.17395
Massilia	0.00889	0.00222	0.00171	0.00162	0	0.01669	0.07062
Megamonas	0.00494	0.00038	0	0	0.00038	0.08033	0.17395
Mesorhizobium	0.01542	0.0011	0.00141	0.00043	0.00038	0.02559	0.08479
Metallibacterium	0.00161	0.00039	0	0	0	0.1916	0.3446
Methylobacterium	0.02136	0.00414	0.00709	0.00534	0.00116	0.19172	0.3446
Methyloceanibacter	0.00162	0	0.00113	0	0	0.21538	0.36562
Methyloferula	0.00594	0.00111	0	0.00036	0.00038	0.16178	0.31112
Methylophilus	0.00176	0	0	0	0	0.0801	0.17395
Microbacterium	0.005	0.00222	0.0015	0.00241	0	0.08161	0.17397
Micrococcus	0.00233	0.00038	0	0.00208	0.00513	0.01007	0.06007
Mogibacterium	0.01007	0.00346	0	0.00216	0	0.29156	0.46888
Moraxella	0	0	0.05493	0.05996	0.07216	0.00177	0.05309
Mucilaginibacter	0.01335	0.00333	0.00034	0.00043	0.00041	0.00457	0.05309

Mucispirillum	0.00065	0.00385	0.00154	0.00112	0	0.23054	0.38657
Mycobacterium	0.21968	0.01264	0.00682	0.01328	0.038	0.13923	0.27823
Neisseria	0.00226	0.0049	0.01638	0.01846	0.03404	0.00079	0.05309
Nevskia	0.00293	0.00037	0	0	0.00042	0.70005	0.70517
Nitrospira	0.01027	0.00264	0.00363	0.00085	0.0008	0.33075	0.48544
Nitrospirillum	0.00097	0	0.00038	0	0	0.53577	0.57779
Nocardia	0.01761	0.00337	0.00501	0.00166	0.00122	0.03378	0.10171
Nocardioides	0.00354	0.00037	0	0	0	0.53577	0.57779
Novosphingobium	0.00098	0	0	0	0	0.40601	0.48544
Oceanobacillus	0.00293	0	0.00039	0.00073	0	0.70005	0.70517
Odoribacter	0.0596	0.0884	0.02756	0.02583	0.00038	0.3123	0.48544
Oligoflexus	0.0013	0	0	0	0	0.40601	0.48544
Olsenella	0.0039	0.11994	0.23321	0.21639	0.31462	0.02125	0.0749
Oscillibacter	0.25495	0.11573	0.00997	0.01577	0.00079	0.08102	0.17397
Paenibacillus	0.00097	0	0.00068	0	0	0.53577	0.57779
Pantoea	4.8014	0.08266	0.08079	0.09664	0.10725	0.36582	0.48544
Parabacteroides	0.03411	0.00553	0.00077	0.00717	0.00041	0.19067	0.3446
Paracoccus	0.00618	0.0015	0.00112	0.00036	0.00038	0.48489	0.56027
Paraprevotella	0.00161	0.00038	0.00141	0.0401	0	0.19388	0.34564
Parasutterella	0.03149	0.0053	0.00372	0.02146	0.00122	0.55383	0.59033
Parcubacteria	0.00065	0	0	0	0	0.40601	0.48544
Parvibacter	0.02485	0.01047	0	0.00972	0.00038	0.00361	0.05309
Pasteurella	0.115	39.0884	35.7686	35.9183	32.1382	0.01471	0.06829
Pediococcus	0.00422	0.00153	0	0.00043	0.00082	0.54189	0.58211
Pedomicrobium	0.00163	0.00037	0.00038	0	0.00038	0.88181	0.88181
Pelomonas	0.03491	0.00674	0.00653	0.00722	0.00395	0.01641	0.07062
Peptoniphilus	0.00032	0.00115	0	0.0004	0.0004	0.62744	0.65857
Phascolarctobacterium	0.00228	0.00037	0.00034	0	0	0.70005	0.70517
Phenylobacterium	0.03119	0.00996	0.0019	0.0013	0.00042	0.0056	0.05309
Plesiomonas	0.00228	0.00074	0.00078	0	0	0.46757	0.54254
Polaromonas	0.00195	0	0	0.00086	0.00042	0.377	0.48544
Porphyromonas	0.00033	0.00073	1.34919	0.59009	0.1231	0.00574	0.05309
Povalibacter	0.00129	0.00039	0	0.00041	0	0.15123	0.29494
Prevotella	0.35495	0.12554	0.18654	1.05856	0.01394	0.42395	0.49611
Propionibacterium	0.04582	0.10205	0.12685	0.11556	0.11371	0.1635	0.31223
Propionicimonas	0.00065	0	0	0	0	0.40601	0.48544
Proteus	0.48104	0.01113	0.02304	0.14432	0.04262	0.03941	0.10946
Pseudochrobactrum	0.00269	0.00147	0.00038	0.00161	0.00038	0.59746	0.63193
Pseudokineococcus	0.00252	0	0	0	0	0.40601	0.48544
Pseudolabrys	0.00226	0.00037	0.00103	0	0.00038	0.58912	0.62551
Pseudomonas	1.38602	0.21896	0.23285	0.39704	0.13031	0.01467	0.06829
Pseudonocardia	0.00964	0.00076	0	0	0	0.1432	0.28129
Pseudoxanthomonas	0.00129	0	0	0	0	0.40601	0.48544
Ralstonia	0.01641	0.00037	0.00218	0.00084	0.0016	0.00522	0.05309
Reyranella	0.01394	0.00257	0.00141	0.00086	0.00076	0.51012	0.57779
Rheinheimera	0.00064	0	0	0	0	0.40601	0.48544
Rhizobium	0.01163	0.00484	0.00175	0.00207	0.0012	0.14063	0.27823

Rhizomicrobium	0.00948	0.00149	0.00153	0.0008	0.00041	0.01422	0.06829
Rhodanobacter	0.0017	0	0	0	0	0.0801	0.17395
Rhodomicrobium	0.00033	0.00117	0	0	0	0.53577	0.57779
Rickettsia	0	0.00078	0	0	0	0.40601	0.48544
Rikenella	0.02268	0.02501	0.0085	0.00258	0.0004	0.23787	0.38936
Romboutsia	0.00421	0	0	0	0	0.01114	0.06007
Roseburia	0.03358	0.11202	0.00116	0.00604	0.00198	0.04502	0.12019
Roseiarcus	0.00976	0.0041	0.00183	0	0.00038	0.0714	0.17395
Roseiflexus	0.00163	0	0	0	0	0.40601	0.48544
Roseomonas	0.00194	0.00184	0	0	0	0.23765	0.38936
Rothia	0.00065	0.00187	0.02619	0.02658	0.023	0.00405	0.05309
Rubellimicrobium	0.00259	0	0.00073	0.00181	0	0.16091	0.31112
Rubrobacter	0.00032	0.00074	0	0	0	0.53577	0.57779
Rudaea	0.00032	0.0044	0	0	0	0.53577	0.57779
Ruminococcus	0.08831	0.04119	0.00103	0.01154	0	0.01569	0.0696
Ruminococcus2	0.00226	0.0011	0.00112	0.00041	0	0.32281	0.48544
Saccharibacteria	0.17633	0.11885	0.02826	0.09819	0.01349	0.01969	0.0732
Salmonella	0.2448	0.05797	0.00982	0.00396	0.03965	0.032	0.09888
Sandaracinus	0.00133	0	0	0	0	0.01114	0.06007
Scardovia	0	0	0.00527	0.00242	0	0.00634	0.05448
Schlegelella	0.00289	0	0	0.00043	0	0.53577	0.57779
Serratia	0.00533	0.00038	0.00077	0.00123	0.00279	0.10034	0.21063
Singulisphaera	0.00618	0.00073	0	0	0.00038	0.01645	0.07062
Sinomonas	0.00227	0	0.00068	0.00043	0	0.34565	0.48544
Skermanella	0.00108	0	0	0	0	0.40601	0.48544
Solirubrobacter	0.0107	0.00115	0.00282	0.00043	0	0.07294	0.17395
Soonwooa	0.00108	0	0	0	0	0.40601	0.48544
Spartobacteria	0.00973	0.00297	0.00248	0.00043	0.00117	0.03904	0.10946
Sphingobacterium	0.00197	0	0.00103	0.00109	0	0.19482	0.34564
Sphingomonas	0.04652	0.00852	0.00901	0.01259	0.00742	0.02284	0.07851
Sporobacter	0.0029	0.00155	0	0	0	0.06065	0.15302
Staphylococcus	0.22446	0.06775	0.04018	0.0308	0.01602	0.07856	0.17395
Stella	0.00064	0	0	0	0	0.40601	0.48544
Stenotrophomonas	0.0145	0.00267	0.00034	0.00245	0.00041	0.00294	0.05309
Steroidobacter	0.00065	0	0	0	0	0.40601	0.48544
Streptococcus	51.2123	4.65251	6.85188	12.2618	10.5446	0.00801	0.06007
Streptomyces	0.02024	0.00302	0.00038	0.00232	0.00042	0.07007	0.17359
Tatumella	0.02355	0.00037	0.00038	0.00043	0.00079	0.30148	0.48201
Telmatospirillum	0.00097	0.0011	0	0	0	0.23765	0.38936
Tetragenococcus	0.00161	0	0	0	0	0.40601	0.48544
Thermicanus	0	0	0	0	0.00153	0.40601	0.48544
Thermogemmatispora	0.0054	0	0	0	0	0.40601	0.48544
Tumebacillus	0.00285	0	0	0	0	0.0801	0.17395
Turicibacter	0.116	0.05686	0.00264	0.13363	0.00732	0.00219	0.05309
Vagococcus	0.00161	0	0	0	0	0.40601	0.48544
Vampirovibrio	0.01757	0.00642	0.00145	0.00576	0.0004	0.38197	0.48544
Veillonella	0.10533	38.9823	34.3344	31.5563	49.7664	0.00501	0.05309

Weissella	0.01977	0.00637	0.00103	0.00254	0.00306	0.03701	0.10601
Xanthomonas	0.00515	0.0042	0.00708	0.00721	0.0016	0.40349	0.48544

**Species**	**Con**	**Lig**	**WT**	**Mu**	**MuInd**	**p.value**	**FDR**
Acetatifactor_muris	0.17357	0.12716	0.01571	0.00782	0.00119	0.0105	0.06073
Acetoanaerobium_noterae	0.00098	0	0	0	0	0.40601	0.49074
Achromobacter_spanius	0	0	0.00205	0.00109	0.00115	0.70005	0.71027
Aciditerrimonas_ferrireducens	0.01452	0.00037	0.00103	0	0	0.01021	0.06073
Acidobacterium_capsulatum	0.00602	0	0.00034	0.00116	0	0.02409	0.09303
Acinetobacter_guillouiae	0.00532	0	0	0.00388	0.0008	0.00931	0.06073
Acinetobacter_johnsonii	0.05826	0.00188	0.05418	0.02616	0.00431	0.04855	0.13898
Acinetobacter_lwoffii	0.0047	0	0.00526	0.00332	0	0.03086	0.10707
Actinocorallia_glomerata	0.01006	0.00187	0.00034	0	0	0.01216	0.06451
Actinomyces_odontolyticus	0	0.00076	0.00377	0.00622	0.01126	0.00325	0.05524
Actinomyces_oris	0.00355	0.00642	0.00974	0.0098	0.01959	0.01077	0.06073
Actinospica_robiniae	0.00875	0.003	0.00073	0	0.00082	0.01086	0.06073
Aeribacillus_pallidus	0.00064	0	0	0	0	0.40601	0.49074
Aggregicoccus_edonensis	0.00226	0	0	0.00041	0.00041	0.41052	0.49405
Akkermansia_muciniphila	0.0423	0.00639	0.00171	0.05605	0.00284	0.51543	0.5773
Alistipes_finegoldii	0.00072	0.00073	0.00034	0.01139	0	0.21151	0.35854
Alistipes_indistinctus	0.00522	0.00039	0	0.00073	0	0.01645	0.07495
Alistipes_onderdonkii	0.04987	0.07808	0.04675	0.01959	0.00079	0.13937	0.26397
Alistipes_shahii	0.00613	0.00522	0.00415	0	0.00041	0.31197	0.48233
Alkanindiges_illinoisensis	0	0	0.00068	0	0	0.40601	0.49074
Allobaculum_stercoricanis	0.00568	1.33962	0.8864	2.86755	3.02748	0.00491	0.06073
Alloprevotella_rava	0.19664	0.1517	0.0953	0.41327	0.00519	0.21867	0.36185
Altererythrobacter_troitsensis	0	0	0	0.00163	0	0.01114	0.06073
Anaerofustis_stercorihominis	0.00101	0	0	0.00145	0	0.23765	0.38004
Anaeroplasma_abactoclasticum	0	0.00037	0	0.00073	0	0.53577	0.5773
Anaerosalibacter_bizertensis	0.00098	0	0	0	0	0.40601	0.49074
Anaerotruncus_colihominis	0.06286	0.09056	0.00658	0.00836	0.00081	0.13753	0.26397
Aquicella_siphonis	0.00194	0.0011	0	0	0	0.23765	0.38004
Aquihabitans_daechungensis	0.00288	0	0	0	0	0.40601	0.49074
Aquisphaera_giovannonii	0.0447	0.00853	0.00842	0.00168	0.00041	0.01329	0.06844
Aridibacter_famidurans	0.00193	0	0	0.0004	0	0.53577	0.5773
Asticcacaulis_excentricus	0.004	0	0	0	0	0.01114	0.06073
Atopobium_rimae	0.00098	0	0	0	0	0.40601	0.49074
Azospira_oryzae	0.0013	0	0	0	0	0.40601	0.49074
Bacillus_amyloliquefaciens	0.0029	0	0.00076	0	0.00114	0.05125	0.14125
Bacteroides_faecichinchillae	0.01174	0.00037	0	0.00073	0	0.11261	0.22522
Bacteroides_fragilis	0.14601	0.01544	0.00248	0.00165	0.00042	0.08013	0.17683
Bacteroides_plebeius	0.00194	0.00038	0	0	0	0.1916	0.335
Bacteroides_sartorii	0.01557	0.00495	0.0743	0.00158	0.00287	0.09536	0.20236
Bacteroides_uniformis	0.02115	0.00076	0	0	0	0.14727	0.27663
Barnesiella_intestinihominis	1.27728	0.3085	0.32997	0.54925	0.01066	0.06142	0.16263
Bauldia_consociata	0.00392	0	0	0	0	0.01114	0.06073
Beijerinckia_indica	0.00321	0	0	0	0.00041	0.21538	0.35854
Bifidobacterium_catenulatum	0.01015	0	0.00107	0.00123	0.00202	0.051	0.14125

Bifidobacterium_longum	0.00129	0	0	0	0	0.0801	0.17683
Bifidobacterium_pseudolongum	0.00293	0.64189	1.14119	0.61366	0.68959	0.02157	0.08447
Bilophila_wadsworthia	0.00487	0	0	0	0	0.01114	0.06073
Blastocatella_fastidiosa	0.00278	0	0	0	0	0.0801	0.17683
Blautia_hydrogenotrophica	0.01868	0.00463	0.00145	0.00463	0.0004	0.48577	0.56268
Bosea_vestrisii	0.00244	0	0	0.00041	0	0.05183	0.14125
Brevundimonas_lenta	0.00193	0	0	0.0004	0	0.04297	0.12969
Burkholderia_sabiae	0.02679	0.00633	0.00385	0.00084	0	0.02696	0.10033
Burkholderia_soli	0.00713	0.00259	0.00034	0.00036	0.0004	0.31197	0.48233
Burkholderia_symbiotica	0.00195	0	0	0	0	0.0801	0.17683
Burkholderia_zhejiangensis	0.02146	0.00037	0.00257	0.00083	0	0.00702	0.06073
Butyricicoccus_pullicaecorum	0.1834	0.09451	0.00794	0.01531	0.00119	0.00304	0.05524
Byssovorax_cruenta	0	0	0.00103	0	0	0.40601	0.49074
Caldimonas_manganoxidans	0.00097	0	0	0	0	0.40601	0.49074
Catenulispora_graminis	0.00693	0.01249	0.00185	0.00194	0	0.04805	0.13898
Cellulosilyticum_ruminicola	0.00482	0	0	0	0	0.0801	0.17683
Christensenella_minuta	0.00552	0.00914	0.01419	0.0156	0.00791	0.41955	0.50058
Chryseobacterium_lactis	0.00533	0.00113	0.00106	0.00213	0.0028	0.4442	0.52548
Clostridium_aldenense	0.03123	0.00417	0.00221	0.00128	0	0.13063	0.25756
Clostridium_colinum	0.05018	0.03327	0.00107	0.01262	0.00038	0.05174	0.14125
Clostridium_lactatifermentans	0.25465	0.08664	0.0128	0.03455	0.00236	0.06829	0.17578
Clostridium_leptum	0.13774	0.10109	0.01323	0.07919	0.00621	0.0999	0.20661
Clostridium_methylpentosum	0.0252	0.00952	0.00112	0.00117	0	0.00902	0.06073
Clostridium_moniliforme	0	0	0.02703	0.01588	0	0.23765	0.38004
Clostridium_ramosum	0.05391	0.01067	0.00137	0.00364	0	0.01953	0.08052
Clostridium_sardiniense	0.00616	0.00073	0.00039	0.00204	0	0.13958	0.26397
Clostridium_scindens	0.46379	0.42364	0.01736	0.11631	0.01445	0.00783	0.06073
Clostridium_sporosphaeroides	0.0013	0	0	0	0	0.0801	0.17683
Clostridium_viride	0.07366	0.04084	0.01117	0.01799	0.0004	0.05267	0.14216
Collinsella_aerofaciens	0.00194	0	0	0	0	0.0801	0.17683
Comamonas_jiangduensis	0.00419	0	0	0.00036	0	0.04297	0.12969
Comamonas_thiooxydans	0.02855	0.00407	0.00068	0.00512	0.00157	0.02741	0.10033
Conexibacter_arvalis	0.00568	0.00111	0	0	0	0.00079	0.04932
Coprococcus_comes	0.00578	0	0	0	0	0.0801	0.17683
Corynebacterium_durum	0.00097	0.00379	0.00806	0.00713	0.009	0.03176	0.10718
Corynebacterium_lubricantis	0	0.00074	0	0.00254	0	0.53577	0.5773
Corynebacterium_mastitidis	1.18578	0.09767	0.23129	1.12348	0.25222	0.00532	0.06073
Corynebacterium_matruchotii	0	0.00111	0.00479	0.00525	0.00193	0.08052	0.17683
Corynebacterium_simulans	0.00301	0.00076	0	0.00043	0.00041	0.03004	0.10572
Coxiella_burnetii	0.0029	0	0.00039	0	0	0.1916	0.335
Deinococcus_aquiradiocola	0.00097	0	0	0	0	0.0801	0.17683
Deinococcus_ficus	0.00098	0	0	0	0	0.40601	0.49074
Deinococcus_gobiensis	0.00195	0	0	0	0	0.40601	0.49074
Devosia_insulae	0.0055	0	0.00106	0.00043	0.00079	0.31914	0.48586
Dialister_invisus	0.00129	0	0	0	0	0.40601	0.49074
Diaphorobacter_nitroreducens	0.01144	0.00514	0.00214	0.00193	0	0.0197	0.08052
Diplorickettsia_massiliensis	0.24558	0.05413	0.00769	0.00825	0.0008	0.06263	0.16425

Dolosigranulum_pigrum	0	0	0.00479	0.01249	0.0339	0.00171	0.04932
Dongia_mobilis	0.00162	0.00039	0	0	0	0.1916	0.335
Dorea_longicatena	0.04846	0.02246	0.0011	0.01686	0.00079	0.04899	0.13898
Duganella_sacchari	0.00517	0	0	0	0	0.00117	0.04932
Dysgonomonas_oryzarvi	0.00161	0.00147	0	0.00218	0	0.35557	0.49074
Edaphobacter_modestus	0.00129	0.00037	0	0	0.00042	0.70005	0.71027
Eggerthella_lenta	0.04134	0.01186	0.00175	0.00151	0.00082	0.72989	0.73684
Eisenbergiella_tayi	0.02177	0.04789	0.00485	0.00078	0	0.03504	0.11428
Elizabethkingia_miricola	0.00967	0.00227	0.00313	0.00429	0.00117	0.41516	0.49748
Enterococcus_saccharolyticus	0.62562	0.14583	0.08139	0.2358	0.06687	0.00816	0.06073
Enterorhabdus_caecimuris	0.0371	0.0139	0.00818	0.01429	0.00394	0.31846	0.48586
Escherichia	4.15395	0.86573	0.8384	0.30002	0.13261	0.02547	0.09701
Ethanoligenens_harbinense	0.00289	0	0	0.00079	0	0.53577	0.5773
Eubacterium_callanderi	0.01757	0.00911	0.00342	0.00173	0.00038	0.2498	0.39682
Eubacterium_coprostanoligenes	0.01996	0.01294	0.00073	0.01182	0.00079	0.45593	0.53707
Eubacterium_eligens	0.00161	0.00186	0	0.00145	0	0.33308	0.49074
Faecalibacterium_prausnitzii	0.00801	0.00111	0.00217	0.00257	0.00202	0.04757	0.13898
Faecalicoccus_pleomorphus	0.0363	0.00602	0.00616	0.00327	0	0.25633	0.40488
Ferruginibacter_lapsinanis	0	0	0.00137	0	0	0.40601	0.49074
Finegoldia_magna	0.00064	0.00037	0.00155	0.00122	0.00122	0.67032	0.70056
Flavonifractor_plautii	0.25171	0.12093	0.0083	0.01596	0.00162	0.00446	0.06073
Franconibacter_pulveris	0.00265	0	0.00039	0	0	0.1916	0.335
Fulvimonas_soli	0.00488	0.00366	0.00356	0.00042	0	0.13784	0.26397
Fusobacterium_nucleatum	0	0.00039	0.00038	0.00209	0.00437	0.20353	0.35144
Gaiella_occulta	0.03685	0.00446	0.00155	0.00244	0.00123	0.00949	0.06073
Gemella_haemolysans	0.00258	0.00075	0.01408	0.02255	0.02426	0.0031	0.05524
Geminicoccus_roseus	0.00097	0	0	0	0	0.40601	0.49074
Gemmata_obscuriglobus	0.01495	0.00297	0.00073	0.00256	0.00038	0.10313	0.20926
Gemmatimonas_aurantiaca	0.01703	0.00223	0.00227	0.00128	0	0.00338	0.05524
Gemmiger_formicilis	0.00205	0.00443	0.00038	0	0	0.27247	0.42795
Georgfuchsia_toluolica	0.00065	0.00037	0.00034	0	0	0.70005	0.71027
Gordonibacter_pamelaeae	0.00234	0.00037	0.00034	0	0	0.11261	0.22522
Granulicatella_adiacens	0.00161	0.00076	0.00221	0.00289	0.0104	0.0145	0.07176
Granulicatella_elegans	0.00201	0	0.00153	0.00252	0.01156	0.00278	0.05524
Haemophilus_aegyptius	0.00137	0	0.00428	0.00655	0.00523	0.02123	0.08431
Haemophilus_parainfluenzae	0.00064	0.00495	0.00115	0.00381	0.01909	0.00136	0.04932
Halomonas_hamiltonii	0.01135	0.00187	0.00415	0.00073	0	0.03403	0.11261
Helicobacter_ganmani	0.44677	0.34815	0.33557	0.04037	0.01208	0.08142	0.17683
Helicobacter_hepaticus	0.01771	0.00375	0	0	0.00153	0.08091	0.17683
Herbaspirillum_huttiense	0.00033	0.00073	0	0	0	0.53577	0.5773
Herpetosiphon_geysericola	0.00072	0	0	0	0	0.40601	0.49074
Iamia_majanohamensis	0.00793	0.00073	0	0.00036	0.00041	0.04271	0.12969
Intestinimonas_butyriciproducens	0.03773	0.03215	0.00217	0.03875	0.00078	0.01814	0.07981
Kaistia_defluvii	0	0.00332	0	0	0	0.0801	0.17683
Kofleria_flava	0.00735	0.00147	0.00068	0.00043	0.00079	0.35304	0.49074
Kribbella_catacumbae	0.00162	0	0.00038	0	0	0.21538	0.35854
Kroppenstedtia_eburnea	0.0013	0.00037	0	0	0	0.21538	0.35854

Ktedonobacter_racemifer	0.03249	0.00888	0.00329	0.00043	0.00124	0.01114	0.06073
Kurthia_gibsonii	0.00586	0	0	0	0	0.01114	0.06073
Kutzneria_kofuensis	0.00259	0.00037	0	0	0	0.04297	0.12969
Labilithrix_luteola	0.01415	0.00558	0.00192	0.00202	0	0.01934	0.08052
Lactobacillus_apodemi	13.4451	2.36428	12.8243	3.98141	0.68365	0.01819	0.07981
Lactobacillus_aviarius	0.08201	0.04546	0.00278	0.0017	0.00123	0.73154	0.73684
Lactobacillus_delbrueckii	0.01067	0.02821	0.00137	0.00086	0.00119	0.34157	0.49074
Lactobacillus_fermentum	0.03131	0.01553	0.00183	0.00083	0	0.04339	0.12969
Lactobacillus_intestinalis	0.00068	0.00037	0.00077	0.00508	0	0.75394	0.75666
Lactobacillus_paracasei	0	0	0.00076	0.00119	0.00284	0.07385	0.17683
Lactobacillus_plantarum	0.32406	0.10352	0.03997	0.0412	0.00081	0.09903	0.20661
Lactobacillus_rossiae	0.00032	0.0096	0.00038	0	0	0.70005	0.71027
Lactococcus_garvieae	0.05611	0.00074	0.00153	0.00654	0.01575	0.13063	0.25756
Lactococcus_lactis	0.06337	0.01518	0.01077	0.00744	0	0.09694	0.20416
Lautropia_mirabilis	0.00065	0.00227	0.00333	0.00503	0.0072	0.00543	0.06073
Leptotrichia_hongkongensis	0.00032	0.00152	0.00038	0	0.00038	0.21151	0.35854
Luteimonas_cucumeris	0.00129	0	0	0	0	0.40601	0.49074
Lysinibacillus_xylanilyticus	0.00097	0	0	0	0	0.40601	0.49074
Marvinbryantia_formatexigens	0.03227	0.01502	0.00038	0.0015	0	0.07267	0.17683
Massilia_aerilata	0.00298	0.00147	0.00137	0.00083	0	0.15876	0.29424
Massilia_flava	0.00161	0.00038	0	0	0	0.53577	0.5773
Metallibacterium_scheffleri	0.00161	0.00039	0	0	0	0.1916	0.335
Methylobacterium_bullatum	0.00445	0.00186	0.00111	0.00159	0.00038	0.76602	0.76602
Methylobacterium_dankookense	0.01395	0.00152	0.00077	0.00079	0.00078	0.08312	0.17912
Methylobacterium_komagatae	0.00166	0.00037	0.00312	0.00218	0	0.56473	0.60151
Methylobacterium_tarhaniae	0.0013	0.00039	0.00209	0.00078	0	0.59847	0.63021
Methyloceanibacter_caenitepidi	0.00162	0	0.00113	0	0	0.21538	0.35854
Methyloferula_stellata	0.00594	0.00111	0	0.00036	0.00038	0.16178	0.29785
Methylophilus_luteus	0.00176	0	0	0	0	0.0801	0.17683
Micrococcus_aloeverae	0.00233	0.00038	0	0.00208	0.00513	0.01007	0.06073
Moraxella_nonliquefaciens	0	0	0.05493	0.05996	0.07216	0.00177	0.04932
Moraxella_osloensis	0.01033	0.00076	0.00293	0.00278	0.00117	0.02844	0.10136
Mucispirillum_schaedleri	0.00065	0.00385	0.00154	0.00112	0	0.23054	0.37923
Mycobacterium_avium	0.21968	0.01264	0.00682	0.01328	0.038	0.13923	0.26397
Neisseria_macacae	0.00097	0.00188	0.00706	0.01238	0.02197	0.00075	0.04932
Neisseria_oralis	0.00097	0.00189	0.0019	0.00041	0.00041	0.49801	0.57447
Neisseria_perflava	0.00032	0.00113	0.00742	0.00568	0.01166	0.00107	0.04932
Nevskia_terrae	0.00293	0.00037	0	0	0.00042	0.70005	0.71027
Nitrospira_moscoviensis	0.01027	0.00264	0.00363	0.00085	0.0008	0.33075	0.49074
Nitrospirillum_amazonense	0.00097	0	0.00038	0	0	0.53577	0.5773
Nocardioides_hwasunensis	0.00354	0.00037	0	0	0	0.53577	0.5773
Novosphingobium_rosa	0.00098	0	0	0	0	0.40601	0.49074
Oceanobacillus_indicireducens	0.00293	0	0.00039	0.00073	0	0.70005	0.71027
Odoribacter_laneus	0.0596	0.0884	0.02756	0.02583	0.00038	0.3123	0.48233
Oligoflexus_tunisiensis	0.0013	0	0	0	0	0.40601	0.49074
Olsenella_profusa	0.00097	0	0	0	0	0.40601	0.49074
Oscillibacter_ruminantium	0.01135	0.00453	0	0.00109	0	0.06529	0.16962

Oscillibacter_valericigenes	0.16056	0.07469	0.00539	0.01205	0.00038	0.10107	0.20661
Paenibacillus_koleovorans	0.00097	0	0	0	0	0.40601	0.49074
Pantoea_stewartii	4.8014	0.08266	0.08079	0.09664	0.10725	0.36582	0.49074
Parabacteroides_distasonis	0.01525	0.00296	0.00077	0.00717	0.00041	0.44284	0.52548
Parabacteroides_merdae	0.01886	0.00257	0	0	0	0.0091	0.06073
Paracoccus_chinensis	0.00618	0.0015	0.00112	0.00036	0.00038	0.48489	0.56268
Paraprevotella_clara	0.00161	0.00038	0.00141	0.0401	0	0.19388	0.33687
Parasutterella_excrementihominis	0.03149	0.0053	0.00372	0.02146	0.00122	0.55383	0.59218
Parvibacter_caecicola	0.02485	0.01047	0	0.00972	0.00038	0.00361	0.05572
Pasteurella_pneumotropica	0.115	39.0884	35.7686	35.9183	32.1382	0.01471	0.07176
Pediococcus_pentosaceus	0.00422	0.00153	0	0.00043	0.00082	0.54189	0.58164
Pelomonas_aquatica	0.03491	0.00674	0.00653	0.00722	0.00395	0.01641	0.07495
Peptoniphilus_gorbachii	0.00032	0.00115	0	0.0004	0.0004	0.62744	0.65822
Phascolarctobacterium_faecium	0.00228	0.00037	0.00034	0	0	0.70005	0.71027
Phenylobacterium_koreense	0.00814	0.00111	0.00076	0.00086	0	0.01506	0.0722
Phenylobacterium_muchangponense	0.01668	0.00662	0.00076	0.00043	0.00042	0.0109	0.06073
Planktothricoides_raciborskii	0.00064	0	0	0	0	0.40601	0.49074
Plesiomonas_shigelloides	0.00228	0.00074	0.00078	0	0	0.46757	0.54615
Porphyromonas_catoniae	0	0	0.00039	0.00085	0.00277	0.02743	0.10033
Porphyromonas_gingivalis	0.00033	0.00073	1.3488	0.58924	0.12033	0.0123	0.06451
Povalibacter_uvarum	0.00129	0.00039	0	0.00041	0	0.15123	0.28215
Prevotella_copri	0.00128	0	0	0	0.00038	0.53577	0.5773
Prevotella_scopos	0	0	0.00152	0.00173	0.0059	0.00627	0.06073
Propionibacterium_acnes	0.04582	0.10205	0.12685	0.11556	0.11371	0.1635	0.29903
Propionicimonas_paludicola	0.00065	0	0	0	0	0.40601	0.49074
Pseudochrobactrum_asaccharolyticum	0.00269	0.00147	0.00038	0.00161	0.00038	0.59746	0.63021
Pseudokineococcus_lusitanus	0.00252	0	0	0	0	0.40601	0.49074
Pseudolabrys_taiwanensis	0.00226	0.00037	0.00103	0	0.00038	0.58912	0.62509
Pseudomonas_aeruginosa	1.37959	0.21633	0.22994	0.39621	0.12991	0.01548	0.07294
Pseudomonas_psychrotolerans	0.00643	0.00263	0.00291	0.00084	0.00041	0.31983	0.48586
Pseudonocardia_endophytica	0.00288	0	0	0	0	0.40601	0.49074
Pseudoxanthomonas_mexicana	0.00129	0	0	0	0	0.40601	0.49074
Ralstonia_pickettii	0.01641	0.00037	0.00218	0.00084	0.0016	0.00522	0.06073
Reyranella_massiliensis	0.00421	0.00073	0.00103	0.00043	0.00038	0.36733	0.49074
Reyranella_soli	0.00973	0.00184	0.00038	0.00043	0.00038	0.69501	0.71027
Rheinheimera_texasensis	0.00064	0	0	0	0	0.40601	0.49074
Rhizobium_jaguaris	0.00841	0.00408	0.00137	0.00126	0.00041	0.13726	0.26397
Rhizobium_larrymoorei	0.00322	0.00076	0.00038	0.00081	0.00079	0.46509	0.54555
Rhizomicrobium_electricum	0.00948	0.00149	0.00153	0.0008	0.00041	0.01422	0.07176
Rhodanobacter_glycinis	0.0017	0	0	0	0	0.0801	0.17683
Rhodomicrobium_vannielii	0.00033	0.00117	0	0	0	0.53577	0.5773
Rikenella_microfusus	0.02268	0.02501	0.0085	0.00258	0.0004	0.23787	0.38004
Romboutsia_sedimentorum	0.00421	0	0	0	0	0.01114	0.06073
Roseburia_faecis	0.02136	0.09899	0.00038	0.00309	0.00157	0.01837	0.07981
Roseiarcus_fermentans	0.00976	0.0041	0.00183	0	0.00038	0.0714	0.17683
Roseiflexus_castenholzii	0.00163	0	0	0	0	0.40601	0.49074
Roseomonas_gilardii	0.00032	0.0011	0	0	0	0.1916	0.335

Roseomonas_soli	0	0.00073	0	0	0	0.40601	0.49074
Rothia_aeria	0.00065	0.00074	0.00221	0.00376	0.00909	0.00658	0.06073
Rothia_mucilaginosa	0	0.00113	0.02398	0.02282	0.01391	0.0016	0.04932
Rubellimicrobium_aerolatum	0.00259	0	0.00073	0	0	0.0467	0.13811
Rubellimicrobium_thermophilum	0	0	0	0.00181	0	0.40601	0.49074
Rudaea_cellulosilytica	0.00032	0.0044	0	0	0	0.53577	0.5773
Ruminococcus_albus	0.00097	0	0	0.00042	0	0.53577	0.5773
Ruminococcus_bromii	0.00064	0	0	0	0	0.40601	0.49074
Ruminococcus_champanellensis	0.0867	0.04119	0.00103	0.01113	0	0.02104	0.08431
Ruminococcus_faecis	0.00226	0.0011	0.00112	0.00041	0	0.32281	0.48772
Salmonella_enterica	0.2448	0.05797	0.00982	0.00396	0.03965	0.032	0.10718
Sandaracinus_amylolyticus	0.00133	0	0	0	0	0.01114	0.06073
Scardovia_wiggsiae	0	0	0.00527	0.00242	0	0.00634	0.06073
Schlegelella_aquatica	0.00289	0	0	0.00043	0	0.53577	0.5773
Serratia_myotis	0.00533	0.00038	0.00077	0.00123	0.00279	0.10034	0.20661
Singulisphaera_rosea	0.00098	0.00037	0	0	0	0.53577	0.5773
Skermanella_xinjiangensis	0.00108	0	0	0	0	0.40601	0.49074
Solirubrobacter_ginsenosidimutans	0.0052	0.00076	0.00107	0	0	0.03535	0.11428
Solirubrobacter_soli	0.00161	0	0.00034	0	0	0.53577	0.5773
Soonwooa_buanensis	0.00108	0	0	0	0	0.40601	0.49074
Sphingobacterium_siyangense	0.00064	0	0	0	0	0.40601	0.49074
Sphingomonas_jaspsi	0.0211	0.0022	0.00213	0.00043	0.00118	0.02836	0.10136
Sphingomonas_oligophenolica	0.02542	0.00632	0.00687	0.01216	0.00624	0.08726	0.1866
Sporobacter_termitidis	0.0029	0.00155	0	0	0	0.06065	0.16213
Staphylococcus_cohnii	0.22446	0.06775	0.04018	0.0308	0.01602	0.07856	0.17683
Stenotrophomonas_maltophilia	0.01281	0.00228	0.00034	0.00245	0.00041	0.00297	0.05524
Stenotrophomonas_nitritireducens	0.00169	0.00039	0	0	0	0.1916	0.335
Streptococcus_acidominimus	0	0.00537	0.00385	0.00911	0.01469	0.01904	0.08052
Streptococcus_danieliae	51.198	4.63325	6.69402	12.1178	10.362	0.00885	0.06073
Streptococcus_oralis	0.01269	0.01125	0.10044	0.09144	0.14097	0.00176	0.04932
Streptococcus_salivarius	0.00161	0.00263	0.05356	0.04347	0.02694	0.00129	0.04932
TM7_phylum	0.14864	0.11149	0.02655	0.0974	0.01349	0.0312	0.10707
Telmatospirillum_siberiense	0.00097	0.0011	0	0	0	0.23765	0.38004
Tetragenococcus_halophilus	0.00161	0	0	0	0	0.40601	0.49074
Thermicanus_aegyptius	0	0	0	0	0.00153	0.40601	0.49074
Thermogemmatispora_onikobensis	0.0054	0	0	0	0	0.40601	0.49074
Tumebacillus_ginsengisoli	0.00285	0	0	0	0	0.0801	0.17683
Turicibacter_sanguinis	0.116	0.05686	0.00264	0.13363	0.00732	0.00219	0.05524
Vampirovibrio_chlorellavorus	0.01757	0.00642	0.00145	0.00576	0.0004	0.38197	0.49074
Veillonella_dispar	0.00258	0.00492	0.00525	0.01935	0.04541	0.00898	0.06073
Weissella_confusa	0.01977	0.00637	0.00103	0.00254	0.00306	0.03701	0.11826
Xanthomonas_citri	0.00515	0.0042	0.00708	0.00721	0.0016	0.40349	0.49074

The ARRIVE guidelines 2.0: author checklist

The ARRIVE Essential 10

These items are the basic minimum to include in a manuscript. Without this information, readers and reviewers cannot assess the reliability of the findings.

Item		Recommendation	Section/line number, or reason for not reporting
**Study design**	1	For each experiment, provide brief details of study design including:<br>a. The groups being compared, including control groups. If no control group has been used, the rationale should be stated.<br>b. The experimental unit (e.g. a single animal, litter, or cage of animals).	Methods/Infection and treatment of mice/ Page 27
**Sample size**	2	a. Specify the exact number of experimental units allocated to each group, and the total number in each experiment. Also indicate the total number of animals used.<br>b. Explain how the sample size was decided. Provide details of any *a priori* sample size calculation, if done.	Methods/Infection and treatment of mice/ Page 27
**Inclusion and exclusion criteria**	3	a. Describe any criteria used for including and excluding animals (or experimental units) during the experiment, and data points during the analysis. Specify if these criteria were established *a priori*. If no criteria were set, state this explicitly.<br>b. For each experimental group, report any animals, experimental units or data points not included in the analysis and explain why. If there were no exclusions, state so.<br>c. For each analysis, report the exact value of *n* in each experimental group.	Methods/Infection and treatment of mice/ Page 27
**Randomisation**	4	a. State whether randomisation was used to allocate experimental units to control and treatment groups. If done, provide the method used to generate the randomisation sequence.<br>b. Describe the strategy used to minimise potential confounders such as the order of treatments and measurements, or animal/cage location. If confounders were not controlled, state this explicitly.	Methods/Infection and treatment of mice/ Page 27
**Blinding**	5	Describe who was aware of the group allocation at the different stages of the experiment (during the allocation, the conduct of the experiment, the outcome assessment, and the data analysis).	Methods/Infection and treatment of mice/Page 27 Author Contributions/ Page 31
**Outcome measures**	6	a. Clearly define all outcome measures assessed (e.g. cell death, molecular markers, or behavioural changes).<br>b. For hypothesis-testing studies, specify the primary outcome measure, i.e. the outcome measure that was used to determine the sample size.	Methods/Page 27-28
**Statistical methods**	7	a. Provide details of the statistical methods used for each analysis, including software used.<br>b. Describe any methods used to assess whether the data met the assumptions of the statistical approach, and what was done if the assumptions were not met.	Methods/ Bioinformatic and statistical analysis/ Page 29
**Experimental animals**	8	a. Provide species-appropriate details of the animals used, including species, strain and substrain, sex, age or developmental stage, and, if relevant, weight.<br>b. Provide further relevant information on the provenance of animals, health/immune status, genetic modification status, genotype, and any previous procedures.	Methods/Infection and treatment of mice/ Page 27
**Experimental procedures**	9	For each experimental group, including controls, describe the procedures in enough detail to allow others to replicate them, including:<br>a. What was done, how it was done and what was used.<br>b. When and how often.<br>c. Where (including detail of any acclimatisation periods).<br>d. Why (provide rationale for procedures).	Methods/Infection and treatment of mice/ Page 27
**Results**	10	For each experiment conducted, including independent replications, report:<br>a. Summary/descriptive statistics for each experimental group, with a measure of variability where applicable (e.g. mean and SD, or median and range).<br>b. If applicable, the effect size with a confidence interval.	Results/Page 11-14 Supplementary Table 4

Submission Checklist

#	Checklist Item	Description	Checked
**1**	Author Details		√
**2**	Abstract	Research articles should include an Abstract, without subheadings, and up to 150 words in length.	√
**3**	Introduction		√
**4**	Results		√
**5**	Discussion		√
**6**	Methods	All descriptions of Methods should be provided in the main manuscript file.	√
**7**	Declaration Statements	Declaration statements should include: (1) Data Availability; (2) Code Availability (where applicable); (3) Acknowledgements (note: statement should declare any Funding for the work); (4) Author Contributions; (5) Competing Interests.	√
**8**	References		√
**9**	Figures, Tables and associated legends		√
**10**	Supplementary information	The supplementary information should be uploaded separately in a single, merged PDF. Supplementary items must be labeled and cited using only the following formats: Supplementary Figure 1, Supplementary Table 1, etc.	√
**11**	ARRIVE Essential 10 checklist	The ARRIVE Essential 10 checklist should be submitted as a supplementary file.	√
